# Blood eosinophil count and airway epithelial transcriptome relationships in COPD versus asthma

**DOI:** 10.1111/all.14016

**Published:** 2019-09-10

**Authors:** Leena George, Adam R. Taylor, Anna Esteve‐Codina, María Soler Artigas, Gian Andri Thun, Stewart Bates, Stelios Pavlidis, Scott Wagers, Anne Boland, Antje Prasse, Piera Boschetto, David G. Parr, Adam Nowinski, Imre Barta, Jens Hohlfeld, Timm Greulich, Maarten van den Berge, Pieter S. Hiemstra, Wim Timens, Timothy Hinks, Sally Wenzel, Salman Siddiqui, Matthew Richardson, Per Venge, Simon Heath, Ivo Gut, Martin D. Tobin, Lindsay Edwards, John H. Riley, Ratko Djukanovic, Charles Auffray, Bertrand De‐Meulder, Sven Erik‐Dahlen, Ian M. Adcock, Kian Fan Chung, Loems Ziegler‐Heitbrock, Peter J. Sterk, Dave Singh, Christopher E. Brightling, the U‐BIOPRED and the EvA study teams

**Affiliations:** ^1^ Institute for Lung Health, Leicester NIHR Biomedical Research Centre University of Leicester Leicester UK; ^2^ GSK Respiratory Therapeutic Area Unit Stevenage UK; ^3^ Centre for Genomic Regulation CNAG‐CRG Centre Nacional d'Anàlisi Genòmica, Barcelona Institute for Science and Technology Barcelona Spain; ^4^ Psychiatric Genetics Unit, Group of Psychiatry, Mental Health and Addiction Vall d'Hebron Research Institute (VHIR), Universitat Autònoma de Barcelona Barcelona Spain; ^5^ Instituto de Salud Carlos III Biomedical Network Research Centre on Mental Health (CIBERSAM) Barcelona Spain; ^6^ Airway Disease Section National Heart & Lung Institute, Imperial College London London UK; ^7^ Data Science Institute Imperial College London London UK; ^8^ Biosci Consulting Maasmechelen Belgium; ^9^ Institut de Génomique, CEA CNG Centre National de Génotypage Evry France; ^10^ Department of Pneumology University Medical Center Freiburg Germany; ^11^ Department of Medical Sciences University of Ferrara and Ferrara City Hospital Ferrara Italy; ^12^ Department of Respiratory Medicine University Hospitals Coventry and Warwickshire NHS Trust Coventry UK; ^13^ Department of Respiratory Medicine National Institute of Tuberculosis and Lung Diseases Warsaw Poland; ^14^ Department of Pathophysiology National Koranyi Institute for TB and Pulmonology Budapest Hungary; ^15^ Fraunhofer Institute for Toxicology and Experimental Medicine Hannover Germany; ^16^ Department of Medicine, Pulmonary and Critical Care Medicine University Medical Center Giessen and Marburg, Philipps‐Universität Marburg Marburg Germany; ^17^ Member of the German Center for Lung Research (DZL) Großhansdorf Germany; ^18^ Department of Pulmonary Diseases University Medical Center Groningen, University of Groningen Groningen The Netherlands; ^19^ Department of Pulmonary Diseases Leiden University Medical Center, University of Leiden Leiden The Netherlands; ^20^ Department of Pathology and Medical Biology University Medical Center Groningen, University of Groningen Groningen The Netherlands; ^21^ University of Oxford Oxford UK; ^22^ Department of Medicine University of Pittsburgh Pittsburgh PA USA; ^23^ Department of Immunology University of Pittsburgh Pittsburgh PA USA; ^24^ Department of Medical Sciences, Clinical Chemistry Uppsala University Uppsala Sweden; ^25^ Universitat Pompeu Fabra Barcelona Spain; ^26^ NIHR Southampton Respiratory Biomedical Research Unit and Clinical and Experimental Sciences Southampton UK; ^27^ European Institute for Systems Biology and Medicine (EISBM) CNRS‐ENS‐UCBL, Université de Lyon Lyon cedex 07 France; ^28^ Karolinska Institute Stockholm Sweden; ^29^ EvA Study Center Helmholtz Zentrum Muenchen and Asklepios‐Klinik Gauting Germany; ^30^ Department Respiratory Medicine Amsterdam University Medical Centres, University of Amsterdam Amsterdam The Netherlands; ^31^ Centre for Respiratory Medicine and Allergy The University of Manchester Manchester UK; ^32^ Medicines Evaluation Unit University Hospital of South Manchester NHS Foundation Trust Manchester UK

**Keywords:** asthma, chronic obstructive pulmonary disease, eosinophil, gene expression, T2‐immunity

## Abstract

**Background:**

Whether the clinical or pathophysiologic significance of the “treatable trait” high blood eosinophil count in COPD is the same as for asthma remains controversial. We sought to determine the relationship between the blood eosinophil count, clinical characteristics and gene expression from bronchial brushings in COPD and asthma.

**Methods:**

Subjects were recruited into a COPD (emphysema versus airway disease [EvA]) or asthma cohort (Unbiased BIOmarkers in PREDiction of respiratory disease outcomes, U‐BIOPRED). We determined gene expression using RNAseq in EvA (n = 283) and Affymetrix microarrays in U‐BIOPRED (n = 85). We ran linear regression analysis of the bronchial brushings transcriptional signal versus blood eosinophil counts as well as differential expression using a blood eosinophil > 200 cells/μL as a cut‐off. The false discovery rate was controlled at 1% (with continuous values) and 5% (with dichotomized values).

**Results:**

There were no differences in age, gender, lung function, exercise capacity and quantitative computed tomography between eosinophilic versus noneosinophilic COPD cases. Total serum IgE was increased in eosinophilic asthma and COPD. In EvA, there were 12 genes with a statistically significant positive association with the linear blood eosinophil count, whereas in U‐BIOPRED, 1197 genes showed significant associations (266 positive and 931 negative). The transcriptome showed little overlap between genes and pathways associated with blood eosinophil counts in asthma versus COPD. Only CST1 was common to eosinophilic asthma and COPD and was replicated in independent cohorts.

**Conclusion:**

Despite shared “treatable traits” between asthma and COPD, the molecular mechanisms underlying these clinical entities are predominately different.

## Graphical abstract

In a chronic obstructive pulmonary disease (COPD) cohort (EvA, n = 283), 12 genes, whereas in asthma cohort (UBIOPRED, n = 85), 1197 genes in bronchial epithelial brushes were correlated with a blood eosinophil count. The gene *CST1* was common to eosinophilic asthma and COPD and was replicated in independent cohorts. Despite shared “treatable traits” between asthma and COPD, the molecular mechanisms underlying these clinical entities are predominately different.

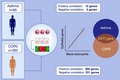

## 1 INTRODUCTION

There is increasing recognition that airway inflammation in chronic obstructive pulmonary disease (COPD) is heterogeneous.^1^, ^2^, ^3^ Although COPD is typically associated with neutrophilic inflammation in 10%‐40% of subjects, there is evidence of eosinophilic inflammation. The aetiology of eosinophilic inflammation in COPD and the underlying immunopathological mechanisms in eosinophilic COPD are poorly understood.^1^, ^2^, ^3^

In asthma, an eosinophilia is associated with increased allergic sensitization and T2‐mediated immunity.^4^ However, atopy is not essential and type‐2 innate lymphoid cells (ILC2) have emerged as an alternative mechanisms driving an airway eosinophilia.^4^ In COPD, cytokines associated with type‐2‐mediated immunity such as interleukin (IL)‐5 and CCL17 are increased in sputum in subjects with eosinophilic inflammation.^5^, ^6^, ^7^ In contrast to noneosinophilic COPD, the sputum concentrations of the pro‐inflammatory cytokines IL‐1β and TNF are increased versus eosinophilic COPD.^5^, ^6^, ^7^ Atopy is not a prominent feature of COPD,^1^, ^2^, ^3^ and whether cytokines released by the bronchial epithelium that activate ILC2 cells such as the “alarmin” IL‐33 and thymic stromal lymphopoietin (TSLP) are increased in eosinophilic COPD is uncertain. Interestingly, the airway bacterial ecology is different between eosinophilic and noneosinophilic COPD with a lower proportion of *Proteobacteria* versus *Firmicutes* in eosinophilic COPD.^8^

These findings suggest that damage to the epithelium or functional differences in the bronchial epithelium might underlie the different inflammatory responses observed such as eosinophilic and noneosinophilic inflammation in COPD and asthma. It has also been proposed that the presence of eosinophilic inflammation in COPD and asthma is driven by similar molecular pathways leading to an eosinophilic phenotype representing a common “treatable trait”.^3^, ^9^, ^10^ Therefore, we hypothesized that the gene profiles of bronchial epithelial brushes will be distinct in both COPD and asthma subjects between those subjects with or without eosinophilic inflammation, but will show similarities between eosinophilic COPD versus asthma.

## 2 MATERIALS AND METHODS

The EvA and U‐BIOPRED studies have been described previously.^11^, ^12^, ^13^, ^14^ EvA is a multicentre study of COPD across 5 European countries (Germany, UK, Italy, Hungary and Poland) that involves clinical examination, CT imaging and bronchoscopic sampling. U‐BIOPRED is a 12‐month prospective European‐wide industry‐academic collaborative study designed to identify sub‐phenotypes of asthma patients. At baseline, a subgroup underwent bronchial biopsy collection which included bronchial brushings. The studies were approved by the relevant ethics and review boards at the participating centres and all subjects provided written informed consent.

### 2.1 Clinical assessment in EvA

In the EvA study, a diagnosis of COPD was based on a postbronchodilator forced expiratory volume in 1 second (FEV_1_)/forced vital capacity (FVC) ratio < 70%. Patients were excluded if they had very severe COPD (FEV_1_ < 30% predicted or <1 L), bronchodilator reversibility greater than 400 mL, had smoked within the previous 12 months, or had a primary diagnosis of bronchiectasis, asthma or any other significant respiratory diseases. All subjects underwent pulmonary function testing, six‐minute walk distance (6MWD), quantification of dyspnoea using the modified Medical Research Council (mMRC) scale, thoracic CT using a standardized acquisition and analysis,^9^ blood sampling, sputum induction and bronchoscopy. Bronchial brush samples were taken from the right upper and lower lobes (5 mm bristle diameter; Olympus). The brush samples were transferred into RNAprotect immediately and stored at −20°C. Sputum samples were processed to derive a differential cell count read by a single‐blinded observer and a supernatant. The concentration of the neutrophil mediators myeloperoxidase (MPO) and human neutrophil lipocalin (HNL) and the eosinophil mediator eosinophil cationic peroxidase (ECP) were measured by ELISA (Uppsala, Sweden) in sputum supernatants.

### 2.2 Bronchial epithelial brush sample RNAseq in EvA

Samples were extracted using the AllPrep DNA/RNA Mini Kit (Qiagen) on a Qiacube robot. RNA quality was tested by running the samples on a Bioanalyzer 2100 from Agilent, using the RNA6000 Nano LabChip kit (Agilent Technologies, Inc), and cDNA libraries were prepared on Sciclone robot (PerkinElmer Inc) using the RNATRUSEQ protocol (Illumina Inc). RNAseq was performed using Hiseq 2000 with 100 bp paired‐end reads. Samples with low sequencing throughput (<10 M reads) were removed from the analysis. The selected RNAseq samples were aligned with the GEMTools RNAseq pipeline v1.7 (http://gemtools.github.io).^15^ The transcriptome was generated from version 15 of the Gencode annotation. After mapping, all alignments were filtered to increase the number of uniquely mapping reads. The filter criteria contained a minimum intron length of 20, a minimum exon overlap of 5 and a filter step against the reference annotation checking for consistent pairs and junctions where both sides align to the same annotated gene. Quantifications and read counts were calculated using the Flux Capacitor^15^ to create gene‐level read counts that were used for the differential expression analysis.

### 2.3 Clinical assessment in U‐BIOPRED

Within U‐BIOPRED, there are three asthmatic cohorts: severe nonsmoking asthma (cohort A), smoking and ex‐smoking asthmatics with >5 pack years (cohort B) and nonsmoking mild‐moderate asthma (cohort C). The current study consisted of a combination of cross‐sectional U‐BIOPRED participants from cohorts A and C. Participants in the cohorts were assessed with clinical and molecular measurements at baseline, using prespecified protocols.^13^ A subgroup of participants from each cohort underwent bronchial biopsy collection, which included bronchial brushings. In total, bronchial brushings from 49 cohort A patients and 36 cohort C patients were included in the present analysis. Bronchial brushings were obtained from the lower lobes; brushes were immediately spun down in PBS before the pellet was preserved in RNAlater^®^ solution and maintained at −70°C.

### 2.4 Bronchial epithelial brush gene array in U‐BIOPRED

RNA was extracted using Qiagen miRNeasy kit (Qiagen) and amplified with NuGen ovation pico WTA kit (NuGen Technologies). The cDNA was analysed using the Affymetrix HG‐U133 + PM microarray platform (Affymetrix). In U‐BIOPRED, CEL files were normalized, assessed for quality control to exclude technical outliers (chip image analysis, Affymetrix GeneChip QC, RNA degradation analysis, distribution analysis, principal components analysis, and correlation analysis) and normalized using the robust multi‐array (RMA) method.^16^ Low‐intensity probe sets (defined as having a maximum median group intensity <2^5^) were removed prior to reporting.

### 2.5 Replication cohorts

Genes that were associated with the blood eosinophil count and met the 1% false discovery rate (FDR) (adjusted *P* < .01) for both asthma and COPD were tested in available replication cohorts. Bronchial brush gene array (Affymetrix HG‐U133 or Human Gene 1.0 ST) and matching blood eosinophil data were available from a pooled analysis of asthma from four studies BOBCAT, Leicester, HINKS and SARP (n = 213)^17^, ^18^, ^19^ and from GLUCOLD (n = 79)^20^ for COPD.

### 2.6 Statistics

All analyses were carried out in R 3.3 or GraphPad PRISM 7. Comparisons between groups of subjects were made using Student's *t* test and Mann‐Whitney U test for normally and non‐normally distributed data, respectively, and correlations between clinical characteristics were assessed by Pearson's or Spearman correlation coefficient. We determined the relationship between clinical characteristics and blood eosinophil counts, either as continuous variables or dichotomized using 200 cells/μL blood as cut‐offs, in COPD and asthma cases. We selected this cut‐off as a value within the range (150‐300 cell/µL) used for blood eosinophil cut‐offs to predict response to therapies in asthma and COPD.^2^, ^21^ Differential gene expression analysis in COPD or asthma using the same blood cell cut‐off was undertaken, and additional linear regression analysis with the blood eosinophil concentration as a continuous variable was performed, both using either limma (microarray data) or limma‐voom (RNAseq data) (tools in R). FDR according to Benjamini and Hochberg was used to correct for multiple testing, and association results with a corrected *P*‐value < .05 (for models with dichotomized eosinophilic levels) and *P* < .01 (for models with linear levels) were considered significant. All models were run without any covariates. Additional adjustment for gender and recruiting centre was carried out in sensitivity analyses for models with linear eosinophilic concentrations. In the replication approach, the Pearson correlation coefficient was determined between gene expression levels and linear blood eosinophil concentration. Only genes that were associated with the blood eosinophil count and met FDR for both asthma and COPD were assessed in the replication cohorts. Pathway analysis was undertaken using R v3.3.3, running the metabaser v4.2.3 package (proprietary R packages supplied by Thompson Reuters), querying the MetaBase version 6.36.69400.

## 3 RESULTS

In the EvA cohort, 458 COPD subjects were recruited. Their clinical characteristics with the subjects dichotomized by blood (>200 eosinophils/μL) cut‐offs are shown in Table 1. There were no significant differences between those subjects with or without eosinophilic COPD with respect to demographics, spirometry, lung volumes, diffusion, CT‐derived densitometry nor %WA. Total serum IgE and serum ECP were increased in those subjects with higher blood eosinophil counts (Table 1). The blood eosinophil count was correlated with age, FEV_1_% predicted, total IgE and sputum eosinophil count (Table 2). The sputum eosinophil count was correlated with sputum ECP (Table S1). There was no significant difference in the proportions treated with inhaled corticosteroids in the noneosinophilic versus eosinophilic groups (58% vs 46%).

**Table 1:** Clinical characteristics of eosinophilic versus noneosinophilic COPD subjects using blood eosinophil cut‐off >200 eosinophils/μL in the whole EvA cohort (n = 458)

	Eosinophilic COPD	N	Noneosinophilic COPD	N	*P*‐value
Gender (male [n])	118	158	191	300	**.022**
Age (years)	64 (1)	158	65 (0)	300	.343
Smoking history (pack years)	41 (2)	158	40 (1)	300	.703
BMI kg/m^2^	28 (0.4)	158	28 (0.3)	300	.293
6MWD (m)	460 (9)	152	445 (6)	282	.165
BODE index	2 (0)	158	2 (0)	300	.538
Pulmonary function tests					
FEV_1_% predicted	69 (0)	157	73 (0)	300	.064
FEV_1_/FVC %	55 (1)	157	56 (1)	300	.613
Bronchodilator response (%)	9 (0.7)	157	9 (0.4)	299	.371
RV/TLC % predicted	1.24 (0.02)	157	1.24 (0.01)	290	.952
TLCO/VA % predicted	85 (0.02)	155	83 (0.02)	291	.586
CT parameters					
Lung density Perc15 HU	−916 (2)	140	−919 (1)	251	.155
Percentage wall area	65 (0.57)	139	64 (0.44)	273	.370
Blood parameters					
Blood leucocytes 10^9^cells/L	8 (0.1)	158	7 (0.1)	299	**.002**
Blood neutrophils 10^9^cells/L	4.9 (0.1)	158	4.6 (0.1)	299	.087
Blood eosinophils 10^9^cells/L	0.39 (0.023)	158	0.12 (0.003)	299	**<.001**
Blood IgE kU/L a	73 (29‐174)	153	36 (14‐94)	294	**<.001**
Sputum parameters					
Sputum neutrophils (%) a	71 (54‐84)	92	78 (66‐86)	164	**.019**
Sputum eosinophils (%) a	3.5 (0.75‐8.5)	92	1.25 (0.25‐4)	164	**<.001**
Sputum MPO (pg/mL)^a^	437 (164‐588)	68	337 (166‐595)	109	.798
Sputum HNL (pg/mL)^a^	1674 (627‐3213)	69	1480 (565‐2570)	108	.658
Sputum ECP (pg/mL)^a^	314 (103‐912)	69	164 (62‐506)	109	**.032**

Bold values indicate *P* < 0.05

Abbreviations: 6MWD, 6‐min walk distance; BMI, body mass index; BODE, body mass index, airflow obstruction, dyspnoea, exercise; CT, computed tomography; ECP, eosinophil cationic protein; FEV1, forced expiratory volume in 1s; FVC, forced vital capacity; HNL, human neutrophil lipocalin; HU, Hounsfield unit; IgE, immunoglobulin E; MPO, myeloperoxidase; Perc15, 15th percentile point; RV, residual volume; TLC, total lung capacity; TLCO, transfer capacity of the lungs for carbon monoxide; VA, alveolar volume.

a Mean (SEM) unless otherwise stated; median (interquartile range).

**Table 2:** Correlation of clinical characteristics with blood eosinophils in EvA (n = 458)

	Blood eosinophil correlation *r*	*P* value	N
Age (years)	−.102	**.029**	458
Smoking history (pack years)	.007	.886	458
BMI kg/m^2^	.040	.396	458
6MWD (m)	.125	**.009**	434
BODE index	.013	.777	458
Pulmonary function tests			
FEV_1_% predicted	−.099	**.034**	457
FEV_1_/FVC %	−.026	.582	457
Bronchodilator response (%)	.055	.237	456
RV/TLC % predicted	.048	.307	447
TLCO/VA % predicted	.011	.817	446
CT parameters			
Lung density Perc15 HU	.058	.256	391
Percentage wall area	−.010	.839	412
Blood parameters			
Blood leucocytes 10^9^cells/L	.068	.145	457
Blood neutrophils 10^9^cells/L	.030	.528	457
Blood IgE kU/L^a^	.234	**<.001**	447
Sputum parameters			
Sputum neutrophils (%)^a^	−.167	**.007**	256
Sputum eosinophils (%)^a^	.280	**<.001**	256
Sputum MPO (pg/mL)^a^	−.027	.721	177
Sputum HNL (pg/mL)^a^	−.014	.851	177
Sputum ECP (pg/mL)^a^	.161	**.031**	178

Bold values indicate *P* < 0.05

Abbreviations: 6MWD, 6‐min walk distance; BMI, body mass index; BODE, body mass index, airflow obstruction, dyspnoea, exercise; CT, computed tomography; ECP, eosinophil cationic protein; FEV1, forced expiratory volume in 1s; FVC, forced vital capacity; HNL, human neutrophil lipocalin; HU, Hounsfield unit; IgE, immunoglobulin E; MPO, myeloperoxidase; Perc15, 15th percentile point; RV, residual volume; TLC, total lung capacity; TLCO, transfer capacity of the lungs for carbon monoxide; VA, alveolar volume.

a Pearson correlation unless stated Spearman correlation.

There were assessable RNAseq data from bronchial brushes in 283 EvA subjects (RNAseq uploaded to European genome‐phenome archive: EGAD00001002003 and EGAD00001002004). The clinical characteristics were similar to the entire dataset (Table S2). The clinical characteristics of the U‐BIOPRED subjects are shown in Table S3. Total IgE and bronchodilator reversibility were higher in those with versus those without a blood eosinophil count >200 cells/μL. All of the asthma subjects were treated with inhaled corticosteroids. There were assessable gene array data from bronchial brushes in all 85 U‐BIOPRED subjects (gene array uploaded to gene expression omnibus: GSE76226). RNA quality was not different between the EvA and U‐BIOPRED cohorts. After removing lowly expressed genes, 20 143 genes were left for RNAseq differential expression analysis for EvA and 17 175/54 675 (31%) probe sets were detected in the U‐BIOPRED samples. Gender and centre were identified as potential confounders in the EvA data set, but not U‐BIOPRED and were therefore used in the sensitivity analysis.

No genes in the EvA COPD cohort and 356 genes in the U‐BIOPRED asthma cohorts were differentially expressed (FDR < 0.05) in bronchial brush samples between subjects with and without a blood eosinophil count > 200/μL. The 10 most highly differentiated genes are shown in Table 3a (COPD) and b (asthma), and all the differentially expressed genes that met the FDR criteria are shown in Table S4.

**Table 3:** (a) COPD top (b) Asthma top ten differentially expressed genes between individuals with high (>200 eosinophils/μL) and low blood eosinophil counts, ranked by expression fold change

Gene symbol	Gene expression fold change	*P* value	*P* value FDR corrected
(a)			
*TTTY15*	3.68	1.5E‐04	.26
*NLGN4Y*	3.48	1.3E‐04	.26
*CLCA1*	2.98	1.0E‐05	.14
*PSMA6P1*	2.80	1.8E‐04	.26
*TBL1Y*	2.77	1.8E‐04	.26
*FETUB*	2.69	1.0E‐05	.14
*IL9R*	2.22	1.5E‐04	.26
*CLC*	2.19	1.8E‐04	.26
*GPRC5D*	1.51	9.0E‐05	.26
*SH3RF2*	1.20	1.7E‐04	.26
(b)			
*S100A8*	2.5	2.6E‐04	3.5E‐02
*SRGN*	2.3	5.2E‐04	4.4E‐02
*IGK///IGKC*	2.2	1.7E‐04	3.4E‐02
*TPSB2*	2.2	1.8E‐04	3.5E‐02
*MNDA*	2.1	4.7E‐04	4.2E‐02
*ALOX5AP*	2.1	2.3E‐04	3.5E‐02
*TPSAB1*	2.1	1.7E‐04	3.4E‐02
*LCP1*	2.1	3.3E‐04	3.7E‐02
*SLC25A37*	2.0	7.9E‐04	4.7E‐02
*RGS2*	2.0	2.4E‐05	2.1E‐02

Abbreviations: ALOX5AP, arachidonate 5‐lipoxygenase‐activating protein; CLC, Charcot‐Leyden crystal galectin; CLCA1, calcium‐activated chloride channel protein 1; FETUB, fetuin‐B; GPRC5D, G‐protein‐coupled receptor family C group 5 member D; IGK, immunoglobulin kappa locus; IL9R, interleukin‐9 receptor; LCP1, lymphocyte cytosolic protein 1; MNDA, myeloid cell nuclear differentiation antigen; NLGN4Y, neuroligin 4, Y‐Linked; PSMA6P1, proteasome subunit alpha 6 pseudogene 1; RGS2, regulator of G‐protein signalling; S100A8, S100 calcium‐binding protein A8; SH3RF2, SH3 domain containing ring finger 2; SLC25A337, solute carrier family 25 member 37; SRGN, serglycin; TBL1Y, transducin‐beta‐like protein 1; TBSB2, tryptase beta 2; TPSAB1, tryptase alpha/beta 1; TTTY15, testis‐specific transcript, Y‐linked 15.

**Figure 1:**
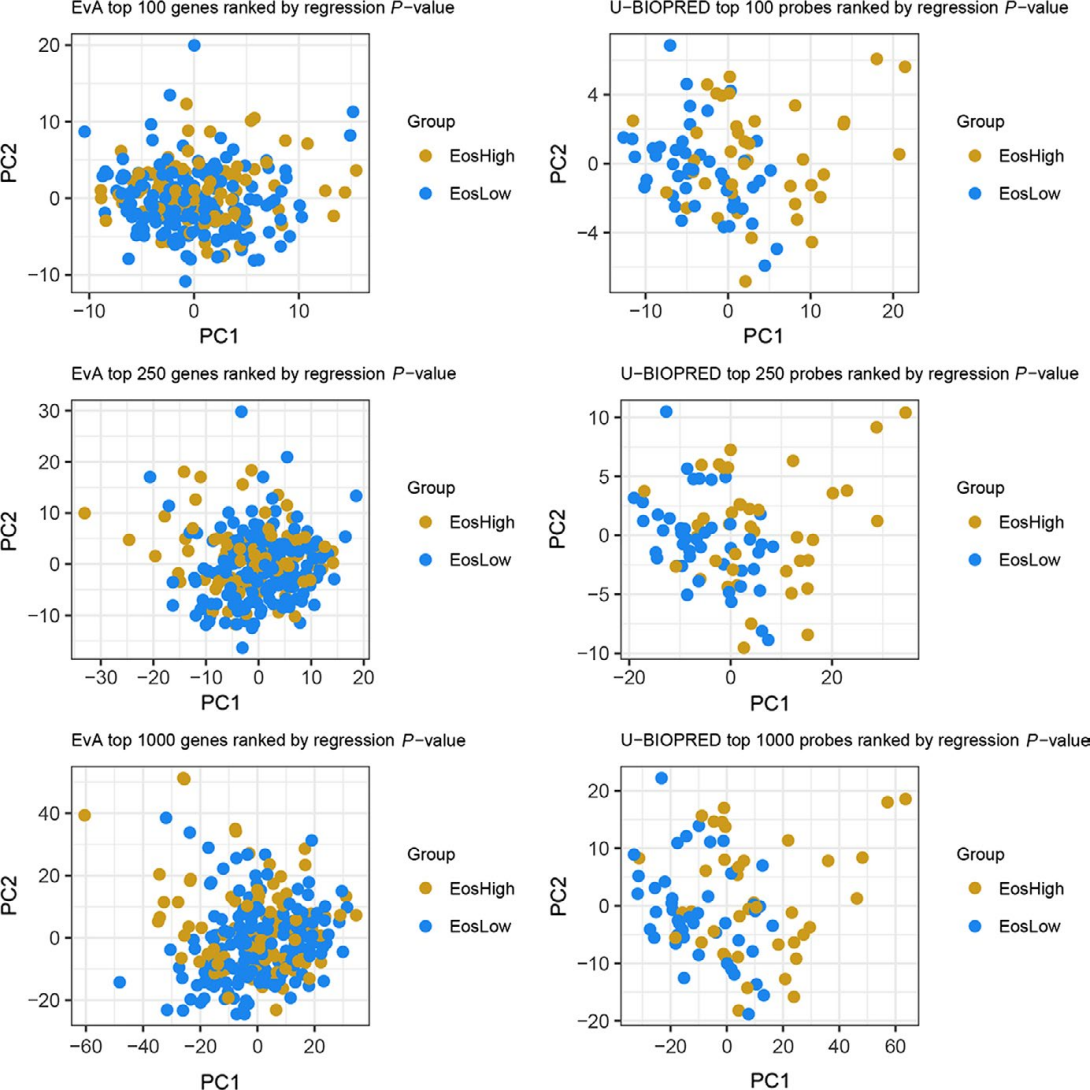
Principal component analysis plots derived from the top 100, 250 and 1000 genes determined by the regression analysis from the COPD (EvA) and asthma (U‐BIOPRED) subjects. Blue dots and red dots represent individual subjects dichotomized by the blood eosinophil count (>200 eosinophils/μL)

Regression analysis of gene expression with linear blood eosinophil counts using a 1% FDR showed that 12 genes were significantly associated (all in positive direction) with the EvA cohort and 1197 (266 in positive and 931 in negative direction) in the U‐BIOPRED cohort. The top 10 up‐ and downregulated genes with respect to linearly increasing levels of blood eosinophils for the EvA and U‐BIOPRED cohorts are shown in Tables 4a,b, and 5a,b, respectively. The 1197 genes in the U‐BIOPRED cohort that met FDR criteria are shown in Table S5. The principal component analysis using the top 100, 250 and 1000 genes with most significant regression coefficients from the EvA and U‐BIOPRED cohorts showed that subjects with and without a blood eosinophil count > 200 cells/μL could not be distinguished in the EvA COPD cohort but showed separation in the U‐BIOPRED asthma cohort (Figure 1). Adjusting the regression models for gender and centre, sensitivity analyses suggested that nine genes (*CST1, CLCA1, FETUB, CAPN14, CPA4, C5orf17, CCL26, RAET1L* and *SLC24A3*), of which seven were common with the uncorrected analyses, were related to the blood eosinophil count in COPD and 941 genes, all of which were contained in the unadjusted analysis, in asthma.

**Table 4:** Regression analysis of blood eosinophils and gene expression. Top ten (a) upregulated (b) downregulated genes in EvA COPD

Gene symbol	Regression coefficient	Average gene expression (log2)	*P* value	*P* value FDR corrected
(a)				
*CST1*	3.78	−1.03	2.8E‐19	2.8E‐15
*CLCA1*	3.73	−2.35	1.8E‐26	3.6E‐22
*FETUB*	2.67	−2.11	3.8E‐14	2.6E‐10
*CPA4*	2.39	−0.78	7.1E‐10	2.9E‐06
*KLK7*	2.20	−2.11	5.2E‐07	1.3E‐03
*SPRR3*	2.18	1.69	2.0E‐06	4.0E‐3
*CAPN14*	2.12	−1.88	5.3E‐10	2.7E‐06
*C5orf17*	2.11	−3.89	3.6E‐06	6.7E‐3
*AC019349.5*	2.03	−0.60	7.3E‐07	1.6E‐03
*CCL26*	1.99	−2.84	2.2E‐09	7.2E‐06
(b)				
*RP11‐627G23.1*	−2.42	0.33	3.0E‐04	0.08
*RP11‐532E4.2*	−1.88	−0.38	2.0E‐04	0.06
*MUC5B*	−1.30	8.63	2.0E‐04	0.06
*C3*	−1.08	9.05	4.0E‐04	0.08
*TMEM45A*	−1.05	5.75	4.0E‐04	0.09
*PLK3*	−0.78	2.59	4.0E‐04	0.08
*INPP5J*	−0.75	1.48	4.0E‐04	0.08
*SPAG17*	−0.69	6.49	2.0E‐04	0.06
*SLC34A2*	−0.57	8.92	5.0E‐04	0.09
*PDE4DIP*	−0.37	6.53	4.0E‐04	0.09

Abbreviations: C3, complement C3; C5orf17, chromosome 5 open reading frame 17; CAPN14, calpain 14; CCL26, C‐C motif chemokine ligand 26; CLCA1, calcium‐activated chloride channel protein 1; CPA4, carboxypeptidase A4; CST1, cystatin SN; FETUB, fetuin‐B; INPP5J, inositol polyphosphate‐5‐phosphatase J; KLK7, kallikrein‐related peptidase 7; MUC5B, mucin 5B; PDE4DIP, phosphodiesterase 4D interacting protein; PLK3, serine/threonine‐protein kinase PLK3; SPAG17, sperm‐associated antigen 17SLC34A2, solute carrier family 34 member 2; SPRR3, small proline‐rich protein 3; TMEM45A, transmembrane protein 45A.

**Table 5:** Regression analysis of blood eosinophils and gene expression. Top ten (a) upregulated (b) downregulatedgenes in U‐BIOPRED asthma

Gene symbol	Regression coefficient	Average intensity	*P* value	*P* value FDR corrected
(a)				
*CST1*	5.20	5.37	4.0E‐05	2.0E‐03
*SRGN*	3.22	5.52	9.1E‐05	3.0E‐03
*TPSAB1///TPSB2*	3.12	6.14	4.8E‐05	2.0E‐03
*CST4*	3.12	5.32	3.7E‐06	1.0E‐03
*S100A8*	3.05	7.00	3.9E‐04	7.0E‐03
*IGK///IGKC*	2.99	5.81	2.7E‐05	1.0E‐03
*PTPRC*	2.97	5.47	1.2E‐04	3.0E‐03
*ALOX5AP*	2.65	6.22	1.5E‐04	4.0E‐03
*LCP1*	2.65	5.98	1.2E‐04	3.0E‐03
*CXCR4*	2.59	6.89	5.3E‐04	8.0E‐03
(b)				
*MSMB*	−2.90	10.72	1.4E‐06	3.0 E‐04
*MUC5B*	−2.74	10.92	8.8E‐08	1.0E‐04
*MKL2*	−2.50	5.48	6.3E‐06	7.0E‐04
*SCGB3A1*	−2.44	11.5	1.1E‐05	9.0E‐04
*THSD4*	−2.29	5.98	4.4E‐06	6.0E‐04
*SULT1E1*	−2.21	5.44	1.9E‐04	4.4E‐03
*ANKUB1*	−2.20	5.44	3.6E‐04	6.6E‐03
*ADAM12*	−2.19	5.88	7.2E‐06	7.0E‐03
*RIBC1*	−2.08	6.35	2.3E‐05	1.3E‐03
*BMS1P6*	−2.06	5.93	1.2E‐06	3.0E‐04

Abbreviations: ADAM12, ADAM metallopeptidase; ALOX5AP, arachidonate 5‐lipoxygenase‐activating protein; ANKUB1, ankyrin repeat and ubiquitin domain containing 1; BMS1P6, BMS1‐like, ribosome assembly protein pseudogene; CST 1 and 4, cystatin 1 and 4; CXCR4, chemokine (C‐X‐C) motif receptor 4; IGK///IGKC, immunoglobulin kappa locus///immunoglobulin kappa constant; LCP1, lymphocyte cytosolic protein 1; MKL2, MKL1/myocardin‐like 2; MSMB, microseminoprotein beta; MUC5B, mucin 5B oligomeric mucus/gel forming; PTPRC, protein tyrosine phosphatase, receptor type C; RIBC1, RIB43A domain with coiled‐coils 1; S100A8, S100 calcium‐binding protein 8; SCGB3A1, secretoglobin family 3A member 1; SRGN, serglycin; SULT1E1, sulfotransferase family 1E member 1; TBSB2, tryptase beta2; THSD4, thrombospondin, type 1 domain containing 4; TPSAB1, tryptase alpha/beta 1.

The number of genes that coincided between asthma and COPD in the top 100, 250 and 1000 genes (ranked by *P*‐value in the unadjusted regression models) was 1, 2 and 28 respectively. The transcriptome thus showed little overlap between genes associated with linear blood eosinophil counts in asthma versus COPD with *CST1* the only gene associated with eosinophilic asthma and COPD meeting FDR control. The clinical characteristics of the replication cohorts are shown in Table S6. In the replication cohorts, CST1 and the blood eosinophil count were weakly correlated in asthma (n = 213) (*r* = .21; 95% CI 0.08 to 0.36; *P* = .002) and in COPD (n = 79) (*r* = .21; 95% CI −0.01 to 0.41; *P* = .06).

Pathway analysis was undertaken derived from the most significant genes in the regression analyses. There were very few pathways that were common between the EvA and U‐BIOPRED cohorts when top 100, 250 and 1000 genes were used (Figure S1).

## 4 DISCUSSION

We found little difference with respect to clinical data between eosinophilic and noneosinophilic COPD with the only notable difference being that a high blood eosinophil count was related to increased levels of total IgE. The gene expression profiles of bronchial epithelial brush samples from COPD subjects categorized as eosinophilic versus noneosinophilic determined by blood eosinophil count > 200 eosinophils/μL did not point to any genes that were differentially expressed between the two COPD subtypes. In the COPD subjects, only 12 genes were associated with the linear blood eosinophil count. All of these were positive associations, and the lack of negative associations is probably a reflection of the small number of genes that met false discovery rate criteria. In contrast, over 1000 genes were related to the linear blood eosinophil level in asthma. The overlap was minimal between the genes and pathways associated with the blood eosinophil count identified in asthma and COPD with *CST1* the only gene meeting FDR criteria.

We had anticipated that eosinophilic COPD might reveal similar bronchial epithelial T2^HIGH^ gene expression profiles as observed in asthma^17^, ^18^, ^22^, ^23^ and asthma‐COPD overlap syndrome.^20^ For example, previous studies reported 3 key genes *POSTN* (periostin), *CLCA1* (chloride channel accessory 1) and *SERPINB2* (serpin family B member 2), which has been extended to a more comprehensive 100 T2^HIGH^ genes.^20^ Here, we did find that four of the top 10 genes related to a blood eosinophil count were in the 100 T2^HIGH^ genes in COPD *CLCA1, CST1, SPRR3* and *CCL26* and in asthma *CST1, CST4, TPSAB1* and *IGK/IGKC*. This suggests that in eosinophilic COPD although we were unable to demonstrate distinct gene clusters, genes previously associated with T2‐mediated immunity were indeed increased. In further support of T2‐immunity playing a role in eosinophilic COPD, total IgE was associated with blood eosinophils suggesting a possible role for allergic sensitization although atopy is consistently not reported to be increased in this group. However, critically the number of genes that were related to a blood eosinophil count in COPD was very few compared to asthma suggesting that T2‐immunity is unlikely to play a major role in COPD compared to asthma and might reflect different mechanisms driving eosinophilic inflammation in COPD. This underscores the complexity and heterogeneity of the airway inflammation in COPD and asthma^3^, ^31^ and highlights the challenges in identifying common molecular signatures. Indeed, confounders could include smoking status, atopy, disease severity, treatment and cellular composition.

Notwithstanding our major finding that in asthma and COPD, there were very few common genes and pathways related to a blood eosinophil count and there was one notable exception. In both asthma and COPD, *CST1* was the gene most positively related to a blood eosinophil count and indeed was the only gene that met FDR criteria in both asthma and COPD. CST1 was weakly correlated to the blood eosinophil count in our replication asthma and COPD cohorts. However, this correlation was significant for asthma but did not meet significance in COPD possibly due to a smaller sample size (n = 79 in the COPD replication cohort). *CST1* is the gene for cystatin SN. Cystatin SN is a cysteine protease inhibitor expressed by the airway epithelium and is implicated in T2‐mediated innate immunity and epithelial repair.^24^, ^25^ Its expression by epithelial cells is upregulated by TSLP and IL‐33, and it reciprocally amplifies the release of these “alarmins”.^25^ Additionally, cystatin SN directly stimulates fibroblasts to release eosinophil‐directed chemokines.^25^ Thus, cystatin SN can promote eosinophilic inflammation via activation of innate lymphoid cells or through recruitment via mesenchymal cell release of CCR3 chemokines. *MUC5B* was in the top 10 genes most negatively related to a blood eosinophil count in both asthma and COPD, but did not meet FDR criteria in the COPD group. The MUC5AC:MUC5B ratio is increased in eosinophilic asthma^26^ consistent with the negative association between MUC5B expression and a blood eosinophil count. MUC5AC is expressed by goblet cells within the epithelium, whereas MUC5B is expressed predominately in the mucus glands.^27^ Thus, whether the negative relationship between MUC5B expression in the bronchial brushes and a blood eosinophil count reflects differences in site and number of mucus glands warrants further investigation.

Increased eosinophilic inflammation in peripheral blood and sputum samples in asthma and COPD is associated with favourable responses to corticosteroids^1^, ^2^, ^3^, ^28^, ^29^ and is associated with increased risk of exacerbations following corticosteroid withdrawal.^30^ These findings suggest that a high blood eosinophil count represents a common “treatable trait” shared between asthma and COPD. Anti‐IL5 biological treatment is consistently beneficial in asthma^31^ and is now a licensed therapy. However, the response to anti‐IL5 and IL‐5R monoclonal antibodies in COPD has been disappointing with benefits related to the intensity of the blood eosinophil count as seen in asthma but the magnitude of response greatly reduced.^2^, ^32^, ^33^, ^34^ Thus, both the epithelial gene expression profile and response to T2‐directed biological therapies differ between eosinophilic asthma and COPD.

Our study has a number of limitations. Although the sample size is relatively large, we might have failed to observe important small differences between groups. Interestingly, more genes were statistically associated with a blood eosinophil count in the asthma group suggesting our findings were not simply due to differences in sample size. Our study was cross‐sectional, and the stability of the eosinophilic phenotype and the associated gene expression profile cannot be determined in this study. Although previous reports suggest the stability of the eosinophilic phenotype is moderate‐to‐good suggesting our findings are valid.^7^, ^28^ A number of possible confounders could have influenced our study. We explored the effect of age and gender which were confounders in the COPD and not asthma groups but did not affect the striking differences observed between genes associated with blood eosinophils in asthma versus COPD. We chose both to study the groups with a single blood eosinophil cut‐point to generate dichotomous groups and also to study the relationship between genes and blood eosinophil counts as a continuous distribution. The choice of cut‐off is between the lower threshold that has demonstrated benefit from anti‐IL5 biologics in asthma, that is 150 cells/μL^21^ and current guidelines for directing the use of ICS in COPD 300 cells/μL.^2^ However, the cut‐off we chose is somewhat arbitrary and other cut‐offs could have been selected. The total IgE was higher in the eosinophilic versus noneosinophilic asthma and COPD groups, but specific IgE was not assessed in the COPD study to explore the link with atopy further. We have compared RNAseq from the EvA cohort and Affymetrix profiles from the U‐BIOPRED cohort. Both of these techniques are widely used, and in a study in activated T cells, there was a high correlation between gene expression profiles generated by the two platforms^35^ suggesting the differences we observed are unlikely to be explained by different technologies although we cannot exclude this possibility.

In conclusion, we found very few differentially expressed genes in bronchial epithelial brushes from COPD in contrast to many in asthma that were related to the blood eosinophil count. Some, but not all, of these genes were consistent with previously described T2^HIGH^ gene profiles. The gene expression profiles between the high and low eosinophil groups of COPD and asthma were broadly different with the exception of one gene *CST1* that was the gene most positively related to a blood eosinophil count in both diseases. Given the recent interest in the concept of “treatable traits”, the finding that the same clinical trait, namely the blood eosinophil count, was apparently supported by predominately differing biology in these two diseases suggests this shared trait has different underlying mechanisms in the lung.

## CONFLICT OF INTEREST

The authors acknowledge the contributions from the wider EvA and UBIOPRED consortia.

## AUTHOR CONTRIBUTIONS

LG, ART and AEC contributed equally to data analysis, data interpretation and writing of the manuscript. MA, GT, SH, IG, MT and LE oversaw the data analysis, data interpretation and writing of the manuscript. MB, PH, WT, TH, SW, SS and MR undertook the analysis in the replication cohorts and contributed to data interpretation and writing of the manuscript. SB, SP, SW, AB, AP, PB, DP, AN, IB, JH, TG, PV, RD, CA, BD, SE, IA and KFC contributed to the data collection, data analysis, data interpretation and writing of the manuscript. CEB, LZH, DS, PS and JH designed the study contributed to data collection, data analysis, data interpretation and writing of the manuscript. All authors reviewed and approved the manuscript.
