# Supplementary material for: Blood eosinophil count and airway epithelial transcriptome relationships in COPD versus asthma
Source: Allergy. 2019 Sep 10;75(2):370–80. doi: 10.1111/all.14016 (PMC7064968; doi:10.1111/all.14016)

**Supplemental Figure 1.** The number of pathways implicated from the top 100, 250 and 1000 genes determined by the regression analysis for the COPD (EvA) in orange and asthma (UBIOPRED) in green subjects with overlapping pathways shown in the intersection.


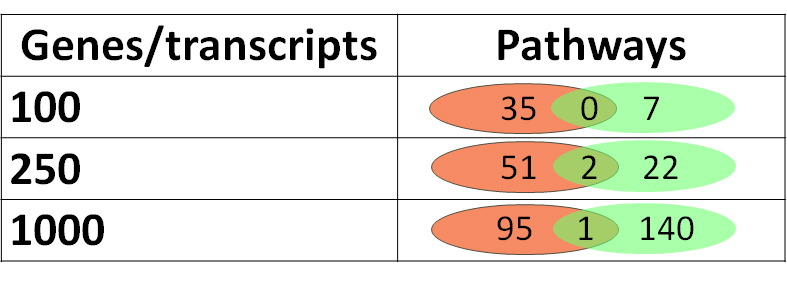

Supplement: Supplementary file 1 [file ALL-75-370-s001.docx]
