# Supplementary material for: Blood eosinophil count and airway epithelial transcriptome relationships in COPD versus asthma
Source: Allergy. 2019 Sep 10;75(2):370–80. doi: 10.1111/all.14016 (PMC7064968; doi:10.1111/all.14016)
Supplement: Supplementary file 2 [file ALL-75-370-s002.docx]

**Table E1. Correlation of COPD clinical characteristics with sputum eosinophils in EvA (n=256)**

|  | Sputum eosinophil  Correlation r | P value | N |
| --- | --- | --- | --- |
| Age (years) | -0.027 | 0.664 | 256 |
| Smoking history (pack years) | 0.041 | 0.518 | 256 |
| BMI kg/m^2^ | 0.015 | 0.808 | 256 |
| 6MWD (m) | 0.111 | 0.081 | 247 |
| BODE index | 0.044 | 0.487 | 256 |
| *Pulmonary Function Tests* |  |  |  |
| FEV_1_ % predicted | -0.130 | **0.038** | 255 |
| FEV_1_/FVC % | -0.130 | **0.038** | 255 |
| Bronchodilator response (%) | 0.042 | 0.500 | 255 |
| RV/TLC % predicted | 0.095 | 0.135 | 249 |
| TLCO/VA % predicted | -0.072 | 0.258 | 248 |
| *CT Parameters* |  |  |  |
| Lung density Perc15 HU | -0.066 | 0.333 | 219 |
| Percentage wall area | 0.120 | 0.068 | 233 |
| *Blood parameters* |  |  |  |
| Blood leucocytes10^9^cells/L | 0.005 | 0.933 | 256 |
| Blood neutrophils 10^9^cells/L | -0.116 | 0.065 | 256 |
| Blood eosinophils 10^9^cells/L | 0.280 | **<0.001** | 256 |
| Blood IgE kU/L ^#^ | 0.043 | 0.495 | 252 |
| *Sputum parameters* |  |  |  |
| Sputum neutrophils (%) ^#^ | -0.209 | **<0.001** | 256 |
| Sputum eosinophils (%) ^#^ | 1.000 |  | 256 |
| Sputum MPO (pg/mL) ^#^ | 0.152 | 0.070 | 143 |
| Sputum HNL (pg/mL) ^#^ | 0.213 | **0.011** | 142 |
| Sputum ECP (pg/mL) ^#^ | 0.428 | **<0.001** | 143 |

Pearson correlation unless stated ^#^Spearman correlation BMI: body mass index; 6MWD: 6-min walk distance; BODE: body mass index, airflow obstruction, dyspnoea, exercise; FEV1: forced expiratory volume in 1s; FVC: forced vital capacity; RV: residual volume; TLC: Total lung capacity; TLCO: transfer capacity of the lungs for carbon monoxide ; VA: alveolar volume; CT: Computed Tomography; Perc15: 15^th^ percentile point; HU: Hounsfield unit; IgE: Immunoglobulin E; MPO: Myeloperoxidase; HNL: Human neutrophil lipocalin; ECP : eosinophil cationic protein

# Table E2. EvA- clinical characteristics of eosinophilic versus non-eosinophilic COPD subjects using blood cell count cut-off >200 eosinophils/μL for those with available bronchial epithelial brush transcriptomic data

|  | Eosinophilic COPD | N | Non-eosinophilic COPD | N | P-value |
| --- | --- | --- | --- | --- | --- |
| Gender (male [n]) | 74 | 99 | 111 | 184 | 0.065 |
| Age (years) | 64 (1) | 99 | 65 (1) | 184 | 0.185 |
| Smoking history (pack years) | 39 (2) | 99 | 39 (1) | 184 | 0.968 |
| BMI kg/m^2^ | 29 (0.5) | 99 | 28 (0) | 184 | 0.051 |
| 6MWD (m) | 455 (12) | 96 | 457 (8) | 184 | 0.895 |
| BODE index | 2 (0) | 99 | 2 (0) | 184 | 0.114 |
| *Pulmonary Function Tests* |  |  |  |  |  |
| FEV_1_ % predicted | 72 (1) | 99 | 74 (1) | 184 | 0.316 |
| FEV_1_/FVC % | 57 (1) | 99 | 57 (1) | 184 | 0.465 |
| Bronchodilator response (%) | 9 (1) | 99 | 9 (1) | 184 | 0.414 |
| RV/TLC % predicted | 1.22 (0.02) | 98 | 1.24 (0.02) | 172 | 0.597 |
| TLCO/VA % predicted | 82 (3) | 97 | 85 (2) | 172 | 0.259 |
| *CT Parameters* |  |  |  |  |  |
| Lung density Perc15 HU | -919 (2) | 89 | -918 (2) | 148 | 0.750 |
| Percentage wall area | 65 (1) | 87 | 64 (1) | 158 | 0.409 |
| *Blood parameters* |  |  |  |  |  |
| Blood leucocytes10^9^cells/L | 7.72 (0.17) | 99 | 7.06 (0.13) | 184 | **0.003** |
| Blood neutrophils 10^9^cells/L | 4.87 (0.15) | 99 | 4.5 (0.11) | 184 | **0.038** |
| Blood eosinophils 10^9^cells/L | 0.357 (0.028) | 99 | 0.11 (0.003) | 184 | **<0.001** |
| Blood IgE kU/L ^#^ | 77 (28-174) | 98 | 32 (13-81) | 182 | **<0.001** |
| *Sputum parameters* |  |  |  |  |  |
| Sputum neutrophils (%) ^#^ | 74 (56-89) | 58 | 78 (68-87) | 98 | 0.448 |
| Sputum eosinophils (%) ^#^ | 3 (0-7) | 58 | 1 (0-4) | 98 | **0.049** |
| Sputum MPO (pg/mL) ^#^ | 261 (130-524) | 41 | 409 (230-629) | 70 | 0.079 |
| Sputum HNL (pg/mL) ^#^ | 1430 (469-1877) | 41 | 1677 (911-2961) | 69 | 0.136 |
| Sputum ECP (pg/mL) ^#^ | 224 (59-528) | 41 | 164 (67-572) | 70 | 0.795 |

Mean (SEM) unless otherwise stated; ^#^ median (interquartile range); BMI: body mass index; 6MWD: 6-min walk distance; BODE: body mass index, airflow obstruction, dyspnoea, exercise; FEV1: forced expiratory volume in 1s; FVC: forced vital capacity; RV: residual volume; TLC: Total lung capacity; TLCO: transfer capacity of the lungs for carbon monoxide ; VA: alveolar volume; CT: Computed Tomography; Perc15: 15^th^ percentile point; HU: Hounsfield unit; IgE: Immunoglobulin E; MPO: Myeloperoxidase; HNL: Human neutrophil lipocalin; ECP : eosinophil cationic protein

**Table E3. U-BIOPRED- clinical characteristics of eosinophilic versus non-eosinophilic asthma subjects using blood cell count cut-off >200 eosinophils/μL for those with bronchial epithelial brush transcriptome**

|  | **Eosinophilic Asthma** | **Non-eosinophilic Asthma** | **P value** |  |
| --- | --- | --- | --- | --- |
| Number | 40 | 45 |  |  |
| Gender (males) | 20 | 19 | 0.377 |  |
| Age | 44.6 (2.47) | 44.4 (2.12) | 0.928 |  |
| Smoking History (Pack Years) | 2.5 (0.31) | 1.6 (0.23) | 0.348 |  |
| BMI | 27.7 (0.85) | 30 (0.96) | 0.075 |  |
| *Pulmonary Function tests* |  |  |  |  |
| Pre-bronchodilator FEV1 (L) | 2.6 (0.19) | 2.7 (0.1) | 0.806 |  |
| FEV1 % Predicted | 88.5 (3.71) | 92.5 (2.87) | 0.395 |  |
| FEV1 / FVC (%) | 71.5 (1.94) | 74.9 (1.61) | 0.18 |  |
| Bronchodilator response (%) | 14.7 (2.56) | 8.1 (0.91) | **0.018** |  |
| Residual Volume (RV) L | 2.1 (0.13) | 2.0 (0.12) | 0.449 |  |
| Total Lung Capacity (TLC) L | 6.4 (0.23) | 6.0 (0.16) | 0.27 |  |
| Total Lung Capacity % Predicted | 106.9 (1.83) | 105.8 (1.9) | 0.703 |  |
| RV / TLC (%) | 33.9 (1.74) | 32.7 (1.36) | 0.628 |  |
| *Patient Reported Outcomes* |  |  |  |  |
| ACQ1-5 Average | 1.5 (0.14) | 1.4 (0.17) | 0.772 |  |
| AQLQ Environment | 5.2 (0.21) | 5.4 (0.22) | 0.525 |  |
| AQLQ Emotional | 5.6 (0.19) | 5.7 (0.25) | 0.805 |  |
| AQLQ Symptoms | 5.3 (0.17) | 5.4 (0.17) | 0.719 |  |
| AQLQ Activity | 5.3 (0.18) | 5.3 (0.16) | 0.757 |  |
| *Blood Parameters* |  |  |  |  |
| Blood Lymphocytes (x10^9^ cells/L) | 1.9 (0.09) | 1.8 (0.08) | 0.17 |  |
| Blood Neutrophils (x10^9^ cells/L) | 4.3 (0.28) | 4.4 (0.28) | 0.747 |  |
| Blood Eosinophils (x10^9^ cells/L) | 0.4 (0.03) | 0.1 (0.01) | **<0.001** |  |
| Blood IgE (IU/mL) | 477.3 (120.69) | 146.9 (29.77) | **0.011** |  |
| *Sputum Parameters* |  |  |  |  |
| Sputum Eosinophils % ^#^ | 2.4 (0.57, 8.39) | 0.5 (0, 1.88) | 0.978 |  |
| Sputum Neutrophils % ^#^ | 48.4 (32.43, 64.63) | 54.1 (45.08, 74.5) | 0.297 |  |

Mean (SEM) unless otherwise stated; ^#^ median (interquartile range). BMI: body mass index; AQLQ: Asthma Quality of Life Questionnaire; ACQ: Asthma Control Questionnaire; FEV1: forced expiratory volume in 1s; FVC: forced vital capacity; RV: residual volume; TLC: Total lung capacity; IgE: Immunoglobulin E

**Table E4. U-BIOPRED all differentially expressed genes/probesets that met FDR criteria between individuals with high (> 200 eosinophils/μL) and low blood eosinophil counts ranked by expression fold change**

**Table E5. U-BIOPRED all genes/probesets that were significantly correlated with blood eosinophil count after correction for multiplicity**

**Table E6. Clinical characteristics of the asthma and COPD replication cohorts**

|  | Asthma  n=213 | COPD  n=79 |
| --- | --- | --- |
| Gender (male/female) | 80/133 | 66/13 |
| Age (years) | 38 (23) | 61 (8) |
| Smoking history (pack years) | 1 (5) | 44.7 (21.0) |
| FEV_1_ % predicted | 77.0 (33) | 63.3 (8.8) |
| FEV_1_/FVC % | 75 (25) | 50.3 (8.8) |
| Blood eosinophils 10^9^cells/L | 0.20 (0.24) | 0.17 (0.11) |

Mean (SD)
