# Supplementary material for: Blood eosinophil count and airway epithelial transcriptome relationships in COPD versus asthma
Source: Allergy. 2019 Sep 10;75(2):370–80. doi: 10.1111/all.14016 (PMC7064968; doi:10.1111/all.14016)
Supplement: Supplementary file 3 [file ALL-75-370-s003.docx]

**Table E4. U-BIOPRED all differentially expressed genes/probesets that met FDR criteria between individuals with high (> 200 eosinophils/μL) and low blood eosinophil counts ranked by expression fold change**

| **Probe ID** | **Gene Symbol** | **Log FC** | **Average Expression** | **P Value** | **Adjusted P value** |
| --- | --- | --- | --- | --- | --- |
| 214370_PM_at | S100A8 | 1.314 | 7.00 | 0.000257 | 0.0349 |
| 201859_PM_at | SRGN | 1.213 | 6.71 | 0.000517 | 0.0435 |
| 216474_PM_x_at | TPSAB1 /// TPSB2 | 1.168 | 6.14 | 0.000336 | 0.0377 |
| 224795_PM_x_at | IGK /// IGKC | 1.132 | 5.81 | 0.000172 | 0.0340 |
| 207134_PM_x_at | TPSB2 | 1.110 | 5.86 | 0.000182 | 0.0347 |
| 204959_PM_at | MNDA | 1.097 | 5.92 | 0.000466 | 0.0424 |
| 204174_PM_at | ALOX5AP | 1.083 | 6.22 | 0.000228 | 0.0349 |
| 210084_PM_x_at | TPSAB1 | 1.066 | 5.86 | 0.000171 | 0.0340 |
| 208885_PM_at | LCP1 | 1.041 | 5.98 | 0.000331 | 0.0374 |
| 231078_PM_at | SLC25A37 | 1.034 | 6.05 | 0.000792 | 0.0470 |
| 202388_PM_at | RGS2 | 0.992 | 7.08 | 2.36E-05 | 0.0214 |
| 207008_PM_at | CXCR2 | 0.961 | 6.09 | 0.001006 | 0.0492 |
| 216485_PM_s_at | TPSAB1 | 0.954 | 6.21 | 0.000110 | 0.0298 |
| 217023_PM_x_at | TPSAB1 /// TPSB2 | 0.943 | 6.02 | 0.000242 | 0.0349 |
| 203535_PM_at | S100A9 | 0.940 | 6.77 | 0.000134 | 0.0324 |
| 221671_PM_x_at | IGK /// IGKC | 0.924 | 6.09 | 0.000239 | 0.0349 |
| 202497_PM_x_at | SLC2A3 | 0.917 | 5.62 | 6.86E-05 | 0.0268 |
| 214669_PM_x_at | IGKC | 0.911 | 5.75 | 0.000642 | 0.0459 |
| 214677_PM_x_at | IGLC1 | 0.886 | 6.33 | 0.000408 | 0.0407 |
| 221651_PM_x_at | IGK /// IGKC | 0.878 | 5.69 | 0.000937 | 0.0491 |
| 228846_PM_at | MXD1 | 0.860 | 7.63 | 6.35E-05 | 0.0266 |
| 215382_PM_x_at | TPSAB1 | 0.837 | 5.84 | 0.000659 | 0.0460 |
| 226275_PM_at | MXD1 | 0.822 | 6.20 | 0.000110 | 0.0298 |
| 214836_PM_x_at | IGK /// IGKC | 0.806 | 5.93 | 0.000652 | 0.0460 |
| 237137_PM_at | SCARNA2 | 0.798 | 8.03 | 0.000353 | 0.0385 |
| 213603_PM_s_at | RAC2 | 0.796 | 6.14 | 5.24E-05 | 0.0242 |
| 204220_PM_at | GMFG | 0.778 | 5.72 | 5.11E-05 | 0.0242 |
| 215121_PM_x_at | CYAT1 /// IGLC1 /// IGLV1-44 | 0.713 | 5.88 | 0.000634 | 0.0456 |
| 1552703_PM_s_at | CARD16 /// CASP1 | 0.707 | 5.51 | 0.000289 | 0.0363 |
| 209791_PM_at | PADI2 | 0.683 | 5.38 | 0.000226 | 0.0349 |
| 204949_PM_at | ICAM3 | 0.670 | 5.51 | 8.52E-06 | 0.0141 |
| 203508_PM_at | TNFRSF1B | 0.659 | 5.71 | 0.000741 | 0.0470 |
| 1555167_PM_s_at | NAMPT | 0.654 | 6.31 | 0.000483 | 0.0427 |
| 221059_PM_s_at | COTL1 | 0.651 | 6.13 | 9.21E-05 | 0.0298 |
| 215379_PM_x_at | IGLV1-44 | 0.644 | 5.72 | 0.000316 | 0.0372 |
| 1568248_PM_x_at | SNORA71B | 0.625 | 7.18 | 6.62E-05 | 0.0268 |
| 225762_PM_x_at | RNA45S5 | 0.622 | 8.70 | 0.000139 | 0.0324 |
| 201288_PM_at | ARHGDIB | 0.587 | 7.07 | 0.000506 | 0.0429 |
| 203765_PM_at | GCA | 0.582 | 7.86 | 0.000553 | 0.0446 |
| 218136_PM_s_at | SLC25A37 | 0.567 | 6.30 | 0.000921 | 0.0488 |
| 228094_PM_at | AMICA1 | 0.557 | 5.27 | 0.000137 | 0.0324 |
| 205180_PM_s_at | ADAM8 | 0.552 | 5.97 | 0.000190 | 0.0349 |
| 217138_PM_x_at | IGLC1 | 0.533 | 5.61 | 0.000957 | 0.0491 |
| 205922_PM_at | VNN2 | 0.531 | 5.82 | 0.000881 | 0.0481 |
| AFFX-NonspecificGC25_at | NA | 0.502 | 5.36 | 0.000761 | 0.0470 |
| 214508_PM_x_at | CREM | 0.475 | 5.48 | 0.000610 | 0.0456 |
| 224796_PM_at | ASAP1 | 0.472 | 5.68 | 5.78E-05 | 0.0252 |
| 203186_PM_s_at | S100A4 | 0.464 | 7.73 | 0.000313 | 0.0372 |
| 1554481_PM_a_at | EPB41 | 0.460 | 6.18 | 0.000261 | 0.0349 |
| 215946_PM_x_at | IGLL3P | 0.450 | 5.27 | 0.000569 | 0.0448 |
| 239843_PM_at | RIT1 | 0.449 | 6.42 | 0.000347 | 0.0385 |
| 212769_PM_at | TLE3 | 0.447 | 5.37 | 0.000276 | 0.0353 |
| 204319_PM_s_at | RGS10 | 0.422 | 5.92 | 0.000254 | 0.0349 |
| 1557905_PM_s_at | CD44 | 0.413 | 8.83 | 0.000820 | 0.0473 |
| 223649_PM_s_at | SLC25A39 | 0.411 | 5.85 | 0.000145 | 0.0324 |
| 210787_PM_s_at | CAMKK2 | 0.404 | 5.21 | 2.95E-05 | 0.0230 |
| 208785_PM_s_at | MAP1LC3B | 0.398 | 5.81 | 0.000845 | 0.0476 |
| 243099_PM_at | NFAM1 | 0.385 | 5.67 | 0.000105 | 0.0298 |
| 219183_PM_s_at | CYTH4 | 0.371 | 5.35 | 5.04E-05 | 0.0242 |
| 203175_PM_at | RHOG | 0.363 | 5.35 | 0.000760 | 0.0470 |
| 238669_PM_at | PTGS1 | 0.362 | 5.27 | 0.000447 | 0.0424 |
| 1563445_PM_x_at | CTSLP8 | 0.353 | 6.02 | 0.001013 | 0.0493 |
| 203879_PM_at | PIK3CD | 0.317 | 5.87 | 5.35E-05 | 0.0242 |
| 214097_PM_at | RPS21 | 0.312 | 7.10 | 0.001029 | 0.0498 |
| 214534_PM_at | HIST1H1B | 0.310 | 5.15 | 0.000853 | 0.0476 |
| 206128_PM_at | ADRA2C | 0.308 | 5.15 | 0.000230 | 0.0349 |
| 236155_PM_at | ZCCHC6 | 0.306 | 5.42 | 0.000832 | 0.0476 |
| 1565583_PM_at | ZSCAN5A | 0.305 | 5.16 | 0.000781 | 0.0470 |
| 203433_PM_at | MTHFS /// ST20-MTHFS | 0.302 | 5.57 | 0.000331 | 0.0374 |
| 206986_PM_at | FGF18 | 0.300 | 5.29 | 0.000182 | 0.0347 |
| 206687_PM_s_at | PTPN6 | 0.297 | 6.81 | 0.000985 | 0.0491 |
| 237041_PM_x_at | RCOR1 | 0.287 | 5.45 | 0.000214 | 0.0349 |
| 215706_PM_x_at | ZYX | 0.287 | 6.03 | 0.000808 | 0.0470 |
| 201389_PM_at | ITGA5 | 0.264 | 5.24 | 0.000769 | 0.0470 |
| 203777_PM_s_at | RPS6KB2 | 0.254 | 5.83 | 0.000719 | 0.0470 |
| 229165_PM_at | MRPL12 | 0.253 | 5.35 | 0.000825 | 0.0474 |
| 223147_PM_s_at | SFT2D3 /// WDR33 | 0.249 | 5.78 | 0.000507 | 0.0429 |
| 229826_PM_at | SMIM4 | 0.245 | 5.82 | 0.000869 | 0.0478 |
| 209413_PM_at | B4GALT2 | 0.236 | 5.14 | 0.000898 | 0.0482 |
| 219966_PM_x_at | BANP | 0.227 | 5.99 | 0.000730 | 0.0470 |
| 205983_PM_at | DPEP1 | 0.225 | 5.11 | 0.000136 | 0.0324 |
| 221769_PM_at | SPSB3 | 0.221 | 5.52 | 0.000205 | 0.0349 |
| 244191_PM_at | RPLP1 | 0.217 | 5.15 | 0.000773 | 0.0470 |
| 1557120_PM_at | EEF1A1 | 0.210 | 5.67 | 0.000659 | 0.0460 |
| 209367_PM_at | STXBP2 | 0.209 | 6.71 | 0.000104 | 0.0298 |
| 210457_PM_x_at | HMGA1 | 0.207 | 6.34 | 0.000205 | 0.0349 |
| 1560439_PM_at | LINGO3 | 0.159 | 5.85 | 0.000426 | 0.0414 |
| 200058_PM_s_at | LOC101929240 /// SNRNP200 | -0.197 | 7.44 | 0.000709 | 0.0470 |
| 226465_PM_s_at | SON | -0.199 | 9.61 | 0.000855 | 0.0476 |
| 208194_PM_s_at | STAM2 | -0.206 | 8.45 | 0.000925 | 0.0488 |
| 201065_PM_s_at | GTF2I /// GTF2IP1 /// LOC100093631 | -0.209 | 8.93 | 0.000883 | 0.0481 |
| 210047_PM_at | SLC11A2 | -0.211 | 6.03 | 0.000919 | 0.0488 |
| 207169_PM_x_at | DDR1 /// MIR4640 | -0.213 | 8.23 | 0.000596 | 0.0456 |
| 201101_PM_s_at | BCLAF1 | -0.216 | 7.99 | 0.000395 | 0.0404 |
| 1007_PM_s_at | DDR1 /// MIR4640 | -0.216 | 9.14 | 0.000788 | 0.0470 |
| 218449_PM_at | UFSP2 | -0.217 | 6.31 | 0.000469 | 0.0424 |
| 217895_PM_at | PTCD3 | -0.218 | 6.05 | 0.000221 | 0.0349 |
| 235281_PM_x_at | AHNAK | -0.220 | 9.59 | 0.000456 | 0.0424 |
| 1557227_PM_s_at | TPR | -0.223 | 8.11 | 0.000541 | 0.0443 |
| 217877_PM_s_at | GPBP1L1 | -0.224 | 7.45 | 2.95E-05 | 0.0230 |
| 209186_PM_at | ATP2A2 | -0.224 | 10.79 | 0.000272 | 0.0353 |
| 211962_PM_s_at | ZFP36L1 | -0.224 | 9.48 | 0.000384 | 0.0402 |
| 214988_PM_s_at | SON | -0.226 | 7.45 | 9.74E-05 | 0.0298 |
| 201952_PM_at | ALCAM | -0.228 | 9.38 | 0.000469 | 0.0424 |
| 215038_PM_s_at | SETD2 | -0.232 | 5.99 | 0.000464 | 0.0424 |
| 201736_PM_s_at | Mar-06 | -0.234 | 8.49 | 0.000774 | 0.0470 |
| 203896_PM_s_at | PLCB4 | -0.237 | 5.83 | 0.001025 | 0.0497 |
| 208073_PM_x_at | TTC3 /// TTC3P1 | -0.240 | 7.42 | 0.000687 | 0.0470 |
| 203513_PM_at | SPG11 | -0.241 | 6.49 | 0.000797 | 0.0470 |
| 216221_PM_s_at | PUM2 | -0.242 | 6.81 | 0.000464 | 0.0424 |
| 218396_PM_at | VPS13C | -0.245 | 7.77 | 0.000608 | 0.0456 |
| 224818_PM_at | SORT1 | -0.249 | 7.08 | 0.000323 | 0.0374 |
| 212068_PM_s_at | PRRC2B | -0.249 | 6.87 | 0.000612 | 0.0456 |
| 205562_PM_at | RPP38 | -0.253 | 7.11 | 0.000317 | 0.0372 |
| 203375_PM_s_at | TPP2 | -0.257 | 6.41 | 0.000557 | 0.0447 |
| 219121_PM_s_at | ESRP1 | -0.259 | 7.58 | 0.000804 | 0.0470 |
| 208621_PM_s_at | EZR | -0.260 | 9.04 | 0.000179 | 0.0347 |
| 214937_PM_x_at | PCM1 | -0.262 | 8.18 | 0.000171 | 0.0340 |
| 35148_PM_at | TJP3 | -0.263 | 8.13 | 0.001011 | 0.0493 |
| 201561_PM_s_at | CLSTN1 | -0.265 | 8.30 | 0.000565 | 0.0448 |
| 201734_PM_at | CLCN3 | -0.266 | 7.21 | 0.000852 | 0.0476 |
| 228926_PM_s_at | SMARCA2 | -0.268 | 6.94 | 0.000466 | 0.0424 |
| 244768_PM_at | DYNC1H1 | -0.269 | 6.54 | 0.000972 | 0.0491 |
| 200672_PM_x_at | SPTBN1 | -0.271 | 8.23 | 0.000709 | 0.0470 |
| 204744_PM_s_at | IARS | -0.272 | 6.37 | 0.000761 | 0.0470 |
| 208719_PM_s_at | DDX17 | -0.273 | 8.83 | 0.000960 | 0.0491 |
| 204656_PM_at | SHB | -0.273 | 6.27 | 0.000762 | 0.0470 |
| 208023_PM_at | TNFRSF4 | -0.274 | 6.04 | 0.000707 | 0.0470 |
| 206323_PM_x_at | OPHN1 | -0.274 | 11.72 | 0.000303 | 0.0369 |
| 212693_PM_at | MDN1 | -0.275 | 5.79 | 0.000798 | 0.0470 |
| 201951_PM_at | ALCAM | -0.275 | 8.49 | 0.000192 | 0.0349 |
| 217234_PM_s_at | EZR | -0.277 | 10.22 | 5.27E-05 | 0.0242 |
| 228351_PM_at | HEATR1 | -0.279 | 7.44 | 0.000773 | 0.0470 |
| 217230_PM_at | EZR | -0.280 | 9.37 | 0.000145 | 0.0324 |
| 219163_PM_at | ZNF562 | -0.282 | 6.57 | 0.000698 | 0.0470 |
| 208775_PM_at | XPO1 | -0.284 | 7.41 | 0.000109 | 0.0298 |
| 200602_PM_at | APP | -0.285 | 9.21 | 0.000223 | 0.0349 |
| 227234_PM_at | IPO5P1 | -0.290 | 5.40 | 0.000791 | 0.0470 |
| 214118_PM_x_at | PCM1 | -0.293 | 8.15 | 8.27E-06 | 0.0141 |
| 210892_PM_s_at | GTF2I | -0.295 | 8.81 | 0.000105 | 0.0298 |
| 212160_PM_at | XPOT | -0.296 | 5.52 | 0.000652 | 0.0460 |
| 224970_PM_at | NFIA | -0.298 | 7.55 | 0.000944 | 0.0491 |
| 201056_PM_at | GOLGB1 | -0.299 | 6.07 | 0.000866 | 0.0478 |
| 208151_PM_x_at | DDX17 | -0.301 | 8.87 | 0.000196 | 0.0349 |
| 225724_PM_at | FLJ31306 | -0.304 | 6.32 | 0.000882 | 0.0481 |
| 226520_PM_at | LCOR | -0.306 | 6.76 | 2.95E-05 | 0.0230 |
| 212216_PM_at | PREPL | -0.306 | 5.83 | 0.000719 | 0.0470 |
| 239728_PM_at | NA | -0.309 | 6.14 | 0.000662 | 0.0460 |
| 236254_PM_at | VPS13B | -0.310 | 5.83 | 0.000566 | 0.0448 |
| 203569_PM_s_at | OFD1 | -0.311 | 7.64 | 0.000773 | 0.0470 |
| 218827_PM_s_at | CEP192 | -0.311 | 5.36 | 0.000625 | 0.0456 |
| 235260_PM_s_at | PACRGL | -0.313 | 6.54 | 0.000723 | 0.0470 |
| 210912_PM_x_at | GSTM4 | -0.314 | 5.17 | 0.000531 | 0.0442 |
| 208661_PM_s_at | TTC3 /// TTC3P1 | -0.314 | 6.84 | 0.000295 | 0.0364 |
| 221625_PM_at | NA | -0.315 | 6.46 | 0.000354 | 0.0385 |
| 216060_PM_s_at | DAAM1 | -0.318 | 5.86 | 5.02E-05 | 0.0242 |
| 214552_PM_s_at | RABEP1 | -0.318 | 5.67 | 1.84E-05 | 0.0211 |
| 225078_PM_at | EMP2 | -0.319 | 8.46 | 4.16E-05 | 0.0242 |
| 209119_PM_x_at | NR2F2 | -0.319 | 7.94 | 0.000925 | 0.0488 |
| 227802_PM_at | RUFY3 | -0.319 | 6.53 | 0.000850 | 0.0476 |
| 1553295_PM_at | ABCA13 | -0.321 | 7.86 | 0.000729 | 0.0470 |
| 207564_PM_x_at | OGT | -0.321 | 8.43 | 0.000483 | 0.0427 |
| 208718_PM_at | DDX17 | -0.321 | 9.32 | 9.93E-05 | 0.0298 |
| 225221_PM_at | ZKSCAN1 | -0.322 | 8.35 | 0.000328 | 0.0374 |
| 214744_PM_s_at | RPL23 /// SNORA21 | -0.322 | 5.81 | 6.62E-06 | 0.0141 |
| 225112_PM_at | ABI2 | -0.322 | 7.62 | 0.000805 | 0.0470 |
| 225617_PM_at | ODF2 | -0.325 | 6.00 | 0.000297 | 0.0364 |
| 1555808_PM_a_at | EXD2 | -0.326 | 5.29 | 0.001004 | 0.0492 |
| 203987_PM_at | FZD6 | -0.327 | 5.93 | 0.000391 | 0.0402 |
| 214092_PM_x_at | SUGP2 | -0.327 | 6.72 | 0.000274 | 0.0353 |
| 200843_PM_s_at | EPRS | -0.327 | 6.37 | 0.000154 | 0.0327 |
| 203255_PM_at | FBXO11 | -0.329 | 6.55 | 0.000501 | 0.0429 |
| 229665_PM_at | CSTF3 | -0.329 | 6.28 | 0.000627 | 0.0456 |
| 201476_PM_s_at | RRM1 | -0.330 | 5.66 | 0.000271 | 0.0353 |
| 210415_PM_s_at | ODF2 | -0.331 | 6.04 | 4.50E-05 | 0.0242 |
| 225093_PM_at | UTRN | -0.331 | 8.23 | 5.31E-05 | 0.0242 |
| 202622_PM_s_at | ATXN2 | -0.332 | 6.53 | 2.37E-05 | 0.0214 |
| 242696_PM_at | NA | -0.332 | 5.73 | 0.000790 | 0.0470 |
| 212404_PM_s_at | UBE3B | -0.332 | 5.85 | 0.000198 | 0.0349 |
| 41660_PM_at | CELSR1 | -0.334 | 7.94 | 0.000292 | 0.0363 |
| 212307_PM_s_at | OGT | -0.335 | 6.75 | 0.000504 | 0.0429 |
| 221683_PM_s_at | CEP290 | -0.336 | 6.32 | 0.000733 | 0.0470 |
| 209442_PM_x_at | ANK3 | -0.337 | 7.70 | 0.000885 | 0.0481 |
| 207495_PM_at | RAB28 | -0.337 | 5.52 | 0.000620 | 0.0456 |
| 210962_PM_s_at | AKAP9 | -0.337 | 7.19 | 0.000189 | 0.0349 |
| 202220_PM_at | KIAA0907 | -0.338 | 7.49 | 0.000261 | 0.0349 |
| 202911_PM_at | MSH6 | -0.338 | 6.24 | 0.000463 | 0.0424 |
| 201737_PM_s_at | Mar-06 | -0.338 | 6.82 | 0.000326 | 0.0374 |
| 208614_PM_s_at | FLNB | -0.338 | 8.64 | 8.42E-05 | 0.0288 |
| 213328_PM_at | NEK1 | -0.338 | 6.73 | 0.000419 | 0.0414 |
| 228834_PM_at | NA | -0.340 | 8.69 | 0.000458 | 0.0424 |
| 229994_PM_at | NFIA | -0.340 | 7.33 | 0.000233 | 0.0349 |
| 1553162_PM_x_at | PROSER3 | -0.343 | 6.91 | 4.57E-05 | 0.0242 |
| 200606_PM_at | DSP | -0.343 | 8.48 | 0.000309 | 0.0371 |
| 232244_PM_at | KIAA1161 | -0.343 | 5.33 | 5.87E-05 | 0.0252 |
| 212074_PM_at | SUN1 | -0.345 | 7.81 | 0.000425 | 0.0414 |
| 227876_PM_at | ARHGAP39 | -0.348 | 6.39 | 0.000535 | 0.0442 |
| 1557675_PM_at | RAF1 | -0.349 | 5.53 | 0.000493 | 0.0429 |
| 227816_PM_at | NTN1 | -0.351 | 6.53 | 0.000525 | 0.0440 |
| 225964_PM_at | ZXDC | -0.352 | 5.64 | 5.73E-06 | 0.0141 |
| 225465_PM_at | MAGI1 | -0.352 | 6.15 | 4.63E-06 | 0.0141 |
| 225825_PM_at | C20orf194 | -0.355 | 5.87 | 0.000583 | 0.0455 |
| 209487_PM_at | RBPMS | -0.357 | 7.62 | 0.000473 | 0.0425 |
| 226110_PM_at | PTAR1 | -0.357 | 5.82 | 0.000998 | 0.0492 |
| 218554_PM_s_at | ASH1L | -0.358 | 6.30 | 0.000847 | 0.0476 |
| 225127_PM_at | TMEM181 | -0.359 | 5.50 | 0.000777 | 0.0470 |
| 223177_PM_at | NT5DC1 | -0.359 | 6.51 | 0.000167 | 0.0340 |
| 224820_PM_at | COX20 | -0.360 | 6.39 | 0.000130 | 0.0324 |
| 202432_PM_at | PPP3CB | -0.362 | 5.36 | 0.000368 | 0.0390 |
| 228077_PM_at | MRI1 | -0.362 | 6.18 | 0.000255 | 0.0349 |
| 218067_PM_s_at | ARGLU1 | -0.363 | 8.56 | 0.000676 | 0.0466 |
| 237383_PM_at | NA | -0.363 | 5.20 | 0.000145 | 0.0324 |
| 213074_PM_at | PHIP | -0.365 | 7.01 | 0.000851 | 0.0476 |
| 236017_PM_at | CDKL3 | -0.365 | 5.94 | 0.000350 | 0.0385 |
| 227199_PM_at | DIP2A | -0.368 | 5.32 | 0.000277 | 0.0353 |
| 204001_PM_at | SNAPC3 | -0.368 | 6.32 | 0.000370 | 0.0390 |
| 229413_PM_s_at | NA | -0.368 | 5.73 | 0.000984 | 0.0491 |
| 213839_PM_at | CLMN | -0.369 | 7.35 | 0.000596 | 0.0456 |
| 225805_PM_at | HNRNPU | -0.369 | 5.55 | 0.000702 | 0.0470 |
| 231923_PM_at | TMEM150C | -0.370 | 5.97 | 0.000988 | 0.0491 |
| 213998_PM_s_at | DDX17 | -0.371 | 9.66 | 0.000230 | 0.0349 |
| 1557100_PM_s_at | HECTD1 | -0.371 | 7.32 | 0.000120 | 0.0308 |
| 226894_PM_at | SLC35A3 | -0.371 | 6.36 | 0.000400 | 0.0404 |
| 201084_PM_s_at | BCLAF1 | -0.373 | 7.22 | 1.09E-05 | 0.0144 |
| 1553605_PM_a_at | ABCA13 | -0.375 | 7.53 | 0.0006325 | 0.0456 |
| 207902_PM_at | IL5RA | -0.377 | 6.61 | 0.0008573 | 0.0476 |
| 226297_PM_at | HIPK3 | -0.377 | 7.27 | 1.65E-06 | 0.0117 |
| 238844_PM_s_at | NPHP1 | -0.378 | 6.99 | 0.000361 | 0.0388 |
| 227484_PM_at | SRGAP1 | -0.379 | 7.01 | 0.000547 | 0.0443 |
| 1553694_PM_a_at | PIK3C2A | -0.380 | 6.17 | 0.000262 | 0.0349 |
| 1569974_PM_x_at | BC015774 /// SEPT7P2 | -0.381 | 5.28 | 0.000952 | 0.0491 |
| 225444_PM_at | UBN2 | -0.383 | 5.71 | 0.000486 | 0.0427 |
| 213092_PM_x_at | DNAJC9 | -0.383 | 5.80 | 0.000253 | 0.0349 |
| 1558956_PM_s_at | IFT80 | -0.385 | 7.36 | 0.000291 | 0.0363 |
| 217904_PM_s_at | BACE1 | -0.385 | 5.83 | 4.40E-05 | 0.0242 |
| 207766_PM_at | CDKL1 | -0.386 | 6.20 | 0.000241 | 0.0349 |
| 206385_PM_s_at | ANK3 | -0.386 | 8.24 | 0.000154 | 0.0327 |
| 226252_PM_at | ZBTB20 | -0.388 | 6.04 | 0.000251 | 0.0349 |
| 231875_PM_at | KIF21A | -0.389 | 8.96 | 0.000867 | 0.0478 |
| 231909_PM_x_at | ODF2L | -0.389 | 5.41 | 0.000757 | 0.0470 |
| 202126_PM_at | PRPF4B | -0.391 | 6.16 | 3.20E-06 | 0.0138 |
| 226189_PM_at | ITGB8 | -0.392 | 5.50 | 0.000116 | 0.0303 |
| 214608_PM_s_at | EYA1 | -0.393 | 6.00 | 0.000267 | 0.0352 |
| 213792_PM_s_at | INSR | -0.397 | 8.51 | 0.000648 | 0.0460 |
| 244881_PM_at | LMLN | -0.397 | 6.57 | 0.000109 | 0.0298 |
| 227432_PM_s_at | NA | -0.398 | 9.01 | 0.000389 | 0.0402 |
| 223007_PM_s_at | TMEM245 | -0.400 | 5.67 | 0.000992 | 0.0491 |
| 231084_PM_at | WDR96 | -0.401 | 8.89 | 0.000889 | 0.0481 |
| 242647_PM_at | USP34 | -0.401 | 6.32 | 0.000483 | 0.0427 |
| 219511_PM_s_at | SNCAIP | -0.401 | 5.69 | 0.000950 | 0.0491 |
| 212602_PM_at | WDFY3 | -0.402 | 6.20 | 0.000191 | 0.0349 |
| 1565436_PM_s_at | KMT2A | -0.403 | 6.94 | 0.000495 | 0.0429 |
| 218374_PM_s_at | C12orf4 | -0.404 | 5.32 | 0.000967 | 0.0491 |
| 203651_PM_at | ZFYVE16 | -0.405 | 6.01 | 0.000607 | 0.0456 |
| 212079_PM_s_at | KMT2A | -0.406 | 7.25 | 0.000463 | 0.0424 |
| 214889_PM_at | FAM149A | -0.408 | 6.61 | 0.000843 | 0.0476 |
| 212176_PM_at | PNISR | -0.409 | 7.07 | 0.000608 | 0.0456 |
| 232139_PM_s_at | KIAA1919 | -0.411 | 5.99 | 0.000702 | 0.0470 |
| 223696_PM_at | ARSD | -0.412 | 7.54 | 0.000807 | 0.0470 |
| 212362_PM_at | ATP2A2 | -0.413 | 6.74 | 1.59E-05 | 0.0195 |
| 229663_PM_at | LOC100507577 /// LONP2 | -0.414 | 5.34 | 3.81E-05 | 0.0242 |
| 202551_PM_s_at | CRIM1 /// LOC101929500 | -0.414 | 6.17 | 0.000593 | 0.0456 |
| 234006_PM_s_at | TMEM234 | -0.415 | 5.96 | 0.000140 | 0.0324 |
| 243539_PM_at | KIAA1841 | -0.415 | 7.12 | 0.000895 | 0.0482 |
| 239834_PM_at | NA | -0.416 | 5.34 | 0.000818 | 0.0473 |
| 202053_PM_s_at | ALDH3A2 | -0.417 | 7.08 | 0.000258 | 0.0349 |
| 208518_PM_s_at | PER2 | -0.418 | 6.83 | 4.85E-05 | 0.0242 |
| 210111_PM_s_at | KLHDC10 | -0.421 | 5.84 | 0.000398 | 0.0404 |
| 223838_PM_at | TSGA10 | -0.421 | 6.71 | 0.000999 | 0.0492 |
| 226098_PM_at | IFT80 | -0.421 | 6.68 | 7.78E-05 | 0.0279 |
| 203426_PM_s_at | IGFBP5 | -0.425 | 7.61 | 0.000778 | 0.0470 |
| 219886_PM_at | CEP97 | -0.426 | 6.89 | 0.000974 | 0.0491 |
| 205408_PM_at | MLLT10 | -0.427 | 6.28 | 0.000113 | 0.0298 |
| 213649_PM_at | SRSF7 | -0.428 | 6.52 | 0.000891 | 0.0481 |
| 225178_PM_at | TTC14 | -0.430 | 6.69 | 0.000971 | 0.0491 |
| 203494_PM_s_at | CEP57 | -0.430 | 6.25 | 0.000497 | 0.0429 |
| 238431_PM_at | NA | -0.434 | 5.58 | 0.000228 | 0.0349 |
| 239785_PM_at | DZIP1L | -0.434 | 7.37 | 0.000676 | 0.0466 |
| 235512_PM_at | CDKL1 | -0.435 | 6.40 | 0.000949 | 0.0491 |
| 212454_PM_x_at | HNRNPDL | -0.435 | 6.07 | 0.000148 | 0.0327 |
| 234991_PM_at | ZXDC | -0.436 | 5.84 | 0.000423 | 0.0414 |
| 51228_PM_at | RBM12B | -0.439 | 5.51 | 8.72E-05 | 0.0288 |
| 214073_PM_at | CTTN | -0.440 | 5.80 | 0.000422 | 0.0414 |
| 231806_PM_s_at | STK36 | -0.441 | 5.38 | 0.000458 | 0.0424 |
| 234681_PM_s_at | CHD6 | -0.441 | 5.81 | 0.000365 | 0.0389 |
| 228111_PM_s_at | DNAH1 | -0.446 | 7.92 | 0.000240 | 0.0349 |
| 218424_PM_s_at | STEAP3 | -0.448 | 6.07 | 0.000402 | 0.0404 |
| 226404_PM_at | RBM39 | -0.450 | 6.45 | 0.000763 | 0.0470 |
| 236173_PM_s_at | LRIG1 | -0.452 | 6.76 | 9.70E-06 | 0.0141 |
| 218510_PM_x_at | FAM134B | -0.455 | 5.86 | 7.53E-05 | 0.0279 |
| 220952_PM_s_at | PLEKHA5 | -0.455 | 6.91 | 0.000111 | 0.0298 |
| 230403_PM_at | RFX3 | -0.455 | 8.22 | 0.000618 | 0.0456 |
| 236488_PM_s_at | CTD-3092A11.2 | -0.455 | 5.79 | 0.000938 | 0.0491 |
| 211596_PM_s_at | LRIG1 | -0.462 | 7.80 | 8.52E-05 | 0.0288 |
| 228421_PM_s_at | EFEMP1 | -0.463 | 7.76 | 7.79E-05 | 0.0279 |
| 209170_PM_s_at | GPM6B | -0.464 | 6.10 | 0.000981 | 0.0491 |
| 238339_PM_x_at | LRIG1 | -0.465 | 7.28 | 3.62E-05 | 0.0242 |
| 1560910_PM_at | PPIL6 | -0.471 | 5.67 | 5.30E-05 | 0.0242 |
| 230142_PM_s_at | CIRBP | -0.471 | 5.50 | 0.000245 | 0.0349 |
| 226877_PM_at | RPL32P3 | -0.472 | 5.67 | 0.000360 | 0.0388 |
| 218926_PM_at | MYNN | -0.473 | 5.34 | 3.85E-05 | 0.0242 |
| 1563958_PM_at | NA | -0.476 | 5.74 | 0.000635 | 0.0456 |
| 221973_PM_at | LOC100506076 /// LOC100506123 | -0.478 | 6.62 | 0.000487 | 0.0427 |
| 1554512_PM_a_at | CEP89 | -0.480 | 6.21 | 0.000388 | 0.0402 |
| 227809_PM_at | ZC3H6 | -0.480 | 6.06 | 6.13E-06 | 0.0141 |
| 241310_PM_at | NEK5 | -0.480 | 6.88 | 0.000973 | 0.0491 |
| 1568763_PM_s_at | LOC102724884 /// LOC728613 /// PDCD6 | -0.485 | 7.02 | 8.60E-05 | 0.0288 |
| 225180_PM_at | TTC14 | -0.485 | 5.74 | 0.000107 | 0.0298 |
| 215099_PM_s_at | RXRB | -0.486 | 5.58 | 2.04E-06 | 0.0117 |
| 230561_PM_s_at | KANSL1L | -0.488 | 5.29 | 0.000803 | 0.0470 |
| 229325_PM_at | ZZZ3 | -0.492 | 5.40 | 2.26E-05 | 0.0214 |
| 236241_PM_at | MED31 | -0.495 | 5.28 | 0.000142 | 0.0324 |
| 232381_PM_s_at | DNAH5 | -0.500 | 8.50 | 0.000741 | 0.0470 |
| 244331_PM_at | NA | -0.503 | 5.89 | 0.000214 | 0.0349 |
| 1566505_PM_at | ERVK13-1 | -0.507 | 5.72 | 0.000169 | 0.0340 |
| 228030_PM_at | RBM6 | -0.510 | 6.13 | 2.20E-05 | 0.0214 |
| 206318_PM_at | EPPIN /// EPPIN-WFDC6 | -0.510 | 6.15 | 6.74E-05 | 0.0268 |
| 219813_PM_at | LATS1 | -0.511 | 6.03 | 0.000152 | 0.0327 |
| 236000_PM_s_at | NA | -0.512 | 5.80 | 0.000242 | 0.0349 |
| 1557066_PM_at | LUC7L | -0.518 | 5.82 | 0.000657 | 0.0460 |
| 1557636_PM_a_at | C7orf57 | -0.518 | 6.85 | 0.000990 | 0.0491 |
| 234476_PM_at | DNAH7 | -0.519 | 7.26 | 0.000546 | 0.0443 |
| 1552299_PM_at | AK9 | -0.520 | 6.99 | 0.000742 | 0.0470 |
| 226908_PM_at | LRIG3 | -0.520 | 5.84 | 0.000236 | 0.0349 |
| 214745_PM_at | PLCH1 | -0.520 | 5.47 | 0.000344 | 0.0383 |
| 222145_PM_at | NA | -0.531 | 6.22 | 0.000687 | 0.0470 |
| 235511_PM_at | NA | -0.536 | 6.06 | 0.000220 | 0.0349 |
| 242393_PM_x_at | AGAP6 | -0.547 | 7.50 | 0.000619 | 0.0456 |
| 230180_PM_at | DDX17 | -0.548 | 8.27 | 7.18E-05 | 0.0274 |
| 1558075_PM_at | NA | -0.548 | 8.27 | 0.000212 | 0.0349 |
| 237912_PM_at | NA | -0.554 | 7.08 | 0.000318 | 0.0372 |
| 1559410_PM_at | NA | -0.556 | 6.37 | 0.000626 | 0.0456 |
| 225570_PM_at | SLC41A1 | -0.558 | 5.48 | 1.05E-07 | 0.0018 |
| 1569472_PM_s_at | TTC3 /// TTC3P1 | -0.561 | 5.55 | 0.000776 | 0.0470 |
| 233198_PM_at | GOLGA2P5 | -0.563 | 5.96 | 0.000571 | 0.0448 |
| 1559950_PM_at | FAM66B /// FAM66C /// FAM66D /// LOC101928910 /// LOC102725108 | -0.571 | 5.84 | 0.001035 | 0.0499 |
| 1569110_PM_x_at | LOC728613 | -0.578 | 6.98 | 0.000732 | 0.0470 |
| 1561207_PM_at | RP11-53B2.2 | -0.581 | 5.36 | 0.000255 | 0.0349 |
| 228045_PM_at | SUGT1 | -0.585 | 5.55 | 0.000602 | 0.0456 |
| 208498_PM_s_at | ACTG1P4 /// AMY1A /// AMY1B /// AMY1C /// AMY2A /// AMY2B | -0.588 | 7.49 | 0.000631 | 0.0456 |
| 227702_PM_at | CYP4X1 | -0.614 | 6.11 | 0.000914 | 0.0488 |
| 237527_PM_at | NA | -0.615 | 8.08 | 0.000535 | 0.0442 |
| 211959_PM_at | IGFBP5 | -0.632 | 7.18 | 0.000546 | 0.0443 |
| 1557270_PM_at | NA | -0.635 | 5.36 | 0.000572 | 0.0448 |
| 1566502_PM_at | NA | -0.638 | 5.88 | 0.000903 | 0.0483 |
| 231106_PM_at | BMS1P6 | -0.643 | 5.93 | 0.000457 | 0.0424 |
| 234893_PM_s_at | DNAH6 | -0.644 | 5.46 | 0.000203 | 0.0349 |
| 223824_PM_at | RNLS | -0.661 | 6.27 | 0.000992 | 0.0491 |
| 1557866_PM_at | C9orf117 | -0.673 | 7.26 | 0.000157 | 0.0329 |
| 225656_PM_at | EFHC1 | -0.695 | 6.76 | 0.000225 | 0.0349 |
| 1553318_PM_at | RIBC1 | -0.696 | 6.35 | 0.000964 | 0.0491 |
| 213790_PM_at | ADAM12 | -0.703 | 5.88 | 0.000814 | 0.0472 |
| 233312_PM_at | ROPN1L | -0.719 | 5.38 | 0.000305 | 0.0369 |
| 222835_PM_at | THSD4 | -0.722 | 5.98 | 0.000772 | 0.0470 |
| 244635_PM_s_at | NA | -0.761 | 5.95 | 0.000758 | 0.0470 |
| 1555997_PM_s_at | IGFBP5 | -0.891 | 6.24 | 9.82E-06 | 0.0141 |

**Table E5. U-BIOPRED all genes/probesets that were significantly correlated with blood eosinophil count after correction for multiplicity**

| **Probe ID** | **Gene Symbol** | **Log FC** | **Average Expression** | **P Value** | **Adjusted P value** |
| --- | --- | --- | --- | --- | --- |
| 206224_at | CST1 | 5.203 | 5.37 | 0.000040 | 0.00178 |
| 201858_s_at | SRGN | 3.223 | 5.52 | 0.000091 | 0.00286 |
| 201859_at | SRGN | 3.133 | 6.71 | 0.000152 | 0.00386 |
| 216474_x_at | TPSAB1 /// TPSB2 | 3.124 | 6.14 | 0.000048 | 0.00199 |
| 206994_at | CST4 | 3.119 | 5.32 | 0.000004 | 0.00051 |
| 214370_at | S100A8 | 3.046 | 7.00 | 0.000390 | 0.00697 |
| 207134_x_at | TPSB2 | 3.034 | 5.86 | 0.000013 | 0.00102 |
| 224795_x_at | IGK /// IGKC | 2.986 | 5.81 | 0.000027 | 0.00141 |
| 212588_at | PTPRC | 2.973 | 5.47 | 0.000119 | 0.00328 |
| 210084_x_at | TPSAB1 | 2.866 | 5.86 | 0.000018 | 0.00117 |
| 205683_x_at | TPSAB1 | 2.767 | 5.95 | 0.000045 | 0.00190 |
| 216485_s_at | TPSAB1 | 2.668 | 6.21 | 0.000004 | 0.00051 |
| 204174_at | ALOX5AP | 2.654 | 6.22 | 0.000145 | 0.00375 |
| 208885_at | LCP1 | 2.650 | 5.98 | 0.000115 | 0.00323 |
| 217028_at | CXCR4 | 2.594 | 6.89 | 0.000530 | 0.00841 |
| 221651_x_at | IGK /// IGKC | 2.549 | 5.69 | 0.000042 | 0.00180 |
| 221671_x_at | IGK /// IGKC | 2.529 | 6.09 | 0.000019 | 0.00120 |
| 212657_s_at | IL1RN | 2.519 | 5.45 | 0.000264 | 0.00542 |
| 217023_x_at | TPSAB1 /// TPSB2 | 2.468 | 6.02 | 0.000048 | 0.00197 |
| 214836_x_at | IGK /// IGKC | 2.427 | 5.93 | 0.000011 | 0.00092 |
| 207741_x_at | TPSAB1 | 2.409 | 5.96 | 0.000076 | 0.00257 |
| 211924_s_at | PLAUR | 2.368 | 5.71 | 0.000380 | 0.00688 |
| 214181_x_at | LST1 | 2.354 | 5.08 | 0.000003 | 0.00046 |
| 214677_x_at | IGLC1 | 2.353 | 6.33 | 0.000070 | 0.00247 |
| 215382_x_at | TPSAB1 | 2.349 | 5.84 | 0.000047 | 0.00197 |
| 214669_x_at | IGKC | 2.346 | 5.75 | 0.000208 | 0.00467 |
| 209138_x_at | IGLC1 | 2.335 | 5.86 | 0.000060 | 0.00222 |
| 202497_x_at | SLC2A3 | 2.274 | 5.62 | 0.000032 | 0.00158 |
| 216236_s_at | SLC2A14 /// SLC2A3 | 2.209 | 5.20 | 0.000251 | 0.00528 |
| 202388_at | RGS2 | 2.187 | 7.08 | 0.000105 | 0.00306 |
| 213603_s_at | RAC2 | 2.119 | 6.14 | 0.000005 | 0.00060 |
| 203535_at | S100A9 | 2.083 | 6.77 | 0.000414 | 0.00717 |
| 228846_at | MXD1 | 2.024 | 7.63 | 0.000079 | 0.00264 |
| 1568248_x_at | SNORA71B | 2.012 | 7.18 | 0.000000 | 0.00004 |
| 226275_at | MXD1 | 2.000 | 6.20 | 0.000075 | 0.00257 |
| 215121_x_at | CYAT1 /// IGLC1 /// IGLV1-44 | 1.933 | 5.88 | 0.000086 | 0.00277 |
| 225762_x_at | RNA45S5 | 1.906 | 8.70 | 0.000000 | 0.00017 |
| 204220_at | GMFG | 1.898 | 5.72 | 0.000032 | 0.00158 |
| 209791_at | PADI2 | 1.854 | 5.38 | 0.000021 | 0.00124 |
| 204949_at | ICAM3 | 1.840 | 5.51 | 0.000000 | 0.00010 |
| 217148_x_at | IGLC1 /// IGLJ3 /// IGLV2-14 /// IGLV@ | 1.840 | 5.14 | 0.000000 | 0.00004 |
| 208576_s_at | HIST1H3B | 1.822 | 5.25 | 0.000653 | 0.00937 |
| NA | NA | 1.804 | 5.36 | 0.000000 | 0.00008 |
| 209083_at | CORO1A | 1.796 | 5.51 | 0.000021 | 0.00125 |
| 201288_at | ARHGDIB | 1.773 | 7.07 | 0.000007 | 0.00069 |
| 215379_x_at | IGLV1-44 | 1.731 | 5.72 | 0.000040 | 0.00178 |
| 205180_s_at | ADAM8 | 1.672 | 5.97 | 0.000001 | 0.00028 |
| 221059_s_at | COTL1 | 1.672 | 6.13 | 0.000022 | 0.00126 |
| 210597_x_at | PRB1 | 1.626 | 5.95 | 0.000005 | 0.00063 |
| 225799_at | LINC00152 /// LOC101930489 /// MIR4435-1HG | 1.615 | 6.82 | 0.000011 | 0.00092 |
| 215159_s_at | NADK | 1.608 | 5.25 | 0.000001 | 0.00031 |
| 216881_x_at | PRB4 | 1.597 | 5.11 | 0.000030 | 0.00153 |
| 228527_s_at | SLC25A37 | 1.528 | 5.11 | 0.000052 | 0.00206 |
| 202934_at | HK2 | 1.508 | 6.88 | 0.000139 | 0.00365 |
| 203765_at | GCA | 1.502 | 7.86 | 0.000168 | 0.00411 |
| 203186_s_at | S100A4 | 1.499 | 7.73 | 0.000000 | 0.00017 |
| 219161_s_at | CKLF /// CKLF-CMTM1 | 1.480 | 5.79 | 0.000390 | 0.00697 |
| 1555812_a_at | ARHGDIB | 1.475 | 6.87 | 0.000162 | 0.00402 |
| 202665_s_at | WIPF1 | 1.460 | 5.82 | 0.000614 | 0.00913 |
| 201324_at | EMP1 | 1.451 | 5.43 | 0.000539 | 0.00852 |
| 225195_at | DPH3 | 1.450 | 5.09 | 0.000149 | 0.00380 |
| 207752_x_at | PRB1 | 1.444 | 5.97 | 0.000028 | 0.00144 |
| 203523_at | LSP1 | 1.414 | 6.26 | 0.000302 | 0.00590 |
| 204265_s_at | GPSM3 | 1.397 | 5.04 | 0.000099 | 0.00299 |
| 38149_at | ARHGAP25 | 1.392 | 5.19 | 0.000158 | 0.00394 |
| 228094_at | AMICA1 | 1.350 | 5.27 | 0.000101 | 0.00303 |
| 203388_at | ARRB2 | 1.347 | 5.20 | 0.000044 | 0.00186 |
| 200600_at | MSN | 1.330 | 7.34 | 0.000417 | 0.00719 |
| 224796_at | ASAP1 | 1.325 | 5.68 | 0.000001 | 0.00031 |
| 211531_x_at | PRB1 | 1.321 | 6.01 | 0.000121 | 0.00332 |
| 204490_s_at | CD44 | 1.283 | 6.93 | 0.000392 | 0.00699 |
| 215946_x_at | IGLL3P | 1.252 | 5.27 | 0.000046 | 0.00193 |
| 1555349_a_at | ITGB2 | 1.228 | 5.10 | 0.000512 | 0.00821 |
| 218611_at | IER5 | 1.197 | 5.44 | 0.000103 | 0.00305 |
| 222483_at | EFHD2 | 1.193 | 7.03 | 0.000003 | 0.00049 |
| 217962_at | NOP10 | 1.180 | 7.14 | 0.000167 | 0.00410 |
| 238267_s_at | NA | 1.164 | 5.13 | 0.000033 | 0.00162 |
| 216438_s_at | TMSB4X | 1.163 | 10.05 | 0.000195 | 0.00452 |
| 1554481_a_at | EPB41 | 1.159 | 6.18 | 0.000103 | 0.00305 |
| 209806_at | HIST1H2BK | 1.136 | 7.16 | 0.000611 | 0.00909 |
| 235163_at | MOB3A | 1.117 | 5.03 | 0.000167 | 0.00410 |
| 1555797_a_at | ARPC5 | 1.097 | 7.66 | 0.000125 | 0.00337 |
| 212014_x_at | CD44 | 1.080 | 6.70 | 0.000415 | 0.00718 |
| 203175_at | RHOG | 1.070 | 5.35 | 0.000022 | 0.00127 |
| 214512_s_at | SUB1 | 1.065 | 6.90 | 0.000017 | 0.00116 |
| 238669_at | PTGS1 | 1.061 | 5.27 | 0.000010 | 0.00091 |
| 211275_s_at | GYG1 | 1.060 | 6.28 | 0.000094 | 0.00290 |
| 223649_s_at | SLC25A39 | 1.051 | 5.85 | 0.000040 | 0.00177 |
| 204436_at | PLEKHO2 | 1.048 | 5.31 | 0.000162 | 0.00402 |
| 1565583_at | ZSCAN5A | 1.044 | 5.16 | 0.000001 | 0.00017 |
| 221269_s_at | SH3BGRL3 | 1.043 | 8.78 | 0.000036 | 0.00166 |
| 222686_s_at | CPPED1 | 1.042 | 5.82 | 0.000045 | 0.00189 |
| 215952_s_at | OAZ1 | 1.039 | 7.29 | 0.000006 | 0.00068 |
| 1557905_s_at | CD44 | 1.032 | 8.83 | 0.000421 | 0.00724 |
| 243749_s_at | LOC100507316 | 1.030 | 5.11 | 0.000005 | 0.00061 |
| 204336_s_at | RGS19 | 1.012 | 6.03 | 0.000409 | 0.00713 |
| 204319_s_at | RGS10 | 1.011 | 5.92 | 0.000228 | 0.00498 |
| 214847_s_at | GPSM3 | 1.000 | 5.02 | 0.000051 | 0.00203 |
| 209193_at | PIM1 | 0.991 | 6.57 | 0.000239 | 0.00513 |
| 200704_at | LITAF | 0.986 | 8.31 | 0.000581 | 0.00887 |
| 200859_x_at | FLNA | 0.981 | 5.31 | 0.000019 | 0.00120 |
| 208785_s_at | MAP1LC3B | 0.976 | 5.81 | 0.000586 | 0.00890 |
| 1556316_s_at | LOC284889 | 0.965 | 7.09 | 0.000041 | 0.00179 |
| 1563445_x_at | CTSLP8 | 0.962 | 6.02 | 0.000143 | 0.00371 |
| 223179_at | YPEL3 | 0.961 | 6.58 | 0.000551 | 0.00863 |
| 208960_s_at | KLF6 | 0.953 | 6.06 | 0.000590 | 0.00893 |
| 202002_at | ACAA2 | 0.952 | 5.14 | 0.000018 | 0.00118 |
| 201452_at | RHEB | 0.943 | 5.05 | 0.000001 | 0.00031 |
| 243099_at | NFAM1 | 0.941 | 5.67 | 0.000067 | 0.00236 |
| 1555478_at | C17orf82 | 0.934 | 5.65 | 0.000026 | 0.00139 |
| 1555730_a_at | CFL1 | 0.927 | 7.44 | 0.000162 | 0.00402 |
| 200921_s_at | BTG1 | 0.926 | 6.74 | 0.000671 | 0.00958 |
| 216835_s_at | DOK1 | 0.922 | 6.56 | 0.000028 | 0.00144 |
| 237041_x_at | RCOR1 | 0.921 | 5.45 | 0.000000 | 0.00012 |
| 231268_at | MYBL1 | 0.912 | 5.50 | 0.000344 | 0.00645 |
| 211672_s_at | ARPC4 /// ARPC4-TTLL3 | 0.909 | 7.94 | 0.000119 | 0.00328 |
| 224752_at | C7orf73 /// LOC101930655 | 0.884 | 6.76 | 0.000693 | 0.00977 |
| 225530_at | MOB3A | 0.879 | 5.30 | 0.000257 | 0.00535 |
| 218758_s_at | RRP1 | 0.879 | 5.56 | 0.000002 | 0.00033 |
| 228319_at | NA | 0.878 | 5.12 | 0.000035 | 0.00166 |
| 205967_at | HIST1H4A /// HIST1H4B /// HIST1H4C /// HIST1H4D /// HIST1H4E /// HIST1H4F /// HIST1H4H /// HIST1H4I /// HIST1H4J /// HIST1H4K /// HIST1H4L /// HIST2H4A /// HIST2H4B /// HIST4H4 | 0.878 | 6.24 | 0.000302 | 0.00590 |
| 206986_at | FGF18 | 0.874 | 5.29 | 0.000003 | 0.00045 |
| 214752_x_at | FLNA | 0.874 | 5.52 | 0.000040 | 0.00177 |
| 230046_at | SPRED3 | 0.869 | 5.18 | 0.000149 | 0.00380 |
| 200075_s_at | GUK1 | 0.867 | 7.44 | 0.000006 | 0.00069 |
| 208232_x_at | NRG1 | 0.863 | 5.37 | 0.000113 | 0.00319 |
| 208112_x_at | EHD1 | 0.854 | 5.71 | 0.000441 | 0.00741 |
| 209310_s_at | CASP4 | 0.851 | 6.17 | 0.000404 | 0.00708 |
| 218600_at | LIMD2 | 0.849 | 5.02 | 0.000112 | 0.00318 |
| 219183_s_at | CYTH4 | 0.846 | 5.35 | 0.000111 | 0.00318 |
| 41047_at | C9orf16 | 0.845 | 6.88 | 0.000235 | 0.00508 |
| 215454_x_at | SFTPC | 0.838 | 5.79 | 0.000019 | 0.00120 |
| 224182_x_at | SEMA6B | 0.833 | 5.14 | 0.000029 | 0.00146 |
| 204479_at | OSTF1 | 0.827 | 6.04 | 0.000650 | 0.00935 |
| 219893_at | CCDC71 | 0.822 | 5.01 | 0.000006 | 0.00065 |
| 201527_at | ATP6V1F /// LOC101927180 | 0.822 | 6.97 | 0.000042 | 0.00180 |
| 203433_at | MTHFS /// ST20-MTHFS | 0.820 | 5.57 | 0.000034 | 0.00164 |
| 202897_at | SIRPA | 0.818 | 5.60 | 0.000650 | 0.00935 |
| 239446_x_at | DCBLD2 | 0.814 | 5.17 | 0.000185 | 0.00436 |
| 239933_x_at | CCDC176 | 0.814 | 5.30 | 0.000015 | 0.00108 |
| 224846_at | SHKBP1 | 0.812 | 5.54 | 0.000112 | 0.00318 |
| 235863_at | JSRP1 | 0.810 | 5.31 | 0.000353 | 0.00653 |
| 222067_x_at | HIST1H2BD | 0.810 | 7.98 | 0.000680 | 0.00964 |
| 208002_s_at | ACOT7 | 0.810 | 5.59 | 0.000108 | 0.00312 |
| 202895_s_at | SIRPA | 0.807 | 5.08 | 0.000400 | 0.00704 |
| 226704_at | UBE2J2 | 0.802 | 5.04 | 0.000017 | 0.00114 |
| 206128_at | ADRA2C | 0.801 | 5.15 | 0.000049 | 0.00202 |
| 214534_at | HIST1H1B | 0.796 | 5.15 | 0.000301 | 0.00589 |
| 224637_at | OST4 | 0.795 | 7.85 | 0.000583 | 0.00889 |
| 228614_at | LINC00116 | 0.790 | 6.75 | 0.000443 | 0.00743 |
| 206687_s_at | PTPN6 | 0.786 | 6.81 | 0.000226 | 0.00494 |
| 219646_at | DEF8 | 0.786 | 5.44 | 0.000014 | 0.00106 |
| 224111_x_at | KLF16 | 0.785 | 5.24 | 0.000007 | 0.00071 |
| 221932_s_at | GLRX5 | 0.782 | 6.80 | 0.000608 | 0.00907 |
| 202201_at | BLVRB | 0.781 | 6.34 | 0.000445 | 0.00745 |
| 221141_x_at | EPN1 | 0.776 | 5.11 | 0.000021 | 0.00124 |
| 216611_s_at | SLC6A2 | 0.774 | 5.11 | 0.000089 | 0.00283 |
| 207641_at | TNFRSF13B | 0.771 | 5.63 | 0.000015 | 0.00108 |
| 231161_x_at | TRIM8 | 0.764 | 5.16 | 0.000607 | 0.00907 |
| 202460_s_at | LPIN2 | 0.757 | 5.59 | 0.000304 | 0.00592 |
| 229998_x_at | EVA1B | 0.748 | 5.30 | 0.000183 | 0.00433 |
| 218834_s_at | TMEM132A | 0.744 | 5.36 | 0.000094 | 0.00290 |
| 217635_s_at | POLG | 0.744 | 6.46 | 0.000014 | 0.00105 |
| 60471_at | RIN3 | 0.744 | 5.79 | 0.000199 | 0.00458 |
| 203879_at | PIK3CD | 0.742 | 5.87 | 0.000071 | 0.00249 |
| 213513_x_at | ARPC2 | 0.737 | 8.51 | 0.000014 | 0.00106 |
| 237570_x_at | NA | 0.735 | 5.06 | 0.000057 | 0.00217 |
| 212430_at | RBM38 | 0.734 | 5.77 | 0.000101 | 0.00302 |
| 216473_x_at | DBET /// DUX4 /// DUX4L1 /// DUX4L2 /// DUX4L24 /// DUX4L3 /// DUX4L4 /// DUX4L5 /// DUX4L6 /// DUX4L7 /// DUX4L8 /// LOC100288289 /// LOC100291626 /// LOC652301 | 0.728 | 5.55 | 0.000423 | 0.00724 |
| 205212_s_at | ACAP1 | 0.728 | 5.10 | 0.000217 | 0.00480 |
| 200021_at | CFL1 | 0.728 | 8.58 | 0.000345 | 0.00645 |
| 224854_s_at | SLAIN2 | 0.727 | 7.00 | 0.000052 | 0.00206 |
| 223064_at | RNF181 | 0.719 | 6.91 | 0.000710 | 0.00991 |
| 223626_x_at | IFI27L2 | 0.711 | 5.81 | 0.000237 | 0.00512 |
| 239663_x_at | LARP1 | 0.710 | 5.34 | 0.000021 | 0.00125 |
| 207289_at | MMP25 | 0.697 | 5.02 | 0.000359 | 0.00660 |
| 221700_s_at | UBA52 | 0.693 | 9.80 | 0.000324 | 0.00619 |
| 233974_s_at | FAM129B | 0.692 | 5.67 | 0.000085 | 0.00275 |
| 203777_s_at | RPS6KB2 | 0.691 | 5.83 | 0.000093 | 0.00289 |
| 241921_x_at | NA | 0.691 | 5.59 | 0.000193 | 0.00450 |
| 213892_s_at | APRT | 0.686 | 6.56 | 0.000098 | 0.00299 |
| 224947_at | RNF26 | 0.684 | 5.29 | 0.000105 | 0.00307 |
| 229826_at | SMIM4 | 0.680 | 5.82 | 0.000085 | 0.00275 |
| 214122_at | PDLIM7 | 0.677 | 5.13 | 0.000417 | 0.00720 |
| 210740_s_at | ITPK1 | 0.671 | 7.61 | 0.000003 | 0.00045 |
| 217818_s_at | ARPC4 | 0.671 | 6.72 | 0.000220 | 0.00483 |
| 213740_s_at | TMEM262 | 0.671 | 5.62 | 0.000093 | 0.00289 |
| 209413_at | B4GALT2 | 0.668 | 5.14 | 0.000061 | 0.00223 |
| 232438_at | EPS15L1 | 0.665 | 5.89 | 0.000176 | 0.00421 |
| 208919_s_at | NADK | 0.665 | 5.91 | 0.000640 | 0.00929 |
| 223050_s_at | FBXW5 | 0.664 | 5.19 | 0.000010 | 0.00090 |
| 218301_at | RNPEPL1 | 0.662 | 5.14 | 0.000392 | 0.00699 |
| 211226_at | GALR2 | 0.662 | 5.13 | 0.000585 | 0.00889 |
| 206361_at | PTGDR2 | 0.661 | 5.41 | 0.000017 | 0.00116 |
| 228074_at | ITPRIPL2 | 0.657 | 5.42 | 0.000082 | 0.00270 |
| 229690_at | FAM109A | 0.651 | 5.56 | 0.000137 | 0.00362 |
| 207150_at | SLC18A3 | 0.649 | 5.72 | 0.000012 | 0.00099 |
| 234021_at | EML2 | 0.647 | 5.66 | 0.000428 | 0.00729 |
| 231201_at | PTGER1 | 0.643 | 5.24 | 0.000062 | 0.00227 |
| 203891_s_at | DAPK3 /// MIR637 | 0.639 | 5.64 | 0.000251 | 0.00528 |
| 207077_at | CELA2B | 0.637 | 5.85 | 0.000036 | 0.00166 |
| 203055_s_at | ARHGEF1 /// LOC100505585 | 0.632 | 5.22 | 0.000443 | 0.00743 |
| 225883_at | ATG16L2 | 0.628 | 5.01 | 0.000206 | 0.00464 |
| 220757_s_at | MIR4746 /// UBXN6 | 0.628 | 5.03 | 0.000072 | 0.00249 |
| 211433_x_at | FAM214B | 0.626 | 5.39 | 0.000552 | 0.00863 |
| 207988_s_at | ARPC2 | 0.624 | 8.89 | 0.000266 | 0.00545 |
| 200077_s_at | OAZ1 | 0.620 | 9.31 | 0.000109 | 0.00312 |
| 218860_at | NOC4L | 0.620 | 5.08 | 0.000452 | 0.00755 |
| 237046_x_at | IL34 | 0.620 | 5.43 | 0.000492 | 0.00795 |
| 234669_x_at | C11orf30 | 0.619 | 5.30 | 0.000345 | 0.00645 |
| 201863_at | FAM32A | 0.618 | 6.87 | 0.000079 | 0.00264 |
| 207685_at | CRYBB3 | 0.617 | 5.11 | 0.000471 | 0.00774 |
| 216348_at | RPS17P5 /// RPS17P5 | 0.616 | 6.46 | 0.000587 | 0.00891 |
| 241799_x_at | LOC102725022 | 0.613 | 5.81 | 0.000143 | 0.00372 |
| 218112_at | MRPS34 | 0.610 | 5.93 | 0.000174 | 0.00420 |
| 1558723_at | LOC284014 | 0.609 | 5.59 | 0.000051 | 0.00203 |
| 205695_at | SDS | 0.606 | 5.29 | 0.000125 | 0.00337 |
| 214555_at | SSTR5 | 0.606 | 5.43 | 0.000021 | 0.00124 |
| 229217_at | SP3 | 0.605 | 5.42 | 0.000060 | 0.00223 |
| 1557120_at | EEF1A1 | 0.594 | 5.67 | 0.000039 | 0.00175 |
| 244191_at | RPLP1 | 0.593 | 5.15 | 0.000092 | 0.00289 |
| 225454_at | CCDC124 | 0.591 | 5.28 | 0.000477 | 0.00781 |
| 204357_s_at | LIMK1 | 0.589 | 5.89 | 0.000281 | 0.00565 |
| 217389_s_at | ATF5 | 0.585 | 5.84 | 0.000522 | 0.00831 |
| 223147_s_at | SFT2D3 /// WDR33 | 0.584 | 5.78 | 0.000625 | 0.00921 |
| 205983_at | DPEP1 | 0.578 | 5.11 | 0.000033 | 0.00161 |
| 220256_s_at | OXCT2 | 0.576 | 5.20 | 0.000512 | 0.00821 |
| 226870_at | COMTD1 | 0.570 | 5.42 | 0.000678 | 0.00963 |
| 208569_at | HIST1H2AB | 0.567 | 5.20 | 0.000198 | 0.00457 |
| 236030_at | RCOR2 | 0.563 | 5.31 | 0.000616 | 0.00914 |
| 223247_at | MED10 | 0.559 | 5.34 | 0.000470 | 0.00774 |
| 213613_s_at | NADK | 0.559 | 5.53 | 0.000563 | 0.00869 |
| 212315_s_at | NUP210 | 0.558 | 5.36 | 0.000058 | 0.00217 |
| 244707_at | RP11-272D12.1 | 0.556 | 5.14 | 0.000168 | 0.00411 |
| 230627_at | NA | 0.556 | 5.29 | 0.000640 | 0.00929 |
| 207475_at | FABP2 | 0.556 | 5.29 | 0.000195 | 0.00451 |
| 226629_at | SLC43A2 | 0.552 | 5.87 | 0.000398 | 0.00703 |
| 244504_x_at | ARF1 /// MIR3620 | 0.549 | 5.11 | 0.000677 | 0.00963 |
| 236894_at | L1TD1 | 0.547 | 5.06 | 0.000597 | 0.00900 |
| 229758_at | TIGD5 | 0.547 | 5.37 | 0.000586 | 0.00890 |
| 203522_at | CCS | 0.543 | 5.12 | 0.000276 | 0.00559 |
| 225091_at | ZCCHC3 | 0.543 | 6.14 | 0.000633 | 0.00927 |
| 218753_at | XKR8 | 0.537 | 5.73 | 0.000476 | 0.00781 |
| 243267_x_at | NA | 0.534 | 5.22 | 0.000645 | 0.00931 |
| 221791_s_at | TMA7 | 0.534 | 9.76 | 0.000581 | 0.00887 |
| 222164_at | FGFR1 | 0.533 | 6.47 | 0.000601 | 0.00903 |
| 217313_at | AC004692.5 | 0.532 | 6.23 | 0.000572 | 0.00880 |
| 216821_at | KRT8P11 /// KRT8P11 | 0.530 | 5.68 | 0.000437 | 0.00740 |
| 223153_x_at | TMUB1 | 0.528 | 5.53 | 0.000210 | 0.00469 |
| 221769_at | SPSB3 | 0.521 | 5.52 | 0.000232 | 0.00505 |
| 1553082_at | CRYGN | 0.521 | 5.30 | 0.000120 | 0.00330 |
| 211716_x_at | ARHGDIA | 0.512 | 6.68 | 0.000292 | 0.00579 |
| 222861_x_at | FBXO44 | 0.494 | 5.80 | 0.000584 | 0.00889 |
| 210457_x_at | HMGA1 | 0.494 | 6.34 | 0.000194 | 0.00451 |
| 220973_s_at | SHARPIN | 0.492 | 6.24 | 0.000697 | 0.00981 |
| 210811_s_at | DDX49 | 0.489 | 6.11 | 0.000608 | 0.00907 |
| 226466_s_at | FAM58A | 0.487 | 6.64 | 0.000065 | 0.00233 |
| 222818_at | OSBPL10 | 0.481 | 5.39 | 0.000550 | 0.00862 |
| 213987_s_at | CDK13 | 0.479 | 5.95 | 0.000319 | 0.00612 |
| 226253_at | LRRC45 | 0.473 | 5.00 | 0.000200 | 0.00458 |
| 209367_at | STXBP2 | 0.446 | 6.71 | 0.000558 | 0.00866 |
| 204335_at | CCDC94 | 0.442 | 5.45 | 0.000415 | 0.00718 |
| 225148_at | RPS19BP1 | 0.412 | 6.86 | 0.000557 | 0.00866 |
| 1560439_at | LINGO3 | 0.400 | 5.85 | 0.000180 | 0.00428 |
| 1553567_s_at | ATP6 | 0.354 | 11.96 | 0.000424 | 0.00724 |
| 224375_at | LOC102724870 | 0.326 | 11.97 | 0.000637 | 0.00927 |
| 226052_at | BRD4 | -0.432 | 8.91 | 0.000295 | 0.00582 |
| 204658_at | TRA2A | -0.451 | 7.73 | 0.000480 | 0.00785 |
| 222489_s_at | WRNIP1 | -0.478 | 9.01 | 0.000344 | 0.00645 |
| 1554678_s_at | HNRNPDL | -0.503 | 8.77 | 0.000171 | 0.00415 |
| 1294_at | MIR5193 /// UBA7 | -0.503 | 6.66 | 0.000469 | 0.00773 |
| 216836_s_at | ERBB2 | -0.504 | 7.57 | 0.000485 | 0.00790 |
| 200058_s_at | LOC101929240 /// SNRNP200 | -0.515 | 7.44 | 0.000174 | 0.00420 |
| 209272_at | NAB1 | -0.516 | 6.23 | 0.000680 | 0.00964 |
| 200033_at | DDX5 /// MIR3064 /// MIR5047 | -0.519 | 9.01 | 0.000249 | 0.00526 |
| 211962_s_at | ZFP36L1 | -0.520 | 9.48 | 0.000547 | 0.00860 |
| 200041_s_at | ATP6V1G2-DDX39B /// DDX39B /// SNORD84 | -0.520 | 7.47 | 0.000034 | 0.00162 |
| 217895_at | PTCD3 | -0.521 | 6.05 | 0.000207 | 0.00466 |
| 1557227_s_at | TPR | -0.521 | 8.11 | 0.000676 | 0.00963 |
| 222521_x_at | NDUFC2 /// NDUFC2-KCTD14 | -0.529 | 6.69 | 0.000225 | 0.00493 |
| 203056_s_at | PRDM2 | -0.530 | 7.52 | 0.000386 | 0.00694 |
| 207435_s_at | SRRM2 | -0.531 | 6.48 | 0.000019 | 0.00121 |
| 201057_s_at | GOLGB1 | -0.536 | 7.25 | 0.000050 | 0.00203 |
| 217580_x_at | NA | -0.547 | 7.80 | 0.000426 | 0.00726 |
| 209127_s_at | SART3 | -0.549 | 7.99 | 0.000181 | 0.00430 |
| 1563560_at | NA | -0.551 | 9.89 | 0.000100 | 0.00301 |
| 37425_g_at | CCHCR1 | -0.553 | 5.05 | 0.000701 | 0.00984 |
| 222244_s_at | TUG1 | -0.554 | 6.81 | 0.000297 | 0.00584 |
| 218808_at | DALRD3 | -0.555 | 5.87 | 0.000658 | 0.00942 |
| 214356_s_at | KIAA0368 | -0.557 | 7.77 | 0.000057 | 0.00217 |
| 1556924_at | CFLAR-AS1 | -0.560 | 5.94 | 0.000634 | 0.00927 |
| 220725_x_at | DNAH3 | -0.560 | 8.93 | 0.000627 | 0.00922 |
| 206936_x_at | NA | -0.562 | 9.45 | 0.000389 | 0.00697 |
| 243579_at | MSI2 | -0.565 | 7.67 | 0.000456 | 0.00758 |
| 226465_s_at | SON | -0.569 | 9.61 | 0.000045 | 0.00189 |
| 208804_s_at | SRSF6 | -0.570 | 7.08 | 0.000464 | 0.00767 |
| 214988_s_at | SON | -0.573 | 7.45 | 0.000027 | 0.00141 |
| 201247_at | SREBF2 | -0.580 | 6.85 | 0.000268 | 0.00548 |
| 219226_at | CDK12 | -0.581 | 6.16 | 0.000620 | 0.00917 |
| 203513_at | SPG11 | -0.582 | 6.49 | 0.000641 | 0.00929 |
| 1555743_s_at | ERVH-6 | -0.587 | 6.33 | 0.000493 | 0.00797 |
| 208779_x_at | DDR1 /// MIR4640 | -0.587 | 8.51 | 0.000146 | 0.00377 |
| 217734_s_at | WDR6 | -0.588 | 8.81 | 0.000280 | 0.00564 |
| 203380_x_at | SRSF5 | -0.589 | 9.13 | 0.000157 | 0.00392 |
| 207169_x_at | DDR1 /// MIR4640 | -0.590 | 8.23 | 0.000048 | 0.00199 |
| 225181_at | ARID1B | -0.591 | 6.58 | 0.000706 | 0.00989 |
| 228487_s_at | NA | -0.592 | 6.93 | 0.000259 | 0.00536 |
| 201086_x_at | SON | -0.593 | 7.18 | 0.000033 | 0.00161 |
| 225117_at | KANSL1 | -0.596 | 6.89 | 0.000094 | 0.00290 |
| 222021_x_at | SDHAP1 /// SDHAP2 | -0.598 | 8.06 | 0.000012 | 0.00096 |
| 1556178_x_at | TAF8 | -0.599 | 6.27 | 0.000037 | 0.00170 |
| 244272_s_at | TC2N | -0.605 | 8.44 | 0.000366 | 0.00668 |
| 216221_s_at | PUM2 | -0.607 | 6.81 | 0.000212 | 0.00471 |
| 224614_at | DYNC1LI2 | -0.607 | 6.33 | 0.000574 | 0.00883 |
| 204064_at | THOC1 | -0.607 | 6.03 | 0.000441 | 0.00741 |
| 226691_at | TNRC18 | -0.610 | 6.62 | 0.000708 | 0.00989 |
| 211277_x_at | APP | -0.612 | 9.24 | 0.000073 | 0.00251 |
| 212266_s_at | SRSF5 | -0.613 | 7.53 | 0.000233 | 0.00506 |
| 223577_x_at | MALAT1 | -0.614 | 11.82 | 0.000099 | 0.00300 |
| 235244_at | CCDC58 | -0.615 | 6.71 | 0.000255 | 0.00534 |
| 225139_at | NFATC3 | -0.615 | 7.35 | 0.000178 | 0.00426 |
| 204113_at | CELF1 | -0.617 | 8.07 | 0.000175 | 0.00420 |
| 202167_s_at | MMS19 | -0.620 | 6.35 | 0.000626 | 0.00921 |
| 202462_s_at | DDX46 | -0.620 | 6.06 | 0.000045 | 0.00189 |
| 201951_at | ALCAM | -0.621 | 8.49 | 0.000431 | 0.00733 |
| 212451_at | SECISBP2L | -0.621 | 7.14 | 0.000261 | 0.00536 |
| 209431_s_at | PATZ1 | -0.624 | 5.05 | 0.000577 | 0.00885 |
| 213762_x_at | LOC101928747 /// RBMX /// SNORD61 | -0.627 | 7.81 | 0.000206 | 0.00464 |
| 210749_x_at | DDR1 /// MIR4640 | -0.628 | 8.23 | 0.000046 | 0.00191 |
| 219121_s_at | ESRP1 | -0.628 | 7.58 | 0.000635 | 0.00927 |
| 226898_s_at | SFPQ | -0.629 | 6.75 | 0.000084 | 0.00273 |
| 207186_s_at | BPTF | -0.629 | 7.13 | 0.000238 | 0.00513 |
| 235281_x_at | AHNAK | -0.631 | 9.59 | 0.000017 | 0.00114 |
| 229586_at | CHD9 | -0.633 | 7.20 | 0.000518 | 0.00828 |
| 208663_s_at | TTC3 /// TTC3P1 | -0.635 | 7.36 | 0.000177 | 0.00423 |
| 225705_at | CEP95 | -0.635 | 6.32 | 0.000258 | 0.00535 |
| 214744_s_at | RPL23 /// SNORA21 | -0.636 | 5.81 | 0.000242 | 0.00516 |
| 214118_x_at | PCM1 | -0.637 | 8.15 | 0.000054 | 0.00211 |
| 202809_s_at | INTS3 | -0.638 | 6.57 | 0.000445 | 0.00745 |
| 223016_x_at | ZRANB2 | -0.639 | 6.06 | 0.000579 | 0.00887 |
| 200648_s_at | GLUL | -0.639 | 10.31 | 0.000161 | 0.00402 |
| 230926_s_at | ODF2L | -0.640 | 8.09 | 0.000217 | 0.00480 |
| 1007_s_at | DDR1 /// MIR4640 | -0.641 | 9.14 | 0.000020 | 0.00123 |
| 212854_x_at | NBPF1 | -0.644 | 8.28 | 0.000021 | 0.00125 |
| 242602_x_at | ZNF254 | -0.645 | 7.92 | 0.000011 | 0.00092 |
| 208073_x_at | TTC3 /// TTC3P1 | -0.646 | 7.42 | 0.000106 | 0.00308 |
| 200635_s_at | PTPRF | -0.649 | 6.74 | 0.000072 | 0.00249 |
| 215504_x_at | ANKRD10-IT1 | -0.652 | 8.65 | 0.000333 | 0.00632 |
| 225569_at | AGO2 /// CASC7 /// CASC7 | -0.653 | 6.38 | 0.000167 | 0.00410 |
| 226520_at | LCOR | -0.653 | 6.76 | 0.000203 | 0.00461 |
| 217234_s_at | EZR | -0.654 | 10.22 | 0.000061 | 0.00223 |
| 64474_g_at | DGCR8 /// MIR1306 | -0.654 | 5.29 | 0.000219 | 0.00482 |
| 201736_s_at | 38777 | -0.654 | 8.49 | 0.000063 | 0.00227 |
| 227465_at | MAU2 | -0.656 | 6.59 | 0.000203 | 0.00461 |
| 225598_at | SLC45A4 | -0.657 | 6.05 | 0.000520 | 0.00830 |
| 223713_at | RSPH3 | -0.658 | 7.87 | 0.000285 | 0.00569 |
| 208988_at | KDM2A | -0.660 | 6.55 | 0.000556 | 0.00866 |
| 202810_at | DRG1 | -0.660 | 6.37 | 0.000519 | 0.00829 |
| 214937_x_at | PCM1 | -0.661 | 8.18 | 0.000064 | 0.00229 |
| 202519_at | MLXIP | -0.662 | 5.98 | 0.000344 | 0.00645 |
| 208930_s_at | ILF3 | -0.663 | 6.74 | 0.000055 | 0.00214 |
| 217202_s_at | GLUL | -0.665 | 9.18 | 0.000483 | 0.00788 |
| 208936_x_at | LGALS8 | -0.666 | 6.51 | 0.000654 | 0.00937 |
| 233093_s_at | BIRC6 | -0.667 | 6.51 | 0.000629 | 0.00923 |
| 211778_s_at | OVOL2 | -0.669 | 5.97 | 0.000390 | 0.00697 |
| 225869_s_at | UNC93B1 | -0.673 | 5.89 | 0.000168 | 0.00411 |
| 213470_s_at | HNRNPH1 | -0.673 | 7.90 | 0.000696 | 0.00980 |
| 201474_s_at | ITGA3 | -0.675 | 5.37 | 0.000437 | 0.00740 |
| 213775_x_at | ZNF638 /// ZNF638-IT1 | -0.675 | 7.04 | 0.000027 | 0.00141 |
| 208775_at | XPO1 | -0.677 | 7.41 | 0.000103 | 0.00306 |
| 223286_at | ELP5 | -0.678 | 5.40 | 0.000278 | 0.00561 |
| 216060_s_at | DAAM1 | -0.678 | 5.86 | 0.000311 | 0.00599 |
| 212721_at | SREK1 | -0.678 | 6.29 | 0.000598 | 0.00900 |
| 226129_at | FAM83H | -0.679 | 6.49 | 0.000181 | 0.00430 |
| 214552_s_at | RABEP1 | -0.680 | 5.67 | 0.000139 | 0.00365 |
| 202622_s_at | ATXN2 | -0.682 | 6.53 | 0.000324 | 0.00619 |
| 211257_x_at | ZNF638 | -0.682 | 6.52 | 0.000357 | 0.00657 |
| 224159_x_at | TRIM4 | -0.683 | 8.76 | 0.000591 | 0.00893 |
| 228926_s_at | SMARCA2 | -0.686 | 6.94 | 0.000152 | 0.00386 |
| 214077_x_at | MEIS3P1 | -0.687 | 8.38 | 0.000287 | 0.00573 |
| 203357_s_at | CAPN7 | -0.689 | 6.31 | 0.000394 | 0.00699 |
| 244768_at | DYNC1H1 | -0.690 | 6.54 | 0.000345 | 0.00645 |
| 202267_at | LAMC2 | -0.690 | 5.47 | 0.000424 | 0.00725 |
| 224336_s_at | DUSP16 | -0.691 | 5.11 | 0.000438 | 0.00741 |
| 225358_at | DNAJC19 | -0.692 | 6.28 | 0.000331 | 0.00627 |
| 203997_at | PTPN3 | -0.692 | 6.35 | 0.000358 | 0.00658 |
| 232324_x_at | NA | -0.692 | 5.19 | 0.000491 | 0.00794 |
| 218740_s_at | CDK5RAP3 | -0.693 | 7.33 | 0.000393 | 0.00699 |
| 213594_x_at | SRSF10 | -0.693 | 6.91 | 0.000399 | 0.00703 |
| 223165_s_at | IP6K2 | -0.694 | 6.27 | 0.000558 | 0.00866 |
| 233732_at | LOC401320 | -0.698 | 6.17 | 0.000635 | 0.00927 |
| 208407_s_at | CTNND1 /// TMX2-CTNND1 | -0.698 | 7.14 | 0.000314 | 0.00605 |
| 232323_s_at | TTC17 | -0.699 | 5.99 | 0.000503 | 0.00809 |
| 241632_x_at | NA | -0.702 | 8.73 | 0.000643 | 0.00930 |
| 229666_s_at | CSTF3 | -0.703 | 7.98 | 0.000340 | 0.00641 |
| 54037_at | HPS4 | -0.703 | 6.18 | 0.000239 | 0.00513 |
| 241683_at | HECTD1 | -0.703 | 6.95 | 0.000077 | 0.00259 |
| 212693_at | MDN1 | -0.705 | 5.79 | 0.000280 | 0.00564 |
| 229533_x_at | ZNF680 | -0.707 | 6.73 | 0.000270 | 0.00550 |
| 206179_s_at | TPPP | -0.708 | 7.89 | 0.000228 | 0.00498 |
| 212217_at | PREPL | -0.708 | 5.68 | 0.000525 | 0.00835 |
| 242961_x_at | DDX58 | -0.708 | 7.42 | 0.000664 | 0.00949 |
| 35436_at | GOLGA2 | -0.708 | 6.03 | 0.000597 | 0.00900 |
| 228095_at | PHF14 | -0.711 | 5.12 | 0.000439 | 0.00741 |
| 208863_s_at | SRSF1 | -0.712 | 7.80 | 0.000362 | 0.00664 |
| 1554411_at | CTNNB1 | -0.712 | 8.12 | 0.000118 | 0.00326 |
| 201636_at | FXR1 | -0.714 | 6.02 | 0.000690 | 0.00974 |
| 238189_at | SIN3A | -0.716 | 5.29 | 0.000399 | 0.00703 |
| 215090_x_at | LOC440434 | -0.719 | 6.28 | 0.000081 | 0.00269 |
| 230662_at | RNF187 | -0.719 | 5.21 | 0.000060 | 0.00221 |
| 222700_at | ATL2 | -0.719 | 5.05 | 0.000461 | 0.00763 |
| 203035_s_at | PIAS3 | -0.722 | 6.38 | 0.000093 | 0.00290 |
| 206958_s_at | UPF3A | -0.722 | 5.46 | 0.000167 | 0.00410 |
| 213505_s_at | SUGP2 | -0.722 | 6.67 | 0.000016 | 0.00114 |
| 202171_at | VEZF1 | -0.723 | 6.01 | 0.000618 | 0.00915 |
| 221971_x_at | AGAP10 /// AGAP11 /// AGAP4 /// AGAP5 /// AGAP9 /// BMS1P5 /// CTGLF12P /// LOC101060581 | -0.723 | 6.75 | 0.000577 | 0.00885 |
| 216279_at | ZNF460 | -0.724 | 7.23 | 0.000328 | 0.00623 |
| 1556176_at | TAF8 | -0.728 | 6.05 | 0.000012 | 0.00096 |
| 205788_s_at | LOC441155 /// ZC3H11A | -0.733 | 8.15 | 0.000025 | 0.00137 |
| 222369_at | NAA40 | -0.733 | 6.55 | 0.000132 | 0.00353 |
| 226445_s_at | TRIM41 | -0.736 | 7.08 | 0.000408 | 0.00713 |
| 210676_x_at | RGPD3 /// RGPD4 /// RGPD5 /// RGPD6 /// RGPD8 | -0.740 | 6.27 | 0.000271 | 0.00550 |
| 223578_x_at | MALAT1 | -0.742 | 10.86 | 0.000122 | 0.00335 |
| 200857_s_at | NCOR1 | -0.742 | 5.44 | 0.000431 | 0.00732 |
| 225617_at | ODF2 | -0.744 | 6.00 | 0.000534 | 0.00845 |
| 224558_s_at | MALAT1 | -0.746 | 11.84 | 0.000033 | 0.00162 |
| 218396_at | VPS13C | -0.746 | 7.77 | 0.000007 | 0.00071 |
| 212065_s_at | USP34 | -0.746 | 6.14 | 0.000284 | 0.00569 |
| 231876_at | TRIM56 | -0.747 | 7.49 | 0.000717 | 0.00998 |
| 1557290_at | DPY19L2P4 | -0.748 | 6.17 | 0.000550 | 0.00862 |
| 1554190_s_at | PLEKHS1 | -0.749 | 8.52 | 0.000027 | 0.00141 |
| 1555106_a_at | CTDSPL2 | -0.749 | 6.01 | 0.000684 | 0.00967 |
| 234725_s_at | SEMA4B | -0.750 | 7.11 | 0.000560 | 0.00867 |
| 215718_s_at | PHF3 | -0.750 | 7.12 | 0.000036 | 0.00166 |
| 203987_at | FZD6 | -0.751 | 5.93 | 0.000647 | 0.00933 |
| 209337_at | PSIP1 | -0.751 | 6.28 | 0.000155 | 0.00390 |
| 229044_at | NUDT17 | -0.751 | 7.42 | 0.000057 | 0.00217 |
| 212428_at | KIAA0368 | -0.753 | 6.31 | 0.000061 | 0.00223 |
| 205095_s_at | ATP6V0A1 | -0.753 | 5.20 | 0.000687 | 0.00970 |
| 209997_x_at | PCM1 | -0.753 | 7.63 | 0.000106 | 0.00309 |
| 206323_x_at | OPHN1 | -0.754 | 11.72 | 0.000022 | 0.00127 |
| 204325_s_at | LOC101930150 /// NF1 | -0.755 | 5.77 | 0.000590 | 0.00893 |
| 242196_at | ARHGAP32 | -0.756 | 7.11 | 0.000213 | 0.00472 |
| 230894_s_at | NA | -0.757 | 8.57 | 0.000100 | 0.00302 |
| 212198_s_at | TM9SF4 | -0.757 | 6.94 | 0.000601 | 0.00903 |
| 222455_s_at | PARVA | -0.759 | 6.32 | 0.000283 | 0.00567 |
| 204498_s_at | ADCY9 | -0.759 | 6.16 | 0.000269 | 0.00549 |
| 224842_at | BOLA2 /// LOC101060386 /// SMG1 | -0.759 | 5.30 | 0.000399 | 0.00703 |
| 225340_s_at | CAPRIN1 | -0.760 | 7.29 | 0.000022 | 0.00126 |
| 203038_at | PTPRK | -0.764 | 5.35 | 0.000651 | 0.00936 |
| 210645_s_at | TTC3 /// TTC3P1 | -0.764 | 7.04 | 0.000073 | 0.00252 |
| 201561_s_at | CLSTN1 | -0.765 | 8.30 | 0.000021 | 0.00125 |
| 201967_at | RBM6 | -0.765 | 6.27 | 0.000636 | 0.00927 |
| 1562697_at | LOC339988 | -0.766 | 5.86 | 0.000094 | 0.00290 |
| 209856_x_at | ABI2 | -0.766 | 5.09 | 0.000327 | 0.00622 |
| 243868_at | RFX3 | -0.767 | 7.65 | 0.000582 | 0.00887 |
| 227635_at | RBBP6 | -0.767 | 6.36 | 0.000021 | 0.00124 |
| 228175_at | SLC4A8 | -0.768 | 5.57 | 0.000087 | 0.00279 |
| 233475_at | MGC32805 | -0.769 | 5.04 | 0.000324 | 0.00619 |
| 204663_at | ME3 | -0.769 | 6.15 | 0.000452 | 0.00754 |
| 201056_at | GOLGB1 | -0.773 | 6.07 | 0.000272 | 0.00552 |
| 242123_at | PAQR7 | -0.774 | 7.44 | 0.000401 | 0.00706 |
| 212943_at | C2CD5 | -0.774 | 7.05 | 0.000691 | 0.00975 |
| 206734_at | JRKL | -0.775 | 5.10 | 0.000043 | 0.00186 |
| 237249_at | KCNQ1OT1 /// LOC101927338 | -0.778 | 5.14 | 0.000310 | 0.00598 |
| 236254_at | VPS13B | -0.780 | 5.83 | 0.000248 | 0.00525 |
| 228582_x_at | MALAT1 | -0.781 | 11.49 | 0.000162 | 0.00402 |
| 212504_at | DIP2C | -0.783 | 5.14 | 0.000065 | 0.00233 |
| 210958_s_at | MAST4 | -0.785 | 5.97 | 0.000085 | 0.00275 |
| 213022_s_at | UTRN | -0.785 | 6.63 | 0.000393 | 0.00699 |
| 212493_s_at | SETD2 | -0.786 | 5.90 | 0.000040 | 0.00178 |
| 212216_at | PREPL | -0.787 | 5.83 | 0.000234 | 0.00508 |
| 225560_at | POMT2 | -0.787 | 5.08 | 0.000065 | 0.00233 |
| 227643_at | TPPP | -0.789 | 7.81 | 0.000100 | 0.00301 |
| 225964_at | ZXDC | -0.790 | 5.64 | 0.000022 | 0.00126 |
| 220044_x_at | LUC7L3 | -0.790 | 7.60 | 0.000001 | 0.00028 |
| 209271_at | BPTF | -0.792 | 5.87 | 0.000473 | 0.00777 |
| 212842_x_at | RGPD3 /// RGPD4 /// RGPD5 /// RGPD6 /// RGPD8 | -0.794 | 6.10 | 0.000491 | 0.00794 |
| 225081_s_at | CDCA7L | -0.795 | 6.63 | 0.000133 | 0.00355 |
| 223187_s_at | ORMDL1 | -0.798 | 5.19 | 0.000161 | 0.00402 |
| 202761_s_at | SYNE2 | -0.799 | 7.35 | 0.000027 | 0.00141 |
| 202813_at | TARBP1 | -0.799 | 5.68 | 0.000105 | 0.00307 |
| 212848_s_at | C9orf3 | -0.800 | 6.33 | 0.000107 | 0.00309 |
| 200636_s_at | PTPRF | -0.801 | 7.74 | 0.000104 | 0.00306 |
| 224227_s_at | BDP1 | -0.805 | 5.81 | 0.000204 | 0.00461 |
| 218248_at | FAM111A | -0.805 | 7.98 | 0.000342 | 0.00644 |
| 204323_x_at | LOC101930150 /// NF1 | -0.806 | 6.36 | 0.000394 | 0.00699 |
| 221526_x_at | PARD3 | -0.808 | 6.41 | 0.000094 | 0.00290 |
| 207836_s_at | RBPMS | -0.808 | 5.71 | 0.000064 | 0.00231 |
| 228998_at | TNRC6B | -0.808 | 5.98 | 0.000347 | 0.00647 |
| 209558_s_at | HIP1R | -0.808 | 6.45 | 0.000558 | 0.00866 |
| 208614_s_at | FLNB | -0.809 | 8.64 | 0.000077 | 0.00259 |
| 237768_x_at | NA | -0.810 | 7.55 | 0.000217 | 0.00480 |
| 221884_at | MECOM | -0.811 | 6.45 | 0.000257 | 0.00535 |
| 218338_at | PHC1 | -0.813 | 6.36 | 0.000123 | 0.00335 |
| 1558620_at | ZNF621 | -0.815 | 5.46 | 0.000366 | 0.00668 |
| 210389_x_at | TUBD1 | -0.815 | 5.00 | 0.000016 | 0.00114 |
| 201996_s_at | SPEN | -0.817 | 6.31 | 0.000305 | 0.00593 |
| 239735_at | NA | -0.818 | 5.42 | 0.000248 | 0.00525 |
| 222339_x_at | NA | -0.818 | 7.71 | 0.000358 | 0.00658 |
| 227025_at | PPHLN1 | -0.820 | 5.61 | 0.000382 | 0.00690 |
| 231451_s_at | NA | -0.820 | 5.10 | 0.000256 | 0.00534 |
| 204020_at | PURA | -0.821 | 5.97 | 0.000142 | 0.00369 |
| 229665_at | CSTF3 | -0.821 | 6.28 | 0.000324 | 0.00619 |
| 1567213_at | PNN | -0.822 | 6.56 | 0.000438 | 0.00741 |
| 225221_at | ZKSCAN1 | -0.824 | 8.35 | 0.000101 | 0.00303 |
| 214703_s_at | MAN2B2 | -0.827 | 6.77 | 0.000061 | 0.00224 |
| 210094_s_at | PARD3 | -0.829 | 6.63 | 0.000552 | 0.00863 |
| 206335_at | GALNS | -0.829 | 7.22 | 0.000038 | 0.00173 |
| 234173_s_at | NXF2 /// NXF2B | -0.830 | 5.72 | 0.000024 | 0.00133 |
| 219017_at | ETNK1 | -0.830 | 5.64 | 0.000116 | 0.00324 |
| 219905_at | ERMAP | -0.831 | 5.48 | 0.000148 | 0.00380 |
| 209390_at | TSC1 | -0.833 | 5.72 | 0.000627 | 0.00922 |
| 231500_s_at | BOLA2 | -0.835 | 9.39 | 0.000297 | 0.00584 |
| 224984_at | NFAT5 | -0.835 | 6.71 | 0.000116 | 0.00324 |
| 218617_at | TRIT1 | -0.835 | 6.68 | 0.000194 | 0.00451 |
| 224685_at | MLLT4 | -0.837 | 7.68 | 0.000129 | 0.00345 |
| 220691_at | NA | -0.837 | 6.08 | 0.000402 | 0.00707 |
| 209240_at | OGT | -0.838 | 7.01 | 0.000232 | 0.00504 |
| 208719_s_at | DDX17 | -0.838 | 8.83 | 0.000014 | 0.00103 |
| 221538_s_at | PLXNA1 | -0.838 | 6.45 | 0.000350 | 0.00651 |
| 236017_at | CDKL3 | -0.838 | 5.94 | 0.000598 | 0.00900 |
| 32137_at | JAG2 | -0.841 | 6.31 | 0.000425 | 0.00725 |
| 224849_at | TTC17 | -0.842 | 5.84 | 0.000399 | 0.00703 |
| 218098_at | ARFGEF2 | -0.842 | 5.56 | 0.000025 | 0.00137 |
| 222169_x_at | SH2D3A | -0.842 | 6.31 | 0.000098 | 0.00299 |
| 202699_s_at | TMEM63A | -0.842 | 5.47 | 0.000033 | 0.00162 |
| 223793_at | PRRC2B | -0.842 | 6.49 | 0.000259 | 0.00536 |
| 216702_x_at | NA | -0.843 | 5.27 | 0.000649 | 0.00933 |
| 212650_at | EHBP1 | -0.845 | 6.62 | 0.000244 | 0.00519 |
| 40016_g_at | MAST4 | -0.848 | 6.51 | 0.000136 | 0.00362 |
| 213376_at | ZBTB1 | -0.849 | 5.43 | 0.000529 | 0.00840 |
| 202126_at | PRPF4B | -0.849 | 6.16 | 0.000026 | 0.00139 |
| 202773_s_at | SFSWAP | -0.850 | 7.06 | 0.000016 | 0.00112 |
| 202220_at | KIAA0907 | -0.850 | 7.49 | 0.000105 | 0.00307 |
| 221546_at | PRPF18 | -0.851 | 5.34 | 0.000104 | 0.00306 |
| 243222_at | ALPK1 | -0.851 | 5.13 | 0.000143 | 0.00372 |
| 232403_at | SPG11 | -0.853 | 6.44 | 0.000126 | 0.00337 |
| 227831_at | TMEM245 | -0.855 | 5.83 | 0.000496 | 0.00800 |
| 209234_at | KIF1B | -0.855 | 5.61 | 0.000125 | 0.00337 |
| 220008_at | PEAK1 | -0.855 | 5.78 | 0.000201 | 0.00460 |
| 213092_x_at | DNAJC9 | -0.856 | 5.80 | 0.000635 | 0.00927 |
| 210686_x_at | SLC25A16 | -0.856 | 9.46 | 0.000108 | 0.00312 |
| 217660_at | MYH14 | -0.857 | 5.92 | 0.000149 | 0.00380 |
| 220409_at | CAMSAP1 | -0.857 | 6.29 | 0.000611 | 0.00909 |
| 237444_at | KIF13A | -0.858 | 7.47 | 0.000050 | 0.00203 |
| 241955_at | HECTD1 | -0.858 | 8.69 | 0.000006 | 0.00066 |
| 213656_s_at | KLC1 | -0.860 | 6.68 | 0.000210 | 0.00469 |
| 209049_s_at | ZMYND8 | -0.860 | 6.25 | 0.000012 | 0.00096 |
| 1557232_at | LOC102725017 | -0.861 | 5.51 | 0.000253 | 0.00531 |
| 200637_s_at | PTPRF | -0.861 | 6.67 | 0.000308 | 0.00597 |
| 229994_at | NFIA | -0.864 | 7.33 | 0.000080 | 0.00265 |
| 212074_at | SUN1 | -0.864 | 7.81 | 0.000197 | 0.00455 |
| 1554873_at | CSPP1 | -0.864 | 7.64 | 0.000113 | 0.00318 |
| 242696_at | NA | -0.865 | 5.73 | 0.000222 | 0.00488 |
| 214600_at | TEAD1 | -0.865 | 6.31 | 0.000408 | 0.00713 |
| 207486_x_at | CHN2 | -0.866 | 6.06 | 0.000579 | 0.00887 |
| 214323_s_at | UPF3A | -0.866 | 7.65 | 0.000007 | 0.00072 |
| 1555808_a_at | EXD2 | -0.867 | 5.29 | 0.000210 | 0.00469 |
| 226563_at | SMAD2 | -0.867 | 5.25 | 0.000490 | 0.00794 |
| 225331_at | CCDC50 | -0.870 | 5.33 | 0.000365 | 0.00668 |
| 241973_x_at | DPP7 | -0.871 | 5.32 | 0.000056 | 0.00216 |
| 242417_at | LOC283278 /// PLEKHA7 | -0.873 | 5.81 | 0.000155 | 0.00390 |
| 229519_at | FXR1 | -0.874 | 6.09 | 0.000040 | 0.00178 |
| 227199_at | DIP2A | -0.875 | 5.32 | 0.000279 | 0.00563 |
| 228351_at | HEATR1 | -0.875 | 7.44 | 0.000005 | 0.00063 |
| 203036_s_at | MTSS1 | -0.879 | 6.95 | 0.000088 | 0.00279 |
| 1568720_at | ZNF506 | -0.879 | 5.34 | 0.000209 | 0.00469 |
| 214464_at | CDC42BPA | -0.879 | 6.90 | 0.000155 | 0.00390 |
| 224704_at | TNRC6A | -0.880 | 5.27 | 0.000461 | 0.00763 |
| 225465_at | MAGI1 | -0.881 | 6.15 | 0.000001 | 0.00031 |
| 1557675_at | RAF1 | -0.881 | 5.53 | 0.000206 | 0.00464 |
| 210962_s_at | AKAP9 | -0.881 | 7.19 | 0.000035 | 0.00166 |
| 218877_s_at | TRMT11 | -0.882 | 5.18 | 0.000567 | 0.00876 |
| 219163_at | ZNF562 | -0.885 | 6.57 | 0.000005 | 0.00058 |
| 235188_at | NA | -0.886 | 7.80 | 0.000116 | 0.00324 |
| 201446_s_at | TIA1 | -0.889 | 6.52 | 0.000633 | 0.00927 |
| 226075_at | SPSB1 | -0.890 | 6.41 | 0.000286 | 0.00571 |
| 214683_s_at | CLK1 | -0.890 | 6.44 | 0.000199 | 0.00458 |
| 225594_at | CREBZF | -0.891 | 6.86 | 0.000125 | 0.00337 |
| 201084_s_at | BCLAF1 | -0.894 | 7.22 | 0.000009 | 0.00087 |
| 1560803_at | DNAH3 | -0.895 | 7.95 | 0.000557 | 0.00866 |
| 208718_at | DDX17 | -0.895 | 9.32 | 0.000004 | 0.00050 |
| 1553162_x_at | PROSER3 | -0.895 | 6.91 | 0.000006 | 0.00068 |
| 222728_s_at | MIR1304 /// SNORA1 /// SNORA18 /// SNORA32 /// SNORA40 /// SNORA8 /// SNORD5 /// TAF1D | -0.896 | 7.64 | 0.000007 | 0.00071 |
| 213517_at | PCBP2 | -0.898 | 5.73 | 0.000125 | 0.00337 |
| 208151_x_at | DDX17 | -0.899 | 8.87 | 0.000002 | 0.00035 |
| 213074_at | PHIP | -0.899 | 7.01 | 0.000540 | 0.00852 |
| 210281_s_at | ZMYM2 | -0.901 | 6.43 | 0.000109 | 0.00312 |
| 224820_at | COX20 | -0.902 | 6.39 | 0.000051 | 0.00203 |
| 213049_at | RALGAPA1 | -0.903 | 6.17 | 0.000267 | 0.00546 |
| 234341_x_at | LOC91548 | -0.903 | 8.49 | 0.000121 | 0.00332 |
| 220452_x_at | NA | -0.904 | 7.92 | 0.000507 | 0.00814 |
| 224970_at | NFIA | -0.905 | 7.55 | 0.000016 | 0.00112 |
| 1557478_at | NA | -0.905 | 5.20 | 0.000222 | 0.00487 |
| 234997_x_at | RP11-488L18.10 | -0.906 | 6.77 | 0.000259 | 0.00536 |
| 212780_at | SOS1 | -0.906 | 5.37 | 0.000714 | 0.00995 |
| 1569723_a_at | SPIRE2 | -0.908 | 5.26 | 0.000391 | 0.00698 |
| 1554986_a_at | SNX19 | -0.908 | 6.40 | 0.000417 | 0.00719 |
| 213718_at | RBM14-RBM4 /// RBM4 | -0.909 | 6.31 | 0.000258 | 0.00535 |
| 218187_s_at | C8orf33 | -0.910 | 5.37 | 0.000407 | 0.00712 |
| 212339_at | EPB41L1 | -0.912 | 5.90 | 0.000372 | 0.00678 |
| 42361_g_at | CCHCR1 | -0.913 | 5.15 | 0.000049 | 0.00202 |
| 244881_at | LMLN | -0.913 | 6.57 | 0.000195 | 0.00451 |
| 226332_at | FAM133B /// FAM133DP | -0.913 | 5.99 | 0.000260 | 0.00536 |
| 230392_at | NA | -0.914 | 5.63 | 0.000455 | 0.00758 |
| 205317_s_at | SLC15A2 | -0.914 | 6.19 | 0.000351 | 0.00651 |
| 218269_at | DROSHA | -0.915 | 5.59 | 0.000014 | 0.00103 |
| 218882_s_at | WDR3 | -0.916 | 5.60 | 0.000546 | 0.00860 |
| 203384_s_at | GOLGA1 | -0.916 | 5.10 | 0.000001 | 0.00027 |
| 226917_s_at | ANAPC4 | -0.916 | 7.33 | 0.000625 | 0.00921 |
| 203804_s_at | LUC7L3 | -0.916 | 7.71 | 0.000457 | 0.00758 |
| 212666_at | SMURF1 | -0.916 | 5.26 | 0.000259 | 0.00536 |
| 203628_at | IGF1R | -0.917 | 5.72 | 0.000074 | 0.00255 |
| 214093_s_at | FUBP1 | -0.918 | 5.78 | 0.000321 | 0.00614 |
| 236346_at | NA | -0.918 | 5.31 | 0.000003 | 0.00046 |
| 222999_s_at | CCNL2 | -0.919 | 7.02 | 0.000076 | 0.00257 |
| 202814_s_at | HEXIM1 | -0.919 | 5.32 | 0.000173 | 0.00419 |
| 207564_x_at | OGT | -0.919 | 8.43 | 0.000020 | 0.00122 |
| 223724_s_at | STAG3L1 /// STAG3L2 /// STAG3L3 | -0.922 | 5.59 | 0.000052 | 0.00206 |
| 237251_at | LRRC71 | -0.922 | 6.85 | 0.000477 | 0.00781 |
| 239728_at | NA | -0.923 | 6.14 | 0.000013 | 0.00102 |
| 210892_s_at | GTF2I | -0.923 | 8.81 | 0.000000 | 0.00009 |
| 224565_at | MIR612 /// NEAT1 | -0.923 | 10.50 | 0.000029 | 0.00149 |
| 1567214_a_at | PNN | -0.925 | 6.90 | 0.000476 | 0.00781 |
| 215099_s_at | RXRB | -0.926 | 5.58 | 0.000206 | 0.00464 |
| 225127_at | TMEM181 | -0.927 | 5.50 | 0.000246 | 0.00521 |
| 225093_at | UTRN | -0.927 | 8.23 | 0.000001 | 0.00031 |
| 212068_s_at | PRRC2B | -0.928 | 6.87 | 0.000000 | 0.00004 |
| 217122_s_at | SLC35E2 /// SLC35E2B | -0.928 | 7.33 | 0.000007 | 0.00072 |
| 51228_at | RBM12B | -0.929 | 5.51 | 0.000553 | 0.00863 |
| 219543_at | PBLD | -0.930 | 5.86 | 0.000135 | 0.00357 |
| 200606_at | DSP | -0.931 | 8.48 | 0.000032 | 0.00158 |
| 212036_s_at | PNN | -0.933 | 7.25 | 0.000004 | 0.00058 |
| 226571_s_at | PTPRS | -0.934 | 7.29 | 0.000443 | 0.00743 |
| 212307_s_at | OGT | -0.934 | 6.75 | 0.000036 | 0.00166 |
| 218666_s_at | STX17 | -0.934 | 5.60 | 0.000037 | 0.00170 |
| 232230_at | LINC00263 | -0.934 | 5.21 | 0.000335 | 0.00633 |
| 229898_at | SNX33 | -0.937 | 5.65 | 0.000534 | 0.00845 |
| 225613_at | MAST4 | -0.937 | 6.82 | 0.000007 | 0.00070 |
| 228477_at | ARGLU1 | -0.938 | 8.74 | 0.000190 | 0.00444 |
| 1554824_at | ZNF585A | -0.938 | 5.62 | 0.000200 | 0.00458 |
| 204703_at | IFT88 | -0.939 | 7.21 | 0.000195 | 0.00451 |
| 231909_x_at | ODF2L | -0.941 | 5.41 | 0.000617 | 0.00915 |
| 210465_s_at | SNAPC3 | -0.944 | 5.58 | 0.000189 | 0.00444 |
| 242774_at | SYNE2 | -0.946 | 5.56 | 0.000570 | 0.00878 |
| 215002_at | LOC101929910 /// LOC613037 /// NPIPA5 /// NPIPB11 /// NPIPB3 /// NPIPB4 /// NPIPB5 /// NPIPB8 | -0.948 | 5.89 | 0.000065 | 0.00232 |
| 1554907_a_at | HYDIN /// HYDIN2 /// LOC101930373 | -0.949 | 5.16 | 0.000703 | 0.00985 |
| 228023_x_at | ACTG1P4 /// AMY2B /// RNPC3 | -0.949 | 5.69 | 0.000310 | 0.00598 |
| 221625_at | NA | -0.952 | 6.46 | 0.000003 | 0.00049 |
| 226297_at | HIPK3 | -0.953 | 7.27 | 0.000000 | 0.00014 |
| 202978_s_at | CREBZF | -0.957 | 6.02 | 0.000212 | 0.00472 |
| 208835_s_at | LUC7L3 | -0.957 | 7.84 | 0.000001 | 0.00027 |
| 211382_s_at | TACC2 | -0.959 | 7.14 | 0.000010 | 0.00091 |
| 1553793_a_at | KIAA1109 | -0.959 | 5.58 | 0.000005 | 0.00061 |
| 1564635_a_at | FHAD1 | -0.960 | 5.28 | 0.000438 | 0.00741 |
| 242712_x_at | RANBP2 /// RGPD1 /// RGPD2 /// RGPD3 /// RGPD4 /// RGPD5 /// RGPD6 /// RGPD8 | -0.962 | 5.13 | 0.000517 | 0.00827 |
| 231846_at | FOXRED2 | -0.963 | 5.63 | 0.000558 | 0.00866 |
| 223940_x_at | MALAT1 | -0.965 | 11.01 | 0.000002 | 0.00042 |
| 201928_at | PKP4 | -0.966 | 7.09 | 0.000001 | 0.00031 |
| 212107_s_at | DHX9 | -0.968 | 5.80 | 0.000002 | 0.00039 |
| 204883_s_at | HUS1 | -0.968 | 5.08 | 0.000396 | 0.00701 |
| 221833_at | LOC100507577 /// LONP2 /// SIAH1 | -0.969 | 5.29 | 0.000490 | 0.00794 |
| 243841_at | SYNE2 | -0.969 | 8.41 | 0.000030 | 0.00153 |
| 233132_at | BMS1P20 | -0.971 | 6.36 | 0.000440 | 0.00741 |
| 229663_at | LOC100507577 /// LONP2 | -0.971 | 5.34 | 0.000049 | 0.00202 |
| 216320_x_at | MST1 | -0.973 | 5.79 | 0.000092 | 0.00288 |
| 239082_at | FZD3 | -0.973 | 5.51 | 0.000261 | 0.00536 |
| 244828_x_at | NAF1 | -0.974 | 5.40 | 0.000182 | 0.00431 |
| 236222_at | MAATS1 | -0.974 | 8.60 | 0.000170 | 0.00413 |
| 224566_at | MIR612 /// NEAT1 | -0.975 | 10.49 | 0.000155 | 0.00390 |
| 213328_at | NEK1 | -0.975 | 6.73 | 0.000014 | 0.00104 |
| 244320_at | NHLRC2 | -0.975 | 6.25 | 0.000623 | 0.00920 |
| 231152_at | INO80D | -0.975 | 5.44 | 0.000043 | 0.00186 |
| 1553694_a_at | PIK3C2A | -0.976 | 6.17 | 0.000074 | 0.00255 |
| 228834_at | NA | -0.977 | 8.69 | 0.000017 | 0.00114 |
| 223097_at | ADPRHL2 | -0.979 | 7.87 | 0.000057 | 0.00217 |
| 240528_s_at | EXOC4 | -0.981 | 5.59 | 0.000147 | 0.00377 |
| 202432_at | PPP3CB | -0.981 | 5.36 | 0.000041 | 0.00178 |
| 224766_at | LOC100506548 /// RPL37 | -0.982 | 5.29 | 0.000052 | 0.00206 |
| 236620_at | LOC101929336 /// RIF1 | -0.982 | 5.80 | 0.000423 | 0.00724 |
| 211401_s_at | FGFR2 | -0.982 | 6.94 | 0.000118 | 0.00326 |
| 231875_at | KIF21A | -0.984 | 8.96 | 0.000376 | 0.00684 |
| 232386_at | VPS13C | -0.985 | 5.48 | 0.000018 | 0.00117 |
| 1554791_a_at | KANSL1L | -0.987 | 6.53 | 0.000562 | 0.00869 |
| 1560116_a_at | NEDD1 | -0.989 | 5.95 | 0.000329 | 0.00624 |
| 203827_at | WIPI1 | -0.990 | 7.48 | 0.000347 | 0.00648 |
| 224976_at | NFIA | -0.990 | 7.11 | 0.000069 | 0.00244 |
| 1553605_a_at | ABCA13 | -0.991 | 7.53 | 0.000128 | 0.00343 |
| 224429_x_at | NA | -0.994 | 7.01 | 0.000084 | 0.00274 |
| 211034_s_at | HECTD4 | -0.995 | 5.78 | 0.000012 | 0.00096 |
| 227227_at | NA | -0.995 | 7.15 | 0.000251 | 0.00528 |
| 212375_at | EP400 | -0.995 | 5.55 | 0.000021 | 0.00124 |
| 214250_at | NUMA1 | -0.996 | 6.43 | 0.000648 | 0.00933 |
| 203638_s_at | FGFR2 | -0.997 | 5.73 | 0.000140 | 0.00365 |
| 213353_at | ABCA5 | -0.997 | 6.35 | 0.000398 | 0.00703 |
| 214608_s_at | EYA1 | -0.999 | 6.00 | 0.000092 | 0.00288 |
| 221925_s_at | CSPP1 | -0.999 | 5.80 | 0.000200 | 0.00458 |
| 225256_at | CTC-444N24.11 | -1.000 | 6.26 | 0.000004 | 0.00050 |
| 1561079_at | ANKRD28 | -1.003 | 6.80 | 0.000194 | 0.00451 |
| 232244_at | KIAA1161 | -1.003 | 5.33 | 0.000000 | 0.00017 |
| 225570_at | SLC41A1 | -1.004 | 5.48 | 0.000108 | 0.00312 |
| 214222_at | DNAH7 | -1.004 | 6.66 | 0.000682 | 0.00965 |
| 243007_at | TTC5 | -1.006 | 5.82 | 0.000642 | 0.00929 |
| 214760_at | ZNF337 | -1.010 | 6.48 | 0.000351 | 0.00651 |
| 230333_at | NA | -1.010 | 8.86 | 0.000336 | 0.00635 |
| 228131_at | ERCC1 | -1.010 | 6.32 | 0.000123 | 0.00335 |
| 227484_at | SRGAP1 | -1.011 | 7.01 | 0.000094 | 0.00290 |
| 228719_at | ZSWIM7 | -1.012 | 6.69 | 0.000057 | 0.00217 |
| 229918_at | CCDC40 | -1.012 | 7.09 | 0.000103 | 0.00306 |
| 225726_s_at | PLEKHH1 | -1.013 | 7.69 | 0.000308 | 0.00597 |
| 229912_at | SDK1 | -1.013 | 7.11 | 0.000005 | 0.00063 |
| 210491_at | NA | -1.015 | 6.10 | 0.000457 | 0.00758 |
| 214890_s_at | FAM149A | -1.015 | 7.15 | 0.000040 | 0.00177 |
| 228077_at | MRI1 | -1.018 | 6.18 | 0.000012 | 0.00096 |
| 215111_s_at | TSC22D1 | -1.019 | 8.65 | 0.000078 | 0.00262 |
| 1560599_a_at | CEP89 | -1.020 | 6.26 | 0.000178 | 0.00425 |
| 212337_at | TUG1 | -1.022 | 6.69 | 0.000002 | 0.00040 |
| 243896_at | WDR96 | -1.023 | 8.47 | 0.000271 | 0.00550 |
| 204863_s_at | IL6ST | -1.025 | 6.73 | 0.000165 | 0.00409 |
| 242349_at | HECTD1 | -1.025 | 6.61 | 0.000031 | 0.00156 |
| 205464_at | SCNN1B | -1.030 | 6.31 | 0.000032 | 0.00158 |
| 235252_at | KSR1 | -1.030 | 6.36 | 0.000057 | 0.00217 |
| 226894_at | SLC35A3 | -1.031 | 6.36 | 0.000029 | 0.00146 |
| 229147_at | RASSF6 | -1.031 | 6.18 | 0.000304 | 0.00592 |
| 242142_at | NA | -1.031 | 5.23 | 0.000546 | 0.00860 |
| 215073_s_at | NR2F2 | -1.031 | 8.72 | 0.000018 | 0.00117 |
| 244571_s_at | TTC12 | -1.031 | 6.16 | 0.000412 | 0.00715 |
| 224686_x_at | LRRC37A2 | -1.031 | 6.59 | 0.000102 | 0.00304 |
| 240303_at | TMC5 | -1.032 | 8.96 | 0.000098 | 0.00299 |
| 215511_at | TCF20 | -1.034 | 5.46 | 0.000008 | 0.00075 |
| 206385_s_at | ANK3 | -1.035 | 8.24 | 0.000016 | 0.00114 |
| 214148_at | ITFG2 /// LOC100507424 | -1.035 | 5.15 | 0.000512 | 0.00821 |
| 220196_at | MUC16 | -1.036 | 8.45 | 0.000169 | 0.00413 |
| 242862_x_at | NA | -1.036 | 7.90 | 0.000112 | 0.00318 |
| 232527_at | PSMD6-AS2 | -1.038 | 5.53 | 0.000422 | 0.00724 |
| 214798_at | ATP2C2 | -1.039 | 6.89 | 0.000012 | 0.00096 |
| 225444_at | UBN2 | -1.039 | 5.71 | 0.000058 | 0.00218 |
| 238854_at | DYNLL1-AS1 | -1.039 | 6.20 | 0.000019 | 0.00120 |
| 211607_x_at | EGFR | -1.040 | 6.54 | 0.000625 | 0.00921 |
| 1553295_at | ABCA13 | -1.043 | 7.86 | 0.000002 | 0.00038 |
| 216908_x_at | RRN3P1 | -1.045 | 5.52 | 0.000099 | 0.00299 |
| 210544_s_at | ALDH3A2 | -1.045 | 7.52 | 0.000699 | 0.00982 |
| 233044_at | NA | -1.046 | 5.88 | 0.000048 | 0.00197 |
| 209290_s_at | NFIB | -1.046 | 7.08 | 0.000351 | 0.00651 |
| 1554726_at | ZNF655 | -1.047 | 6.18 | 0.000004 | 0.00056 |
| 221747_at | TNS1 | -1.047 | 6.39 | 0.000466 | 0.00769 |
| 1560089_at | LOC100289019 | -1.049 | 6.52 | 0.000015 | 0.00106 |
| 204028_s_at | RABGAP1 | -1.049 | 7.15 | 0.000001 | 0.00017 |
| 232283_at | LYSMD1 | -1.051 | 5.45 | 0.000010 | 0.00091 |
| 202054_s_at | ALDH3A2 | -1.052 | 7.63 | 0.000063 | 0.00229 |
| 235231_at | ZNF789 | -1.052 | 5.17 | 0.000363 | 0.00665 |
| 209119_x_at | NR2F2 | -1.053 | 7.94 | 0.000002 | 0.00039 |
| 214753_at | N4BP2L2 /// U50535 | -1.055 | 5.08 | 0.000483 | 0.00788 |
| 229951_x_at | NA | -1.056 | 6.21 | 0.000708 | 0.00989 |
| 231084_at | WDR96 | -1.058 | 8.89 | 0.000210 | 0.00469 |
| 233349_at | NA | -1.058 | 6.21 | 0.000003 | 0.00046 |
| 226110_at | PTAR1 | -1.058 | 5.82 | 0.000030 | 0.00153 |
| 1560081_at | RAD51-AS1 | -1.058 | 5.69 | 0.000183 | 0.00433 |
| 225759_x_at | CLMN | -1.060 | 7.33 | 0.000002 | 0.00042 |
| 231116_at | RP11-190A12.8 | -1.061 | 5.02 | 0.000170 | 0.00413 |
| 207495_at | RAB28 | -1.062 | 5.52 | 0.000003 | 0.00047 |
| 213653_at | METTL3 | -1.065 | 5.21 | 0.000387 | 0.00695 |
| 229053_at | SYT17 | -1.065 | 5.92 | 0.000643 | 0.00931 |
| 213649_at | SRSF7 | -1.066 | 6.52 | 0.000487 | 0.00791 |
| 213029_at | NFIB | -1.066 | 6.75 | 0.000187 | 0.00440 |
| 236717_at | FAM179A | -1.067 | 8.08 | 0.000641 | 0.00929 |
| 203651_at | ZFYVE16 | -1.068 | 6.01 | 0.000129 | 0.00345 |
| 229738_at | DNAH10 | -1.069 | 7.79 | 0.000682 | 0.00965 |
| 238032_at | NA | -1.071 | 5.07 | 0.000035 | 0.00166 |
| 214092_x_at | SUGP2 | -1.071 | 6.72 | 0.000000 | 0.00011 |
| 206993_at | ATP5S | -1.074 | 5.19 | 0.000085 | 0.00275 |
| 213380_x_at | LOC101930052 /// LOC102724562 /// MST1 /// MST1L /// MST1P2 | -1.074 | 5.43 | 0.000109 | 0.00312 |
| 218652_s_at | PIGG | -1.077 | 5.60 | 0.000018 | 0.00118 |
| 203431_s_at | ARHGAP32 | -1.080 | 6.66 | 0.000083 | 0.00273 |
| 215341_at | DNAH6 | -1.080 | 7.44 | 0.000026 | 0.00140 |
| 201450_s_at | TIA1 | -1.081 | 5.63 | 0.000007 | 0.00072 |
| 215388_s_at | CFH /// CFHR1 | -1.081 | 7.88 | 0.000202 | 0.00461 |
| 209497_s_at | RBM4B | -1.081 | 6.43 | 0.000088 | 0.00280 |
| 219886_at | CEP97 | -1.081 | 6.89 | 0.000423 | 0.00724 |
| 225805_at | HNRNPU | -1.082 | 5.55 | 0.000022 | 0.00127 |
| 1560910_at | PPIL6 | -1.082 | 5.67 | 0.000102 | 0.00304 |
| 228318_s_at | CRIPAK | -1.083 | 5.04 | 0.000239 | 0.00513 |
| 241747_s_at | CUL7 | -1.084 | 5.48 | 0.000281 | 0.00565 |
| 226877_at | RPL32P3 | -1.085 | 5.67 | 0.000604 | 0.00906 |
| 226764_at | ZNF827 | -1.088 | 5.22 | 0.000515 | 0.00824 |
| 220257_x_at | NXF2 /// NXF2B | -1.089 | 5.19 | 0.000050 | 0.00203 |
| 225825_at | C20orf194 | -1.090 | 5.87 | 0.000005 | 0.00063 |
| 1553314_a_at | KIF19 | -1.091 | 5.15 | 0.000058 | 0.00217 |
| 218926_at | MYNN | -1.092 | 5.34 | 0.000070 | 0.00246 |
| 244761_at | C5orf63 | -1.092 | 5.25 | 0.000118 | 0.00326 |
| 41660_at | CELSR1 | -1.093 | 7.94 | 0.000000 | 0.00012 |
| 219871_at | KLF3-AS1 | -1.094 | 5.13 | 0.000054 | 0.00212 |
| 221740_x_at | DQ597730 /// LRRC37A2 | -1.094 | 7.07 | 0.000390 | 0.00697 |
| 212361_s_at | ATP2A2 | -1.095 | 6.99 | 0.000255 | 0.00534 |
| 218067_s_at | ARGLU1 | -1.098 | 8.56 | 0.000010 | 0.00091 |
| 225054_x_at | LINC00674 | -1.103 | 6.32 | 0.000085 | 0.00275 |
| 238109_at | NA | -1.105 | 5.23 | 0.000548 | 0.00861 |
| 235190_at | NA | -1.106 | 5.31 | 0.000697 | 0.00981 |
| 1553440_at | AQP4-AS1 | -1.106 | 5.76 | 0.000077 | 0.00258 |
| 1558953_s_at | CEP164 | -1.107 | 7.04 | 0.000026 | 0.00139 |
| 1555568_at | GUSBP2 | -1.108 | 5.37 | 0.000336 | 0.00634 |
| 212232_at | FNBP4 | -1.109 | 6.12 | 0.000043 | 0.00186 |
| 201737_s_at | 38777 | -1.110 | 6.82 | 0.000000 | 0.00014 |
| 1555463_a_at | CHD6 | -1.110 | 6.00 | 0.000354 | 0.00654 |
| 230043_at | MUC20 | -1.111 | 6.10 | 0.000011 | 0.00091 |
| 239500_at | EFCAB1 | -1.113 | 5.61 | 0.000619 | 0.00916 |
| 226252_at | ZBTB20 | -1.114 | 6.04 | 0.000007 | 0.00071 |
| 1558768_at | DNAH1 | -1.115 | 6.14 | 0.000604 | 0.00906 |
| 212602_at | WDFY3 | -1.117 | 6.20 | 0.000010 | 0.00090 |
| 238584_at | IQCA1 | -1.118 | 7.19 | 0.000065 | 0.00232 |
| 228818_at | NA | -1.118 | 5.48 | 0.000188 | 0.00441 |
| 226999_at | RNPC3 | -1.118 | 5.30 | 0.000291 | 0.00578 |
| 205251_at | PER2 | -1.118 | 7.90 | 0.000007 | 0.00074 |
| 1555847_a_at | LOC284454 /// MIR23A /// MIR24-2 | -1.119 | 7.92 | 0.000319 | 0.00612 |
| 205408_at | MLLT10 | -1.120 | 6.28 | 0.000017 | 0.00116 |
| 1568763_s_at | LOC102724884 /// LOC728613 /// PDCD6 | -1.122 | 7.02 | 0.000140 | 0.00365 |
| 231989_s_at | LOC101060604 /// LOC101929910 /// LOC102725125 /// LOC613037 /// NPIPA5 /// NPIPB3 /// NPIPB4 /// NPIPB5 /// SLC7A5P1 /// SMG1P1 /// SMG1P3 | -1.123 | 7.27 | 0.000156 | 0.00390 |
| 220981_x_at | NXF2 /// NXF2B | -1.124 | 5.01 | 0.000383 | 0.00691 |
| 212362_at | ATP2A2 | -1.125 | 6.74 | 0.000001 | 0.00017 |
| 1555373_at | NA | -1.126 | 6.48 | 0.000051 | 0.00204 |
| 211596_s_at | LRIG1 | -1.127 | 7.80 | 0.000055 | 0.00214 |
| 1558686_at | MPV17L | -1.127 | 7.46 | 0.000113 | 0.00318 |
| 213865_at | DCBLD2 | -1.130 | 5.24 | 0.000352 | 0.00651 |
| 232994_s_at | ARHGEF28 | -1.130 | 6.47 | 0.000017 | 0.00114 |
| 234854_at | MDN1 | -1.132 | 5.96 | 0.000019 | 0.00120 |
| 228577_x_at | ODF2L | -1.136 | 6.75 | 0.000457 | 0.00758 |
| 232984_at | HYDIN /// HYDIN2 /// LOC101930373 | -1.137 | 7.56 | 0.000150 | 0.00381 |
| 201447_at | TIA1 | -1.137 | 5.34 | 0.000318 | 0.00611 |
| 223005_s_at | TMEM245 | -1.137 | 6.45 | 0.000008 | 0.00075 |
| 226450_at | INSR | -1.137 | 8.07 | 0.000001 | 0.00031 |
| 220344_at | C11orf16 | -1.138 | 7.17 | 0.000055 | 0.00214 |
| 1556567_at | NAP1L4 | -1.139 | 6.54 | 0.000113 | 0.00320 |
| 213839_at | CLMN | -1.139 | 7.35 | 0.000005 | 0.00062 |
| 239275_at | FRMPD2 /// FRMPD2P1 /// LOC101930006 | -1.139 | 5.28 | 0.000670 | 0.00957 |
| 242874_at | RP11-747H7.3 | -1.140 | 5.48 | 0.000305 | 0.00592 |
| 226575_at | ZNF462 | -1.143 | 5.31 | 0.000086 | 0.00277 |
| 1558154_at | AF289551 | -1.147 | 6.76 | 0.000261 | 0.00536 |
| 1558425_x_at | LINC00265 /// LOC101929038 | -1.148 | 6.85 | 0.000260 | 0.00536 |
| 226381_at | NA | -1.150 | 5.90 | 0.000088 | 0.00279 |
| 232525_at | NA | -1.151 | 5.16 | 0.000530 | 0.00841 |
| 218259_at | MKL2 | -1.154 | 6.98 | 0.000000 | 0.00016 |
| 223838_at | TSGA10 | -1.156 | 6.71 | 0.000124 | 0.00337 |
| 234991_at | ZXDC | -1.157 | 5.84 | 0.000075 | 0.00256 |
| 211086_x_at | NEK1 | -1.157 | 6.29 | 0.000025 | 0.00138 |
| 242800_at | NHS | -1.159 | 5.69 | 0.000450 | 0.00752 |
| 212079_s_at | KMT2A | -1.159 | 7.25 | 0.000020 | 0.00122 |
| 233893_s_at | UVSSA | -1.161 | 6.02 | 0.000030 | 0.00149 |
| 218768_at | NUP107 | -1.163 | 6.33 | 0.000018 | 0.00118 |
| 207950_s_at | ANK3 | -1.165 | 7.71 | 0.000000 | 0.00014 |
| 220917_s_at | WDR19 | -1.165 | 6.76 | 0.000292 | 0.00578 |
| 219497_s_at | BCL11A | -1.170 | 5.09 | 0.000241 | 0.00516 |
| 237383_at | NA | -1.171 | 5.20 | 0.000000 | 0.00008 |
| 239022_at | NA | -1.175 | 5.29 | 0.000242 | 0.00516 |
| 202053_s_at | ALDH3A2 | -1.176 | 7.08 | 0.000011 | 0.00092 |
| 214016_s_at | SFPQ | -1.177 | 8.48 | 0.000037 | 0.00169 |
| 233461_x_at | ZNF226 | -1.178 | 6.31 | 0.000001 | 0.00022 |
| 229966_at | EWSR1 | -1.179 | 6.01 | 0.000076 | 0.00257 |
| 228590_at | PTCD3 | -1.180 | 5.59 | 0.000639 | 0.00929 |
| 212176_at | PNISR | -1.181 | 7.07 | 0.000024 | 0.00135 |
| 228421_s_at | EFEMP1 | -1.181 | 7.76 | 0.000020 | 0.00123 |
| 1563830_a_at | FHAD1 | -1.186 | 5.72 | 0.000369 | 0.00671 |
| 242134_at | NA | -1.186 | 7.44 | 0.000036 | 0.00166 |
| 226182_s_at | WISP3 | -1.187 | 5.14 | 0.000003 | 0.00044 |
| 209185_s_at | IRS2 | -1.188 | 6.08 | 0.000588 | 0.00891 |
| 241402_at | TSEN54 | -1.188 | 5.91 | 0.000111 | 0.00318 |
| 238357_at | NA | -1.190 | 5.46 | 0.000217 | 0.00480 |
| 239785_at | DZIP1L | -1.190 | 7.37 | 0.000076 | 0.00257 |
| 232648_at | PSMA3 | -1.190 | 6.62 | 0.000276 | 0.00559 |
| 221556_at | CDC14B | -1.192 | 5.17 | 0.000310 | 0.00598 |
| 235547_at | N4BP2L2 | -1.193 | 6.67 | 0.000707 | 0.00989 |
| 213998_s_at | DDX17 | -1.197 | 9.66 | 0.000000 | 0.00012 |
| 215046_at | KANSL1L | -1.198 | 6.30 | 0.000059 | 0.00219 |
| 208518_s_at | PER2 | -1.200 | 6.83 | 0.000001 | 0.00018 |
| 236241_at | MED31 | -1.200 | 5.28 | 0.000104 | 0.00306 |
| 1565436_s_at | KMT2A | -1.201 | 6.94 | 0.000008 | 0.00080 |
| 209442_x_at | ANK3 | -1.201 | 7.70 | 0.000000 | 0.00011 |
| 226344_at | ZMAT1 | -1.202 | 5.30 | 0.000042 | 0.00180 |
| 223327_x_at | LOC80154 | -1.202 | 8.49 | 0.000414 | 0.00717 |
| 209006_s_at | LOC101928189 /// RSRP1 | -1.203 | 5.97 | 0.000064 | 0.00231 |
| 1570515_a_at | FILIP1 | -1.203 | 6.55 | 0.000117 | 0.00326 |
| 214294_at | KIAA0485 | -1.205 | 5.22 | 0.000003 | 0.00046 |
| 230500_at | PDE7A | -1.206 | 5.16 | 0.000029 | 0.00149 |
| 239069_s_at | NA | -1.206 | 7.01 | 0.000002 | 0.00040 |
| 239307_at | AF001548.5 | -1.210 | 5.58 | 0.000381 | 0.00690 |
| 219511_s_at | SNCAIP | -1.212 | 5.69 | 0.000019 | 0.00120 |
| 240183_at | TMEM213 | -1.212 | 6.61 | 0.000254 | 0.00533 |
| 1559021_at | C3orf52 | -1.212 | 5.35 | 0.000238 | 0.00513 |
| 214340_at | ALOX12P2 | -1.214 | 5.74 | 0.000636 | 0.00927 |
| 224901_at | SCD5 | -1.215 | 5.91 | 0.000636 | 0.00927 |
| 212179_at | PNISR | -1.216 | 6.75 | 0.000242 | 0.00517 |
| 211494_s_at | SLC4A4 | -1.216 | 7.13 | 0.000056 | 0.00215 |
| 228933_at | NHS | -1.218 | 5.84 | 0.000038 | 0.00171 |
| 214925_s_at | SPTAN1 | -1.221 | 5.98 | 0.000067 | 0.00237 |
| 231073_at | C1orf168 | -1.224 | 7.47 | 0.000501 | 0.00807 |
| 1557067_s_at | LUC7L | -1.224 | 5.92 | 0.000010 | 0.00087 |
| 230827_at | NA | -1.225 | 6.19 | 0.000002 | 0.00040 |
| 243729_at | RP11-747H7.3 | -1.225 | 6.13 | 0.000170 | 0.00413 |
| 230270_at | PRPF38B | -1.226 | 5.37 | 0.000114 | 0.00321 |
| 225180_at | TTC14 | -1.227 | 5.74 | 0.000035 | 0.00165 |
| 220108_at | GNA14 | -1.227 | 5.72 | 0.000036 | 0.00166 |
| 231315_at | NA | -1.228 | 6.16 | 0.000179 | 0.00426 |
| 214850_at | SMA4 | -1.233 | 6.15 | 0.000085 | 0.00275 |
| 223422_s_at | ARHGAP24 | -1.234 | 5.10 | 0.000039 | 0.00175 |
| 1554919_s_at | C7orf63 | -1.236 | 6.89 | 0.000326 | 0.00622 |
| 1554748_at | CLCNKB | -1.237 | 6.39 | 0.000256 | 0.00534 |
| 242337_at | NA | -1.238 | 6.20 | 0.000284 | 0.00569 |
| 230142_s_at | CIRBP | -1.239 | 5.50 | 0.000044 | 0.00188 |
| 239246_at | FARP1 | -1.239 | 5.59 | 0.000343 | 0.00645 |
| 228111_s_at | DNAH1 | -1.241 | 7.92 | 0.000013 | 0.00102 |
| 1561430_s_at | MAATS1 | -1.242 | 7.44 | 0.000123 | 0.00335 |
| 238895_at | NA | -1.243 | 5.37 | 0.000001 | 0.00024 |
| 229712_at | SNAPC3 | -1.244 | 5.50 | 0.000058 | 0.00218 |
| 242647_at | USP34 | -1.246 | 6.32 | 0.000003 | 0.00046 |
| 209487_at | RBPMS | -1.249 | 7.62 | 0.000000 | 0.00008 |
| 229407_at | SDK1 | -1.249 | 5.75 | 0.000059 | 0.00220 |
| 205316_at | SLC15A2 | -1.251 | 7.90 | 0.000024 | 0.00136 |
| 221264_s_at | TARDBP | -1.256 | 5.03 | 0.000097 | 0.00297 |
| 242476_at | NA | -1.257 | 5.42 | 0.000648 | 0.00933 |
| 1569974_x_at | BC015774 /// SEPT7P2 | -1.259 | 5.28 | 0.000002 | 0.00040 |
| 225786_at | HNRNPU-AS1 | -1.259 | 6.19 | 0.000027 | 0.00141 |
| 230403_at | RFX3 | -1.260 | 8.22 | 0.000057 | 0.00217 |
| 237227_at | NEK10 | -1.263 | 7.47 | 0.000016 | 0.00111 |
| 227809_at | ZC3H6 | -1.264 | 6.06 | 0.000000 | 0.00017 |
| 243937_x_at | AGAP6 | -1.265 | 6.37 | 0.000020 | 0.00121 |
| 1554512_a_at | CEP89 | -1.268 | 6.21 | 0.000071 | 0.00249 |
| 231291_at | GIPR | -1.280 | 7.75 | 0.000091 | 0.00286 |
| 1558854_a_at | NA | -1.280 | 5.29 | 0.000093 | 0.00289 |
| 240145_at | DGKH | -1.281 | 6.83 | 0.000099 | 0.00299 |
| 229413_s_at | NA | -1.283 | 5.73 | 0.000001 | 0.00018 |
| 227105_at | CSPP1 | -1.285 | 5.94 | 0.000290 | 0.00577 |
| 220187_at | STEAP4 | -1.288 | 6.37 | 0.000582 | 0.00887 |
| 220713_at | DENND6B | -1.288 | 5.13 | 0.000137 | 0.00362 |
| 227415_at | DGKH | -1.289 | 6.96 | 0.000052 | 0.00206 |
| 1570202_a_at | MKL2 | -1.294 | 7.80 | 0.000000 | 0.00004 |
| 233375_at | EFCAB2 | -1.295 | 6.53 | 0.000557 | 0.00866 |
| 235274_at | GABPB1-AS1 | -1.297 | 6.19 | 0.000296 | 0.00584 |
| 228811_at | NA | -1.297 | 6.32 | 0.000007 | 0.00071 |
| 207330_at | PZP | -1.301 | 5.82 | 0.000461 | 0.00763 |
| 211220_s_at | HSF2 | -1.302 | 5.94 | 0.000050 | 0.00203 |
| 220952_s_at | PLEKHA5 | -1.302 | 6.91 | 0.000002 | 0.00039 |
| 1561939_at | DYNC2H1 | -1.302 | 6.35 | 0.000432 | 0.00734 |
| 229111_at | RP4-635E18.8 | -1.304 | 5.24 | 0.000137 | 0.00362 |
| 241397_at | NA | -1.305 | 5.40 | 0.000561 | 0.00868 |
| 235080_at | CLUAP1 | -1.308 | 5.74 | 0.000594 | 0.00896 |
| 235616_at | TSHZ2 | -1.310 | 5.69 | 0.000002 | 0.00033 |
| 227819_at | LGR6 | -1.311 | 5.59 | 0.000654 | 0.00937 |
| 1554708_s_at | SPATA6L | -1.314 | 7.07 | 0.000180 | 0.00428 |
| 237746_at | NA | -1.315 | 5.15 | 0.000173 | 0.00419 |
| 201984_s_at | EGFR | -1.317 | 7.55 | 0.000023 | 0.00130 |
| 240230_s_at | AGAP9 | -1.317 | 5.91 | 0.000115 | 0.00323 |
| 1559105_at | LEKR1 | -1.318 | 5.49 | 0.000423 | 0.00724 |
| 1560001_at | LOC100131581 | -1.318 | 6.61 | 0.000228 | 0.00498 |
| 1557636_a_at | C7orf57 | -1.319 | 6.85 | 0.000407 | 0.00713 |
| 1565621_at | NA | -1.322 | 5.55 | 0.000441 | 0.00741 |
| 214157_at | GNAS | -1.322 | 6.29 | 0.000074 | 0.00255 |
| 223007_s_at | TMEM245 | -1.323 | 5.67 | 0.000002 | 0.00040 |
| 209561_at | THBS3 | -1.323 | 5.73 | 0.000010 | 0.00091 |
| 228466_at | GABPB2 | -1.324 | 6.07 | 0.000016 | 0.00112 |
| 221103_s_at | WDR52 | -1.326 | 7.15 | 0.000025 | 0.00137 |
| 244331_at | NA | -1.332 | 5.89 | 0.000032 | 0.00158 |
| 1557034_s_at | LOC100134445 /// LOC100288778 /// LOC100653296 /// WASH1 /// WASH2P /// WASH3P /// WASH5P /// WASH7P | -1.333 | 7.11 | 0.000054 | 0.00212 |
| 227260_at | NA | -1.333 | 7.87 | 0.000005 | 0.00062 |
| 1566505_at | ERVK13-1 | -1.336 | 5.72 | 0.000026 | 0.00139 |
| 213472_at | HNRNPH1 | -1.337 | 6.33 | 0.000025 | 0.00137 |
| 238467_at | DYNLL2 | -1.337 | 5.02 | 0.000021 | 0.00124 |
| 242615_at | CCDC37 | -1.339 | 5.94 | 0.000018 | 0.00118 |
| 239828_at | AK9 | -1.340 | 5.45 | 0.000027 | 0.00141 |
| 209184_s_at | IRS2 | -1.342 | 6.86 | 0.000149 | 0.00380 |
| 244478_at | LRRC37A3 | -1.343 | 5.50 | 0.000022 | 0.00127 |
| 1562462_at | DNAH10 | -1.343 | 6.22 | 0.000008 | 0.00074 |
| 230561_s_at | KANSL1L | -1.346 | 5.29 | 0.000087 | 0.00278 |
| 1561207_at | RP11-53B2.2 | -1.350 | 5.36 | 0.000366 | 0.00669 |
| 1553604_at | ABCA13 | -1.351 | 7.59 | 0.000025 | 0.00138 |
| 210800_at | TIMM8A | -1.352 | 6.39 | 0.000013 | 0.00102 |
| 236015_at | NA | -1.353 | 5.08 | 0.000018 | 0.00117 |
| 244748_at | NA | -1.354 | 5.94 | 0.000058 | 0.00218 |
| 235412_at | ARHGEF7 | -1.354 | 5.18 | 0.000411 | 0.00715 |
| 214712_at | SNX29P2 | -1.354 | 5.82 | 0.000005 | 0.00058 |
| 209170_s_at | GPM6B | -1.355 | 6.10 | 0.000041 | 0.00178 |
| 1552299_at | AK9 | -1.355 | 6.99 | 0.000203 | 0.00461 |
| 209936_at | RBM5 | -1.356 | 5.69 | 0.000500 | 0.00805 |
| 238983_at | NSUN7 | -1.357 | 7.08 | 0.000063 | 0.00227 |
| 215921_at | NPIPA5 /// NPIPB3 /// NPIPB6 /// NPIPB8 | -1.357 | 6.86 | 0.000079 | 0.00263 |
| 205073_at | CYP2J2 | -1.358 | 6.18 | 0.000001 | 0.00031 |
| 238924_at | BMS1P5 | -1.359 | 5.21 | 0.000175 | 0.00420 |
| 235606_at | LINC00883 | -1.360 | 6.59 | 0.000122 | 0.00335 |
| 232964_at | SPDYE1 /// SPDYE2 /// SPDYE2B /// SPDYE5 /// SPDYE6 | -1.362 | 7.76 | 0.000245 | 0.00521 |
| 233839_at | EFCAB6 | -1.362 | 7.30 | 0.000013 | 0.00102 |
| 228287_at | ING5 | -1.365 | 5.36 | 0.000041 | 0.00180 |
| 227432_s_at | NA | -1.365 | 9.01 | 0.000000 | 0.00008 |
| 225191_at | CIRBP | -1.366 | 6.71 | 0.000010 | 0.00091 |
| 236173_s_at | LRIG1 | -1.368 | 6.76 | 0.000000 | 0.00003 |
| 229325_at | ZZZ3 | -1.368 | 5.40 | 0.000000 | 0.00017 |
| 228528_at | MIR29B2 /// MIR29C | -1.370 | 5.92 | 0.000018 | 0.00118 |
| 238339_x_at | LRIG1 | -1.372 | 7.28 | 0.000000 | 0.00010 |
| 229465_s_at | NA | -1.373 | 7.70 | 0.000110 | 0.00314 |
| 238408_at | NA | -1.374 | 6.80 | 0.000384 | 0.00693 |
| 234681_s_at | CHD6 | -1.375 | 5.81 | 0.000002 | 0.00033 |
| 1556035_s_at | ZNF207 | -1.376 | 5.52 | 0.000575 | 0.00883 |
| 234006_s_at | TMEM234 | -1.381 | 5.96 | 0.000000 | 0.00004 |
| 239432_at | FLJ31306 | -1.382 | 5.94 | 0.000154 | 0.00389 |
| 208360_s_at | ERVH-4 | -1.384 | 5.75 | 0.000260 | 0.00536 |
| 1552532_a_at | ATP6V1C2 | -1.385 | 5.92 | 0.000522 | 0.00831 |
| 226419_s_at | SRSF1 | -1.389 | 6.26 | 0.000206 | 0.00464 |
| 204591_at | CHL1 | -1.392 | 7.94 | 0.000003 | 0.00046 |
| 204379_s_at | FGFR3 | -1.394 | 7.50 | 0.000013 | 0.00102 |
| 236740_at | NA | -1.397 | 5.38 | 0.000035 | 0.00166 |
| 217554_at | NA | -1.399 | 5.38 | 0.000146 | 0.00377 |
| 216000_at | NA | -1.400 | 7.30 | 0.000107 | 0.00309 |
| 229497_at | ANKDD1A | -1.401 | 5.77 | 0.000001 | 0.00031 |
| 1555749_at | SF1 | -1.402 | 5.13 | 0.000193 | 0.00450 |
| 241310_at | NEK5 | -1.402 | 6.88 | 0.000039 | 0.00177 |
| 214073_at | CTTN | -1.402 | 5.80 | 0.000001 | 0.00029 |
| 235511_at | NA | -1.403 | 6.06 | 0.000042 | 0.00180 |
| 238631_at | NA | -1.403 | 5.27 | 0.000034 | 0.00163 |
| 218918_at | MAN1C1 | -1.405 | 6.06 | 0.000017 | 0.00116 |
| 242080_at | NA | -1.406 | 5.61 | 0.000572 | 0.00880 |
| 227939_s_at | TRA2A | -1.407 | 5.27 | 0.000132 | 0.00353 |
| 238658_at | NA | -1.409 | 5.27 | 0.000042 | 0.00180 |
| 234168_at | NA | -1.411 | 5.02 | 0.000145 | 0.00375 |
| 237395_at | CYP4Z1 | -1.415 | 5.62 | 0.000093 | 0.00289 |
| 213792_s_at | INSR | -1.416 | 8.51 | 0.000000 | 0.00008 |
| 1555942_a_at | MIR205 | -1.418 | 6.57 | 0.000311 | 0.00600 |
| 242812_at | HCG18 | -1.419 | 5.72 | 0.000192 | 0.00450 |
| 213742_at | SRSF11 | -1.420 | 5.02 | 0.000015 | 0.00109 |
| 235167_at | LOC100190986 | -1.422 | 5.80 | 0.000015 | 0.00106 |
| 226975_at | RNPC3 | -1.422 | 6.52 | 0.000005 | 0.00060 |
| 243938_x_at | DNAH5 | -1.423 | 8.49 | 0.000083 | 0.00272 |
| 224276_at | ZNF33A | -1.424 | 5.05 | 0.000309 | 0.00598 |
| 232615_at | NA | -1.426 | 6.30 | 0.000018 | 0.00116 |
| 205997_at | ADAM28 | -1.427 | 7.26 | 0.000011 | 0.00095 |
| 1570314_at | NA | -1.428 | 5.08 | 0.000059 | 0.00219 |
| 1553159_at | DNAH11 | -1.428 | 7.49 | 0.000202 | 0.00461 |
| 236215_at | NA | -1.437 | 5.26 | 0.000446 | 0.00746 |
| 229388_at | LOC101929112 | -1.438 | 8.03 | 0.000203 | 0.00461 |
| 238239_at | WDR27 | -1.443 | 5.07 | 0.000025 | 0.00138 |
| 1565338_x_at | DNAH6 | -1.447 | 5.96 | 0.000012 | 0.00096 |
| 208078_s_at | SIK1 | -1.449 | 7.37 | 0.000364 | 0.00667 |
| 213593_s_at | TRA2A | -1.449 | 5.07 | 0.000009 | 0.00081 |
| 221973_at | LOC100506076 /// LOC100506123 | -1.449 | 6.62 | 0.000005 | 0.00063 |
| 1559313_at | DQ573539 /// SNX18P3 /// SNX18P3 | -1.450 | 5.76 | 0.000138 | 0.00364 |
| 1557867_s_at | C9orf117 | -1.451 | 9.37 | 0.000087 | 0.00279 |
| 231127_at | NA | -1.453 | 5.19 | 0.000125 | 0.00337 |
| 207402_at | ZNF132 | -1.453 | 5.27 | 0.000166 | 0.00410 |
| 235060_at | LOC100190986 | -1.455 | 6.73 | 0.000628 | 0.00923 |
| 212454_x_at | HNRNPDL | -1.455 | 6.07 | 0.000000 | 0.00004 |
| 226404_at | RBM39 | -1.462 | 6.45 | 0.000002 | 0.00040 |
| 226412_at | PNISR | -1.462 | 5.28 | 0.000000 | 0.00014 |
| 1558423_at | LINC00265 /// LOC101929038 | -1.465 | 6.73 | 0.000277 | 0.00561 |
| 229169_at | TTC18 | -1.469 | 6.97 | 0.000300 | 0.00589 |
| 228919_at | NA | -1.478 | 7.43 | 0.000057 | 0.00217 |
| 226363_at | ABCC5 | -1.481 | 6.11 | 0.000000 | 0.00017 |
| 233516_s_at | SPAG17 | -1.485 | 7.14 | 0.000257 | 0.00535 |
| 226908_at | LRIG3 | -1.488 | 5.84 | 0.000007 | 0.00071 |
| 1565337_at | DNAH6 | -1.489 | 6.16 | 0.000036 | 0.00166 |
| 224209_s_at | GDA | -1.489 | 6.31 | 0.000139 | 0.00364 |
| 1560353_at | NA | -1.490 | 6.04 | 0.000067 | 0.00237 |
| 233265_at | NA | -1.493 | 5.44 | 0.000006 | 0.00066 |
| 224517_at | POLR2J4 | -1.495 | 6.49 | 0.000095 | 0.00291 |
| 211310_at | EZH1 | -1.496 | 5.11 | 0.000009 | 0.00084 |
| 220919_s_at | WDR96 | -1.496 | 6.98 | 0.000020 | 0.00122 |
| 220092_s_at | ANTXR1 | -1.497 | 5.85 | 0.000200 | 0.00458 |
| 226216_at | INSR | -1.501 | 7.05 | 0.000000 | 0.00004 |
| 244786_at | SCARNA13 /// SNHG10 | -1.501 | 5.01 | 0.000053 | 0.00209 |
| 242343_x_at | NA | -1.502 | 6.14 | 0.000289 | 0.00576 |
| 1560119_at | LINC00937 | -1.503 | 6.15 | 0.000345 | 0.00645 |
| 232381_s_at | DNAH5 | -1.506 | 8.50 | 0.000013 | 0.00102 |
| 232360_at | EHF | -1.506 | 5.32 | 0.000016 | 0.00112 |
| 1556126_s_at | NA | -1.508 | 5.30 | 0.000219 | 0.00483 |
| 214745_at | PLCH1 | -1.509 | 5.47 | 0.000009 | 0.00084 |
| 210172_at | SF1 | -1.512 | 6.13 | 0.000005 | 0.00063 |
| 235868_at | MGEA5 | -1.516 | 6.15 | 0.000102 | 0.00304 |
| 238409_x_at | OXR1 | -1.517 | 6.41 | 0.000156 | 0.00390 |
| 1557383_a_at | RP5-1092A3.4 | -1.523 | 7.10 | 0.000236 | 0.00509 |
| 229177_at | C16orf89 | -1.523 | 7.35 | 0.000381 | 0.00690 |
| 210365_at | LOC100506403 /// LOC101928269 /// RUNX1 | -1.528 | 5.76 | 0.000075 | 0.00256 |
| 1569157_s_at | LOC100505555 /// ZNF846 | -1.532 | 5.14 | 0.000367 | 0.00669 |
| 234893_s_at | DNAH6 | -1.541 | 5.46 | 0.000188 | 0.00441 |
| 225474_at | MAGI1 | -1.541 | 5.80 | 0.000020 | 0.00123 |
| 237804_at | DNAH11 | -1.547 | 7.73 | 0.000047 | 0.00195 |
| 219389_at | SUSD4 | -1.551 | 5.76 | 0.000001 | 0.00027 |
| 241509_at | NA | -1.555 | 6.53 | 0.000228 | 0.00498 |
| 219813_at | LATS1 | -1.559 | 6.03 | 0.000001 | 0.00019 |
| 238341_at | NA | -1.563 | 5.43 | 0.000051 | 0.00204 |
| 232449_at | BCO2 | -1.575 | 6.45 | 0.000000 | 0.00017 |
| 226848_at | NA | -1.582 | 5.86 | 0.000083 | 0.00272 |
| 225178_at | TTC14 | -1.583 | 6.69 | 0.000000 | 0.00008 |
| 215904_at | MLLT4 | -1.587 | 6.95 | 0.000174 | 0.00419 |
| 230152_at | WDR52 | -1.594 | 6.24 | 0.000005 | 0.00060 |
| 81737_at | LOC100505915 | -1.596 | 6.89 | 0.000000 | 0.00017 |
| 206318_at | EPPIN /// EPPIN-WFDC6 | -1.597 | 6.15 | 0.000000 | 0.00007 |
| 1559812_at | FAM53B-AS1 | -1.600 | 5.29 | 0.000484 | 0.00788 |
| 205583_s_at | ALG13 | -1.602 | 5.25 | 0.000002 | 0.00038 |
| 237910_x_at | FAM92A1 | -1.605 | 6.04 | 0.000003 | 0.00047 |
| 228370_at | IPW /// LOC101930404 /// PWARSN /// SNORD107 /// SNORD115-13 /// SNORD115-26 /// SNORD115-7 /// SNORD116-22 /// SNORD116-28 /// SNORD116-4 | -1.608 | 5.05 | 0.000598 | 0.00900 |
| 219153_s_at | THSD4 | -1.608 | 5.29 | 0.000522 | 0.00831 |
| 234727_at | DNAH7 | -1.609 | 6.42 | 0.000003 | 0.00046 |
| 216291_at | ZNF440 | -1.610 | 5.99 | 0.000067 | 0.00236 |
| 213169_at | SEMA5A | -1.610 | 5.77 | 0.000486 | 0.00790 |
| 213784_at | IFT27 | -1.611 | 5.72 | 0.000008 | 0.00080 |
| 236542_at | NA | -1.612 | 6.24 | 0.000015 | 0.00106 |
| 60815_at | POLR2J4 | -1.617 | 8.10 | 0.000029 | 0.00149 |
| 241436_at | SCNN1G | -1.628 | 5.06 | 0.000292 | 0.00578 |
| 211834_s_at | TP63 | -1.636 | 6.27 | 0.000589 | 0.00893 |
| 1554256_a_at | PCNXL2 | -1.636 | 5.08 | 0.000034 | 0.00162 |
| 214341_at | AP1G2 | -1.637 | 5.37 | 0.000008 | 0.00078 |
| 237483_at | NA | -1.640 | 6.10 | 0.000125 | 0.00337 |
| 1560426_at | C12orf55 | -1.641 | 6.50 | 0.000103 | 0.00306 |
| 223679_at | CTNNB1 | -1.643 | 5.17 | 0.000013 | 0.00101 |
| 238342_at | NA | -1.646 | 5.70 | 0.000009 | 0.00086 |
| 223824_at | RNLS | -1.647 | 6.27 | 0.000549 | 0.00862 |
| 228030_at | RBM6 | -1.652 | 6.13 | 0.000000 | 0.00002 |
| 230180_at | DDX17 | -1.656 | 8.27 | 0.000000 | 0.00011 |
| 212977_at | ACKR3 | -1.657 | 7.14 | 0.000001 | 0.00031 |
| 224489_at | LOC100294341 /// LOC101929774 | -1.662 | 5.52 | 0.000702 | 0.00984 |
| 220854_at | NA | -1.668 | 5.01 | 0.000005 | 0.00058 |
| 220064_at | LOC100506124 /// TTC21B | -1.671 | 5.99 | 0.000170 | 0.00414 |
| 1563958_at | NA | -1.683 | 5.74 | 0.000000 | 0.00009 |
| 1563097_at | DNAH12 | -1.686 | 7.39 | 0.000025 | 0.00137 |
| 220564_at | RNLS | -1.698 | 6.27 | 0.000455 | 0.00758 |
| 244297_at | ANKRD18A | -1.704 | 5.48 | 0.000116 | 0.00324 |
| 1552343_s_at | PDE7A | -1.704 | 5.20 | 0.000001 | 0.00027 |
| 241635_at | NA | -1.707 | 5.25 | 0.000026 | 0.00140 |
| 1555997_s_at | IGFBP5 | -1.710 | 6.24 | 0.000486 | 0.00790 |
| 1559410_at | NA | -1.711 | 6.37 | 0.000006 | 0.00067 |
| 228640_at | PCDH7 | -1.718 | 5.72 | 0.000018 | 0.00117 |
| 239151_at | BMS1P5 | -1.729 | 7.28 | 0.000244 | 0.00519 |
| 236000_s_at | NA | -1.736 | 5.80 | 0.000000 | 0.00006 |
| 237912_at | NA | -1.741 | 7.08 | 0.000001 | 0.00027 |
| 241614_at | RP11-471B22.2 | -1.741 | 5.62 | 0.000011 | 0.00091 |
| 240547_at | NA | -1.746 | 5.24 | 0.000080 | 0.00266 |
| 225656_at | EFHC1 | -1.751 | 6.76 | 0.000089 | 0.00282 |
| 1563259_at | NA | -1.754 | 5.68 | 0.000005 | 0.00061 |
| 234759_at | LOC100287497 | -1.762 | 5.35 | 0.000003 | 0.00047 |
| 1557066_at | LUC7L | -1.769 | 5.82 | 0.000000 | 0.00016 |
| 233623_at | NA | -1.785 | 5.63 | 0.000001 | 0.00022 |
| 214349_at | NA | -1.786 | 7.74 | 0.000176 | 0.00421 |
| 237086_at | FOXA1 | -1.788 | 5.37 | 0.000008 | 0.00075 |
| 241091_at | AGO3 | -1.790 | 5.51 | 0.000076 | 0.00257 |
| 241797_at | NA | -1.791 | 5.24 | 0.000072 | 0.00249 |
| 222368_at | NA | -1.793 | 7.99 | 0.000239 | 0.00513 |
| 234476_at | DNAH7 | -1.803 | 7.26 | 0.000000 | 0.00010 |
| 237527_at | NA | -1.807 | 8.08 | 0.000014 | 0.00103 |
| 231687_at | ERICH3-AS1 | -1.813 | 5.03 | 0.000006 | 0.00065 |
| 241193_at | ETS2 | -1.818 | 6.02 | 0.000271 | 0.00550 |
| 238553_at | AGAP6 /// AGAP9 /// BMS1P5 | -1.819 | 6.20 | 0.000001 | 0.00018 |
| 228613_at | RAB11FIP3 | -1.825 | 6.31 | 0.000014 | 0.00106 |
| 1566265_at | NA | -1.825 | 5.26 | 0.000054 | 0.00211 |
| 1566502_at | NA | -1.833 | 5.88 | 0.000049 | 0.00200 |
| 1557866_at | C9orf117 | -1.840 | 7.26 | 0.000011 | 0.00092 |
| 1568836_at | CLK4 | -1.858 | 6.19 | 0.000092 | 0.00288 |
| 1558220_at | NA | -1.861 | 5.72 | 0.000025 | 0.00138 |
| 230064_at | NA | -1.863 | 6.22 | 0.000136 | 0.00360 |
| 244162_at | NA | -1.873 | 6.06 | 0.000033 | 0.00162 |
| 233198_at | GOLGA2P5 | -1.889 | 5.96 | 0.000000 | 0.00017 |
| 227702_at | CYP4X1 | -1.889 | 6.11 | 0.000012 | 0.00096 |
| 1558075_at | NA | -1.890 | 8.27 | 0.000000 | 0.00004 |
| 1559890_a_at | ABI1 | -1.890 | 5.29 | 0.000007 | 0.00071 |
| 238625_at | C1orf168 | -1.898 | 6.24 | 0.000142 | 0.00369 |
| 241628_at | NA | -1.901 | 5.12 | 0.000058 | 0.00218 |
| 1559950_at | FAM66B /// FAM66C /// FAM66D /// LOC101928910 /// LOC102725108 | -1.911 | 5.84 | 0.000002 | 0.00038 |
| 242868_at | NA | -1.915 | 6.87 | 0.000003 | 0.00045 |
| 213488_at | SNED1 | -1.939 | 5.19 | 0.000003 | 0.00045 |
| 204734_at | KRT15 | -1.954 | 7.82 | 0.000010 | 0.00088 |
| 242393_x_at | AGAP6 | -1.957 | 7.50 | 0.000000 | 0.00008 |
| 216415_at | DNAH3 | -1.981 | 5.04 | 0.000006 | 0.00068 |
| 225727_at | PLEKHH1 | -1.985 | 5.61 | 0.000046 | 0.00192 |
| 207636_at | SERPINI2 | -1.987 | 5.62 | 0.000001 | 0.00027 |
| 232603_at | DCDC1 /// DCDC5 | -1.999 | 5.08 | 0.000004 | 0.00053 |
| 229427_at | SEMA5A | -2.022 | 5.68 | 0.000055 | 0.00214 |
| 1553211_at | ANKFN1 | -2.022 | 6.54 | 0.000387 | 0.00695 |
| 237220_at | NA | -2.023 | 6.06 | 0.000383 | 0.00691 |
| 1552463_at | SERPINB11 | -2.028 | 6.91 | 0.000009 | 0.00084 |
| 1558937_s_at | NA | -2.029 | 5.38 | 0.000003 | 0.00046 |
| 1557270_at | NA | -2.041 | 5.36 | 0.000002 | 0.00034 |
| 222145_at | NA | -2.047 | 6.22 | 0.000000 | 0.00003 |
| 231106_at | BMS1P6 | -2.060 | 5.93 | 0.000001 | 0.00030 |
| 242121_at | NA | -2.083 | 5.66 | 0.000147 | 0.00377 |
| 1553318_at | RIBC1 | -2.084 | 6.35 | 0.000023 | 0.00131 |
| 240156_at | NA | -2.086 | 5.16 | 0.000002 | 0.00038 |
| 241673_x_at | NA | -2.135 | 5.42 | 0.000017 | 0.00116 |
| 213790_at | ADAM12 | -2.191 | 5.88 | 0.000007 | 0.00072 |
| 244635_s_at | NA | -2.196 | 5.95 | 0.000034 | 0.00164 |
| 1562562_at | ANKUB1 | -2.202 | 5.44 | 0.000355 | 0.00655 |
| 222940_at | SULT1E1 | -2.207 | 5.44 | 0.000188 | 0.00441 |
| 222835_at | THSD4 | -2.288 | 5.98 | 0.000004 | 0.00057 |
| 230378_at | SCGB3A1 | -2.438 | 11.50 | 0.000011 | 0.00091 |
| 210297_s_at | MSMB | -2.447 | 10.25 | 0.000009 | 0.00086 |
| 213432_at | MUC5B | -2.457 | 5.25 | 0.000002 | 0.00040 |
| 1556735_at | NA | -2.467 | 5.84 | 0.000001 | 0.00020 |
| 1558777_at | MKL2 | -2.497 | 5.48 | 0.000006 | 0.00068 |
| 238371_s_at | NA | -2.564 | 5.38 | 0.000000 | 0.00009 |
| 241676_x_at | NA | -2.723 | 5.19 | 0.000013 | 0.00101 |
| 222268_x_at | MUC5B | -2.737 | 10.92 | 0.000000 | 0.00008 |
| 238372_s_at | NA | -2.860 | 5.00 | 0.000000 | 0.00004 |
| 207430_s_at | MSMB | -2.899 | 10.72 | 0.000001 | 0.00032 |
