# Supplementary material for: Blood eosinophil count and airway epithelial transcriptome relationships in COPD versus asthma
Source: Allergy. 2019 Sep 10;75(2):370–80. doi: 10.1111/all.14016 (PMC7064968; doi:10.1111/all.14016)
Supplement: Supplementary file 4 [file ALL-75-370-s004.docx]

**Relationship between blood eosinophil count and bronchial epithelial gene expression in COPD versus Asthma- discordance between ‘treatable trait’ and molecular signatures**

Leena George^1^*, Adam R Taylor^2^*, Anna Esteve-Codina^3^*, María Soler Artigas^1,3,4^, Gian Andri Thun^3^, Stewart Bates^2^, Stelios Pavlidis^5,6^, Scott Wagers^7^, Anne Boland^8^, Antje Prasse^9^, Piera Boschetto^10^, David G Parr^11^, Adam Nowinski^12^, Imre Barta^13^, Jens Hohlfeld^14^, Timm Greulich^15^, Maarten van den Berge^16^, Pieter S Hiemstra^17^, Wim Timens^18^, Timothy Hinks^19^, Sally Wenzel^20^, Salman Siddiqui^1^, Matthew Richardson^1^, Per Venge^21^, Simon Heath^3^, Ivo Gut^3,22^, Martin D Tobin^1^, Lindsay Edwards^2^, John H Riley^2^, Ratko Djukanovic^23^, Charles Auffray^24^, Bertrand De-Meulder^24^, Sven Erik-Dahlen^25^, Ian M Adcock^5^, Kian. Kian Fan Chung^5^, Loems Ziegler-Heitbrock^26^, Peter J. Sterk^27^, Dave Singh^28^, Christopher Brightling^1^ on behalf of the U-BIOPRED and the EvA study teams.

^^

^1^ Institute for Lung Health, Leicester NIHR Biomedical Research Centre, University of Leicester, Leicester, UK

^2^ GSK Respiratory Therapeutic Area Unit, Stevenage, UK

^3^ CNAG-CRG Centre Nacional d’Anàlisi Genòmica, Centre for Genomic Regulation, Barcelona Institute for Science and Technology, Barcelona, Spain.

^4^ Psychiatric Genetics Unit, Group of Psychiatry, Mental Health and Addiction, Vall d'Hebron Research Institute (VHIR), Universitat Autònoma de Barcelona, Barcelona, Spain; Biomedical Network Research Centre on Mental Health (CIBERSAM), Instituto de Salud Carlos III, Barcelona, Spain

^5^ Airway Disease Section, National Heart & Lung Institute, Imperial College London, London, United Kingdom. Imperial College, UK

^6^ Data Science Institute, Imperial College London, London, United Kingdom. Imperial College, UK

^7^ Biosci Consulting, Belgium

^8^ CNG Centre National de Génotypage, Institut de Génomique, CEA, Evry, France

^9^ Dept of Pneumology, University Medical Center, Freiburg, Germany.

^10^ Dept of Medical Sciences, University of Ferrara and Ferrara City Hospital, Ferrara, Italy.

^11^ Dept of Respiratory Medicine, University Hospitals Coventry and Warwickshire NHS Trust,

Coventry, UK.

^12^ Dept of Respiratory Medicine, National Institute of Tuberculosis and Lung Diseases, Warsaw, Poland.

^13^ Dept of Pathophysiology, National Koranyi Institute for TB and Pulmonology, Budapest, Hungary.

^14^ Fraunhofer Institute for Toxicology and Experimental Medicine, Hannover, Germany.

^15^ Dept of Medicine, Pulmonary and Critical Care Medicine, University Medical Center Giessen and Marburg, Philipps-Universität Marburg, Germany, Member of the German Center for Lung Research (DZL).

^16^ University of Groningen, University Medical Center Groningen, Dept of Pulmonary Diseases, Groningen, The Netherlands.

^17^ University of Leiden, Leiden University Medical Center, Dept of Pulmonary Diseases, Leiden, The Netherlands.

^18^ University of Groningen, University Medical Center Groningen, Dept of Pathology and Medical Biology, Groningen, The Netherlands.

^19^ University of Oxford, UK

^20^ Department of Medicine, University of Pittsburgh, Pittsburgh, PA, USA; Department of Immunology, University of Pittsburgh, Pittsburgh, PA, USA.

^21^ Department of Medical Sciences, Clinical Chemistry, Uppsala University, Uppsala , Sweden.

^22^ Universitat Pompeu Fabra, Barcelona, Spain

^23^ NIHR Southampton Respiratory Biomedical Research Unit and Clinical and Experimental Sciences, Southampton, UK

^24^ European Institute for Systems Biology and Medicine (EISBM), CNRS-ENS-UCBL, Université de Lyon, Lyon cedex 07, France

^25^ Karolinska Institute, Stockholm, Sweden,

^26^ EvA Study Center, Helmholtz Zentrum Muenchen and Asklepios-Klinik, Gauting, Germany.

^27^ Dept. Respiratory Medicine, Amsterdam University Medical Centres, University of Amsterdam, Amsterdam, The Netherlands

^28^ Centre for Respiratory Medicine and Allergy, The University of Manchester, Medicines Evaluation Unit, University Hospital of South Manchester NHS Foundation Trust, Manchester, UK

* Denotes first authors with equal contribution

**Corresponding Author:**

Professor CE Brightling

University of Leicester

Glenfield General Hospital

Leicester, LE3 9QP, U.K.

Tel.: 0044 116 258 3998, Fax: 0044 1162502787, E-mail: [ceb17@le.ac.uk](mailto:ceb17@le.ac.uk)
